# Enantioselective Synthesis, Crystal Structures, and Stereoisomerism of Substituted *o*,*m*,*o*,*p*-Tetraphenylenes

**DOI:** 10.1021/acs.orglett.4c02712

**Published:** 2024-09-10

**Authors:** Yuya Kawai, Tomohiro Oriki, Yu Sato, Juntaro Nogami, Yoshinobu Kamiya, Shunsuke Suzuki, Ken Tanaka

**Affiliations:** Department of Chemical Science and Engineering, Tokyo Institute of Technology, O-okayama, Meguro-ku, Tokyo 152-8550, Japan

## Abstract

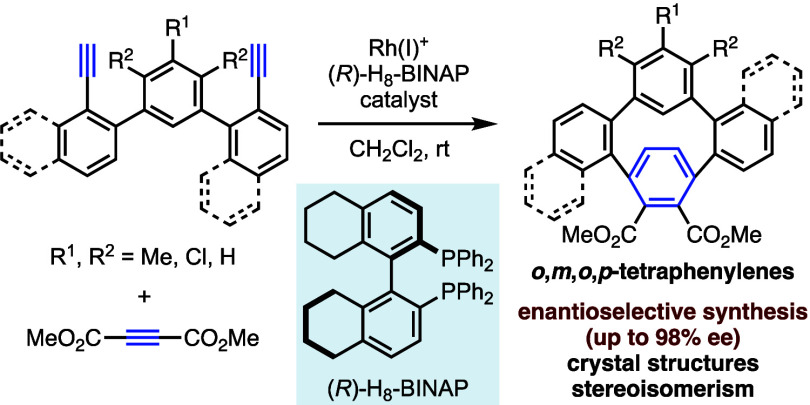

We have achieved the enantioselective synthesis of highly strained, substituted *o*,*m*,*o*,*p*-tetraphenylenes (≤98% ee) via the cationic Rh(I)/(*R*)-H_8_-BINAP complex-catalyzed chemo-, regio-, and enantioselective intermolecular cross-[2+2+2] cycloaddition of teraryl diynes with dimethyl acetylenedicarboxylate. X-ray crystallographic analyses demonstrate the highly bent structures of the *para*-substituted benzene moieties, and density functional theory calculations reveal the large local strain of the paraphenylene unit. ^1^H nuclear magnetic resonance analyses and theoretical calculations elucidate the stereoisomerism, indicating that the nonrotatable *ortho*-disubstituted biphenyl structure results in *cis* and *trans* isomers.

*o*,*o*,*o*,*o*-Tetraphenylene^1^ is a cyclic aromatic hydrocarbon with a stable saddle-shaped structure, in which all four benzene rings are linked at the *ortho* position. As shown in the top of Figure 1a, introducing a substituent R at the *ortho* position of the benzene ring results in axially chiral tetraphenylene **A** due to the nonrotatable *ortho*-trisubstituted biphenyl structure.^1c^ Molecules **B** (*o*,*o*,*o*,*m*-tetraphenylene) and **C** (*o*,*o*,*o*,*p*-tetraphenylene^2^), in which one of the benzene rings is linked to the *meta* or *para* positions, have never been synthesized, probably because of high strain and instability (Figure 1a, top). In contrast, the *meta* linkage of the two benzene rings stabilizes the molecule (*o*,*m*,*o*,*m*-tetraphenylene^3^) without distortion. In this molecule, the two *meta*-linked benzene rings cannot rotate around each other due to steric hindrance, and as shown in the bottom of Figure 1a, molecule **D** with substituent R introduced at the *ortho* position of the benzene ring should become axially and planarly chiral due to two rotational restrictions, resulting in enantiomers and diastereomers (*cis*/*trans*-isomers). Rajca et al. reported the enantioselective synthesis of *o*,*o*,*o*,*o*-tetraphenylenes by (−)-Sparteine-induced lithiation of 2,2′-dibromobiphenyl followed by the CuBr_2_-mediated enantioselective coupling.^4^ Subsequently, Shibata et al. reported the catalytic enantioselective synthesis of *o*,*o*,*o*,*o*-tetraphenylenes^5^ and *o*,*m*,*o*,*m*-tetraphenylenes^6^ by the Rh(I)-catalyzed intermolecular homo-[2+2+2] cycloaddition reactions^7^ of triynes.

**Figure 1:**
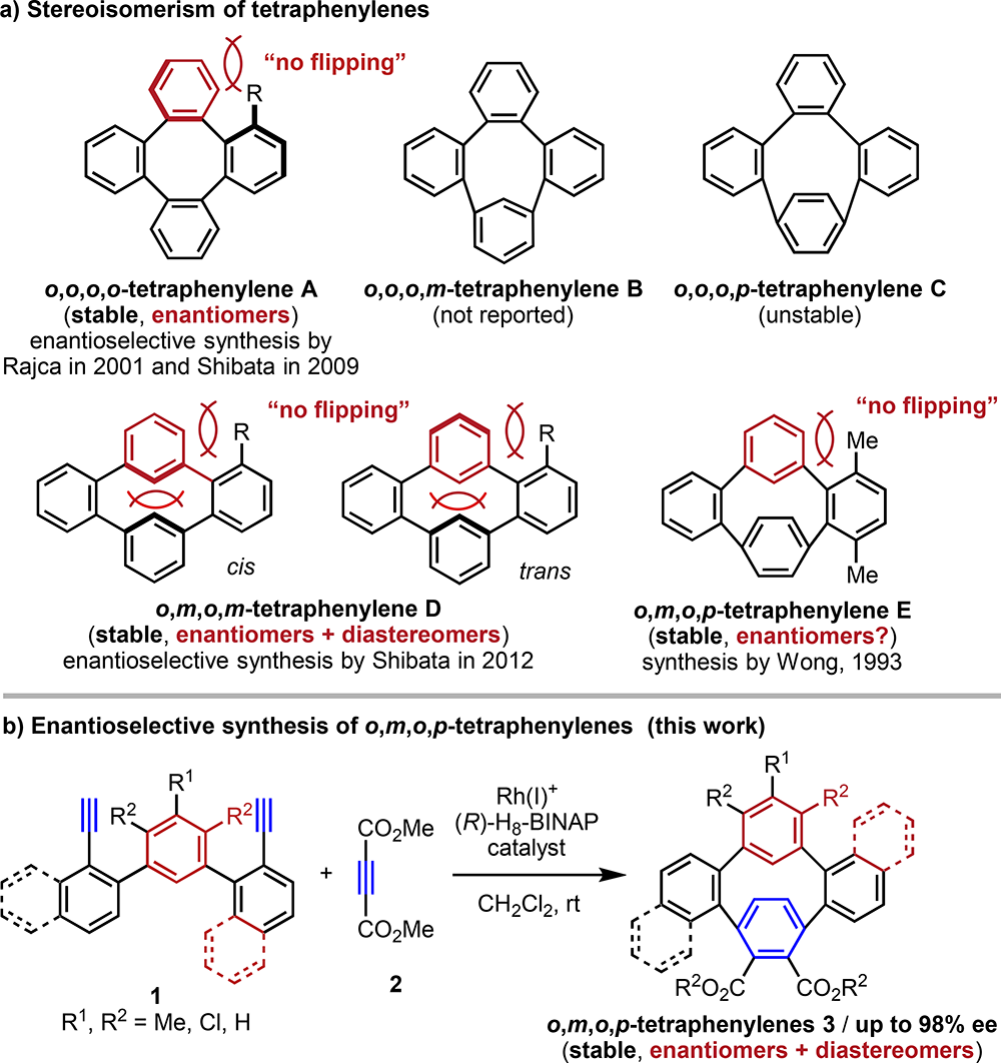
Synthesis and stereoisomerism of tetraphenylenes.

On the other hand, linking the two benzene rings at the *meta* and *para* positions significantly increases the distortion. In 1988, Wong et al. first synthesized *o*,*m*,*o*,*p*-tetraphenylene,^8^ followed in 1993, they reported the synthesis of *o*,*m*,*o*,*p*-tetraphenylene **E**, in which two methyl groups were introduced at the *ortho* positions of the benzene ring (Figure 1a, bottom).^9^ In molecule **E**, the *meta*- and *para*-linked benzene rings cannot rotate due to steric repulsion and ring strain, resulting in axial chirality, which was expected to give rise to enantiomers. However, isolation of the enantiomers was unsuccessful.^9^

Here, we have succeeded in the synthesis of highly strained, enantioenriched *o*,*m*,*o*,*p*-tetraphenylenes **3** (up to 98% ee) via the Rh(I)-catalyzed chemo-, regio-, and enantioselective intermolecular cross-[2+2+2] cycloaddition of teraryl diynes with dimethyl acetylenedicarboxylate (**2**) (Figure 1b).^10^ Their structures were determined by X-ray crystallographic analyses, and their stereoisomerism was confirmed by ^1^H NMR analysis and theoretical calculations.

In 2003, we reported that a cationic Rh(I)/H_8_-BINAP complex catalyzes the chemo- and regioselective [2+2+2] cycloaddition of two molecules of terminal alkynes involving phenylacetylene with one molecule of dialkyl acetylenedicarboxylate at room temperature to produce the corresponding 3,6-disubstituted phthalate in high yield.^11^ Thus, we investigated the enantioselective synthesis of *o*,*m*,*o*,*p*-tetraphenylenes **3** using dimethyl acetylenedicarboxylate (**2**) and the cationic Rh(I)/(*R*)-H_8_-BINAP catalyst (10–40 mol % Rh), as shown in Figure 2, applying various substituted terphenyl diynes **1** instead of two phenylacetylenes to this reaction. The desired *o*,*m*,*o*,*p*-tetraphenylene **3** is highly strained, and thus we also assumed the formation of low-strain regioisomer **4**. In addition, on the basis of the ^1^H NMR analysis of molecule **E** by Wong et al., *cis*-**3** and *trans*-**3** should exist if the rotation of the *meta*-linked benzene ring is inhibited by substituents.^9^

**Figure 2:**
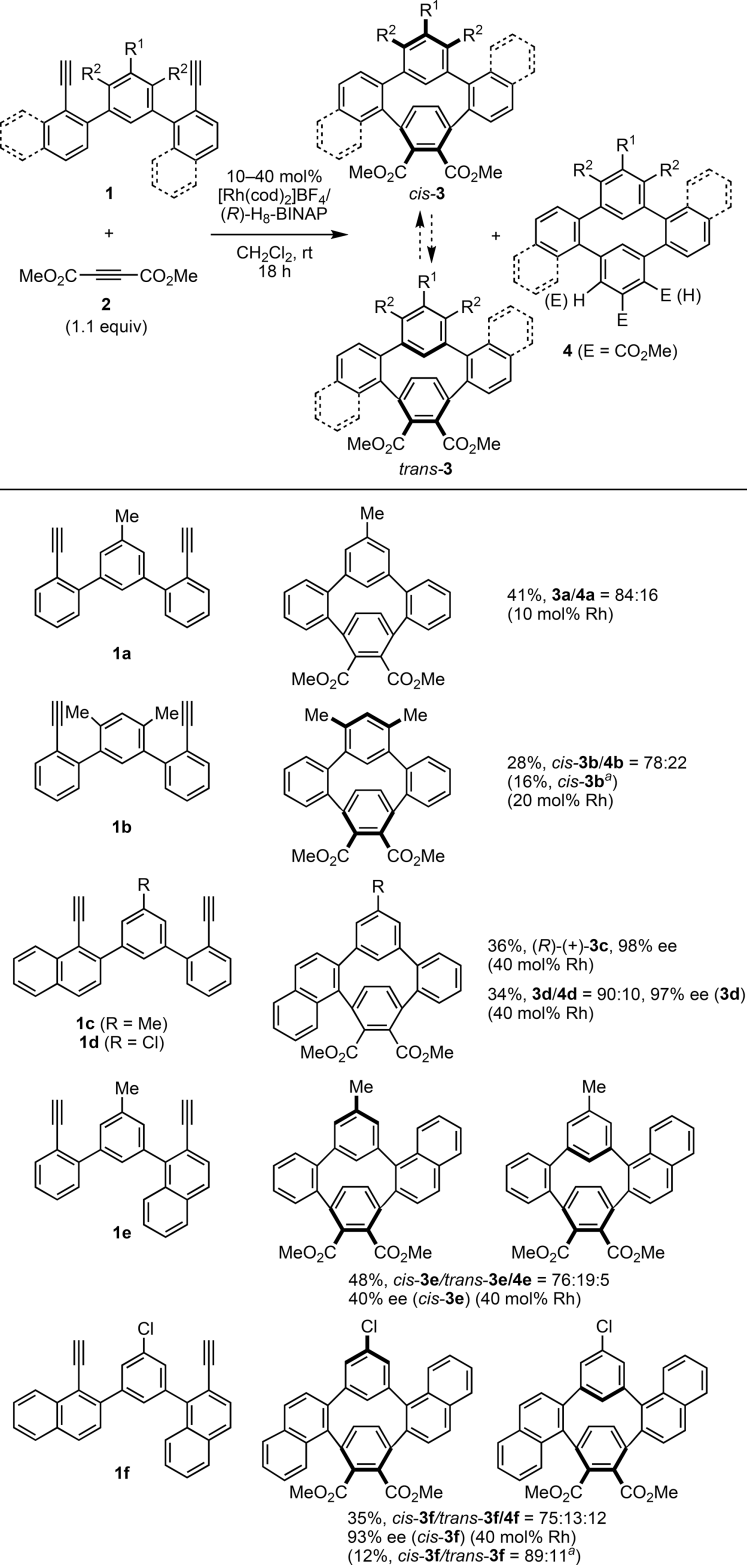
Rh-catalyzed [2+2+2] cycloaddition of terphenyl diynes **1** with dimethyl acetylenedicarboxylate (**2**). Yields are of the isolated products. [Rh(cod)_2_]BF_4_ (10–40 mol %), (*R*)-H_8_-BINAP (10–40 mol %), **1** (0.062–0.10 mmol), **2** (1.1 equiv), and CH_2_Cl_2_ (0.025 M for **1a** and **1b** and 0.0125 M for **1c**–**f**) were used. ^*a*^Isolated yield after repeated silica gel PTLC.

As expected, symmetrically substituted terphenyl diynes **1a** and **1b** reacted with **2** to give the desired *o*,*m*,*o*,*p*-tetraphenylenes **3a** and **3b** along with the regioisomeric *o*,*m*,*o*,*m*-tetraphenylenes **4a** and **4b** in 41% and 28% yields with 84:16 and 78:22 ratios of **3**/**4**, respectively. Although *cis-***3b** and *trans-***3b** can exist due to rotational inhibition by the methyl groups, only *cis-***3b** was produced in this reaction. From a mixture of *cis*-**3b** and **4b**, pure *cis*-**3b** could be isolated after recrystallization. Unsymmetric 1-ethynylnaphthalene-derived diynes **1c** and **1d** reacted with **2** to yield chiral tetraphenylenes **3c** and **3d** with high regio- and enantioselectivity (97–98% ee). Unsymmetric 2-ethynylnaphthalene-derived diyne **1e** reacted with **2** to give a mixture of *cis*-**3e**, *trans*-**3e**, and **4e** in 48% yield due to rotational inhibition by the naphthyl group in **3e**. In contrast to **3c**, the ee value of *cis*-**3e** markedly dropped to 40%. Interestingly, the reaction of a hybrid diyne **1f** bearing both 1-ethynyl- and 2-ethynylnaphthalene moieties with **2** also afforded a mixture of *cis*-**3f**, *trans*-**3f**, and **4f** in 35% yield, but the ee of *cis*-**3f** improved to 93%. From this mixture, *cis*/*trans*-**3f** could be isolated by repeated silica gel preparative thin-layer chromatography (PTLC). ^1^H NMR of the methyl group of the ester moiety demonstrated the interconversion of *cis-***3** and *trans*-**3**. Broad peaks of the methyl groups were observed in **3a**, **3c**, and **3d**; thus, the *cis-* and *trans*-forms interconvert at room temperature. In contrast, in **3b**, **3e**, and **3f**, the peaks of the methyl groups corresponding to the isomers were observed sharply, and thus the *cis-*form and *trans*-form do not interconvert at room temperature. The thermal stability of the stereochemistry was evaluated for **3e**. Heating a (CH_2_Cl)_2_ solution of **3e** (*cis/trans* = 4:1, 40% ee) at 80 °C for 13 h resulted in slight epimerization but not racemization to give **3e** (*cis/trans* = 3:1, 41% ee). Heating at 150 °C in dimethyl sulfoxide resulted in complete decomposition, and thus we performed DFT calculations to determine the racemization barrier of **3e**. However, the barrier was significantly higher and did not converge.

Although we failed to obtain chiral single crystals of (+)-**3c**, the sign and value of experimental specific rotation of (+)-**3c** obtained from **1c** and **2** with (*R*)-H_8_-BINAP as a ligand agrees well with those of the theoretical specific rotation of (*R*)-**3c** [B3LYP/6-311++G(2d,2p)] (Table S27), confirming the *R* absolute configuration of (+)-**3c**. This enantioselection can be rationalized by the preferential reaction of **INT-A** rather than **INT-B** from **1c**, **2**, and (*R*)-H_8_-BINAP due to avoiding steric repulsion between the naphthyl group (colored in blue) and the equatorial phenyl group (colored in red) on the ligand in **INT-B** (Figure 3a). In diyne **1c**, the 1-ethynylnaphthalene moiety is more sterically hindered than the phenylacetylene moiety, and therefore, the phenylacetylene moiety in **1c** selectively reacts with **2** and Rh to give **INT-A** or **INT-B**. However, the difference in steric hindrance between the 2-ethynylnaphthalene (colored in gray) and phenylacetylene moieties in diyne **1e** is small. Accordingly, the reactions through **INT-C** and **INT-D**, giving opposite enantiomeric products, are not biased, which would reduce the ee value of the product (Figure 3b).

**Figure 3:**
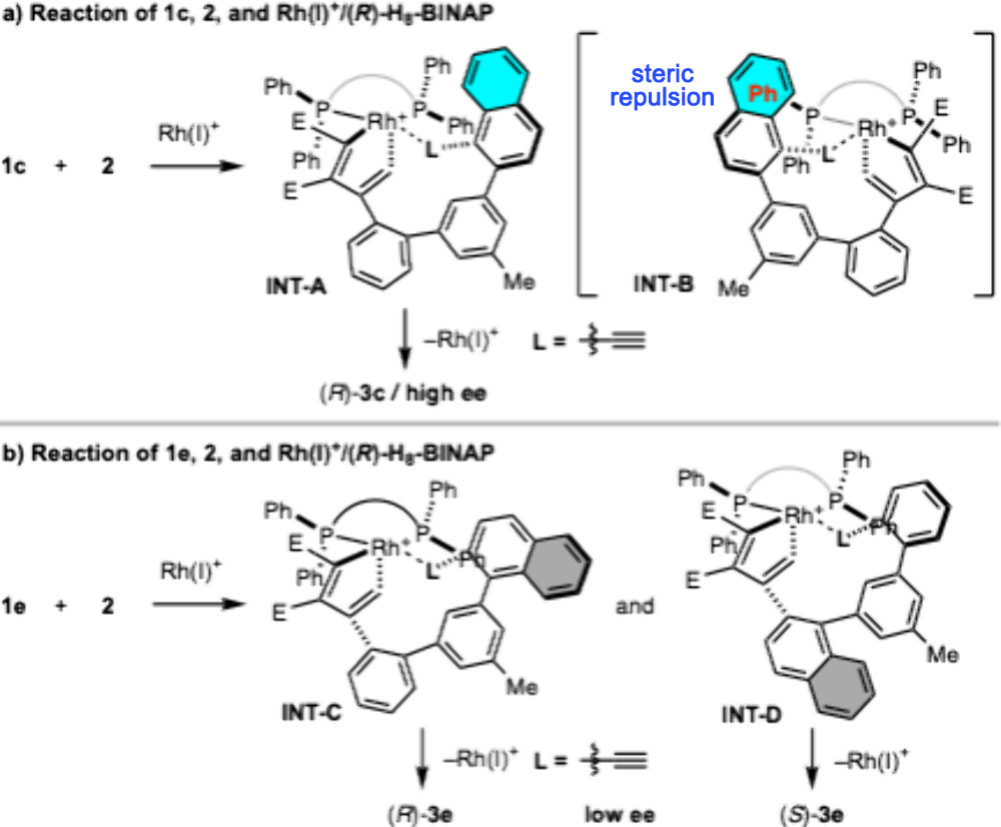
Plausible enantioselection mechanisms using (*R*)-H_8_-BINAP.

We performed single-crystal X-ray diffraction analyses of *o*,*m*,*o*,*p*-tetraphenylenes **3a** (Figures S1–S3), (±)-*cis*-**3b** (Figure S4), (±)-**3d** (Figure S5), (±)-*cis*-**3e** (Figure S6), and (±)-*cis*-**3f** (Figure 4). *cis*-**3a**,**d** and *trans*-**3a**,**d** are interconvertible in solution at room temperature, crystals with a *cis*- to *trans*-isomer ration of 1:1 were obtained for **3a**, whereas crystals of only the *cis*-isomer were obtained for **3d**. For (±)-*cis*-**3b**, (±)-*cis*-**3e**, and (±)-*cis*-**3f**, their *cis* structures were confirmed unambiguously. As a representative example, X-ray crystal structures of (±)-*cis*-**3f** is shown in Figure 4. The top view shows that the two benzene rings partially overlap, and the side view shows that the *para*-linked benzene ring is curved significantly. Measurement of the distance between the two planes shows that the shortest interplane distance (2.72 Å) is shorter than that of *o*,*p*,*o*,*p*-tetraphenylene (2.80 Å).^12^ The benzene ring bonded to naphthalene at the *meta* position (highlighted in blue) is tilted 19.5° relative to the benzene ring bonded to naphthalene at the para position (highlighted in orange). The deformation of the benzene ring from planarity can be defined by the deviation angles (α/α′) of the *para* carbons from the base plane of the boat-shaped benzene ring (Table 1).^13^ The average deviation angles (17.9–19.2°) of *cis*-**3** are larger than those of unsubstituted *o*,*m*,*o*,*p*-tetraphenylene (17.2°),^9^ and [5]cycloparaphenylene (CPP, 15.6°),^14^ indicating that the present substituted *o*,*m*,*o*,*p*-tetraphenylenes **3** are highly distorted cyclophenylene molecules.

**Figure 4:**
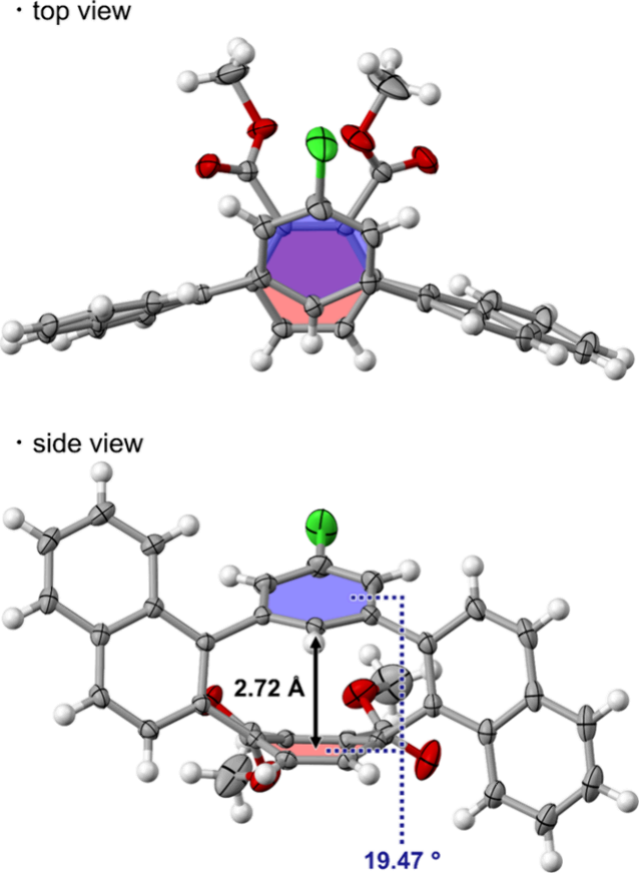
X-ray crystal structures of (±)-*cis*-**3f**, showing thermal ellipsoids at the 50% probability level. The distance between the red plane and the edge carbon atom of the blue plane is indicated in black. The dihedral angle between the red plane and the blue plane is indicated in blue.

**Table 1:**
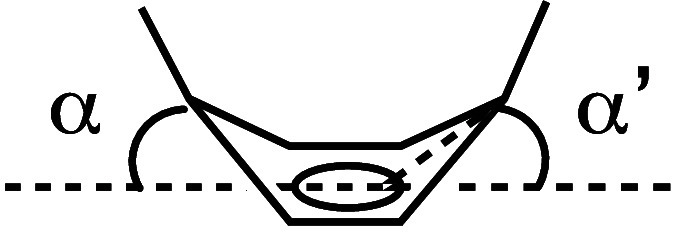
Deviation Angles of **3** and Related Compounds

compound	α/α′	average
*cis*-**3a** (*trans*-**3a**)	17.6/18.6, 18.5/19.0 (16.8/17.6, 16.1/16.8)	18.4 (16.8)
*cis*-**3b**	17.8/17.9	17.9
(±)-**3d**	18.8/19.5	19.2
(±)-*cis*-**3e**	17.8/18.7	18.3
(±)-*cis*-**3f**	18.1/19.0	18.6
*o*,*m*,*o*,*p*-tetraphenylene^a^	16.7/17.7	17.2
[5]CPP^b^	15.6	15.6

a Data from ref (9).

b Data from ref (14).

We further evaluated the strain energies by DFT calculations. The strain energies of simplified *cis*-**3a′** (Figure S7), *cis*-**3c′** (Figure S8), *cis*-**3e′** (Figure S9), and *cis*-**3f′** (Figure 5) by removing substituents on the *meta*-linked benzene ring, based on the homodesmotic reaction method,^15^ are 18.3, 16.5, 17.9, and 16.2 kcal/mol, respectively, indicating that *cis*-**3a** is the most strained molecule (Table 2). For *trans*-**3′**, **3a′** and **3e′** are the most strained molecules. These values are markedly smaller than [2.2]paracyclophane (37 kcal/mol) and [6]CPP (91 kcal/mol).^16^ The StrainViz^16^ examination of the strain distribution for *cis*-**3a′** (Figure S7), *cis*-**3c′** (Figure S8), *cis*-**3e′** (Figure S9), and *cis*-**3f′** (Figure 5) shows that the paraphenylene unit of *cis*-**3e′** is the largest with 3.98 kcal/mol, which is less than the 4.47 kcal/mol of [6]CPP^16^ but more than the 3.39 kcal/mol of [2.2]paracyclophane.^16^

**Table 2:**
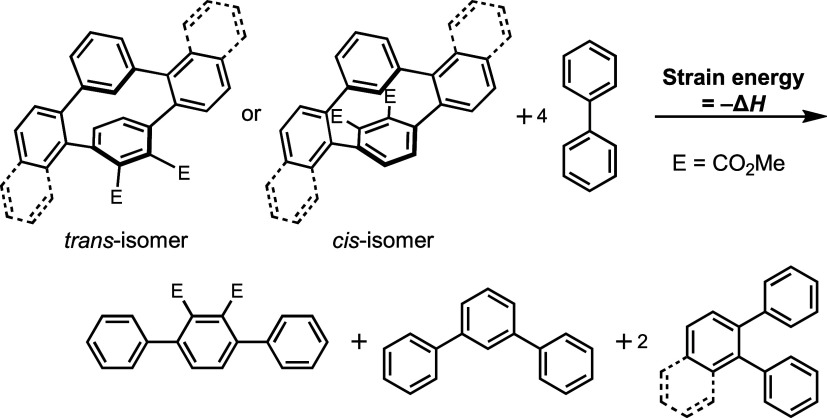
Strain Energies of **3a′**, **3c′**, **3e′**, and **3f′** Determined by DFT Calculations at the B3LYP/6-31G(d) Level of Theory

compound	*cis-*isomer (kcal/mol)	*trans-*isomer (kcal/mol)
**3a′**	18.3	18.5
**3c′**	16.5	16.5
**3e′**	17.9	18.5
**3f′**	16.2	16.5

**Figure 5:**
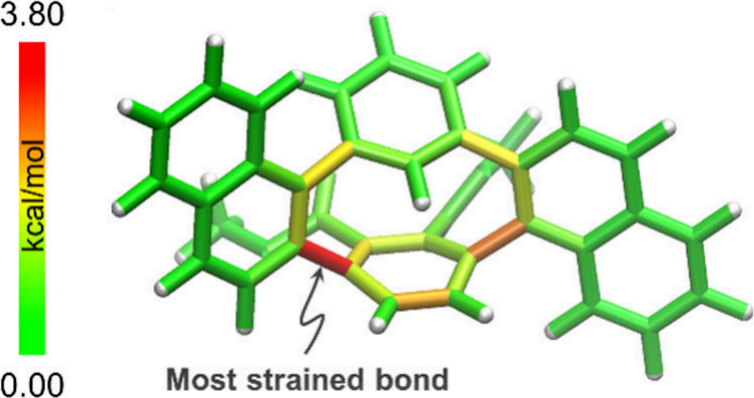
StrainViz of *cis*-**3f′** by DFT calculations at the B3LYP/6-31G(d) level of theory.

^1^H NMR spectra of **3a**, **3c**, **3e**, and **3f** show that the *cis*/*trans* isomers of **3a** and **3c** interconvert at room temperature, but the *cis*/*trans* isomers of **3e** and **3f** do not. The *meta*-linked biphenyl (highlighted in red, Figure 6a, left) in **3a** and **3c** is *ortho*-monosubstituted and would easily rotate at room temperature. On the other hand, the *meta*-linked biphenyl (highlighted in blue, Figure 6a, right) in **3e** and **3f** is *ortho*-disubstituted and would normally rotate readily at room temperature. However, the proximity of H^a^ and H^b^ due to ring strain may inhibit the rotation of **3e** and **3f**. Thus, we performed DFT calculations to determine the rotational barriers of *cis*/*trans* isomerization for simplified compounds **3a′**, **3c′**, **3e′**, and **3f′**. The DFT calculations showed that the activation energy for rotation of the biphenyl unit in **3a′** and **3c′** are Δ*G*^‡^ = 15.1 and 16.1 kcal/mol, respectively, indicating that *cis*/*trans* isomerization proceeds rapidly even at room temperature (Figure 6b, top). In contrast, the activation energy for rotation of the biphenyl unit in **3e′** and **3f′** are Δ*G*^‡^ = 25.4 kcal/mol, indicating that *cis*/*trans* isomerization does not proceed at room temperature (Figure 6b, bottom). Accordingly, for *o*,*m*,*o*,*p*-tetraphenylenes, the *ortho*-disubstituted biphenyl cannot rotate at room temperature.

**Figure 6:**
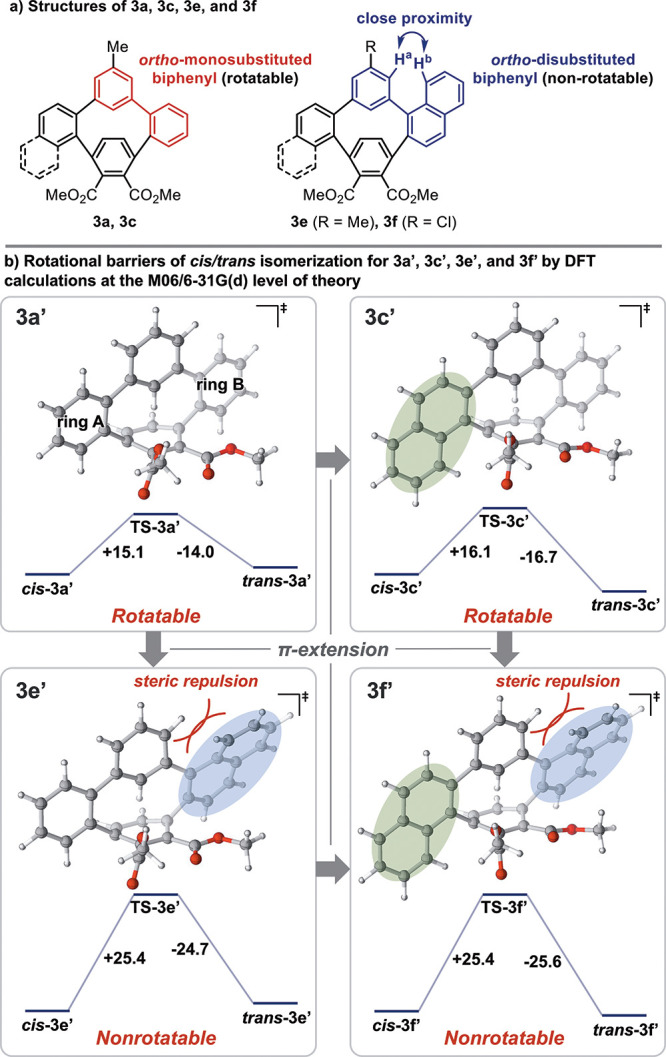
Stereoisomerism of **3** and **3′**.

In conclusion, we have achieved the enantioselective synthesis of highly strained, substituted *o*,*m*,*o*,*p*-tetraphenylenes **3** with up to 98% ee via the cationic Rh(I)/(*R*)-H_8_-BINAP complex-catalyzed chemo-, regio-, and enantioselective intermolecular cross-[2+2+2] cycloaddition of teraryl diynes **1** with dimethyl acetylenedicarboxylate (**2**). In diyne **1c**, the 1-ethynylnaphthalene moiety is more sterically hindered than the phenylacetylene moiety, and thus the phenylacetylene moiety of **1c** may react selectively with **2** and Rh to afford **3c** with high enantioselectivity. X-ray crystallographic analyses of **3** revealed the highly bent structures of the *para*-substituted benzene moieties, and their deviation angles are larger than those of unsubstituted *o*,*m*,*o*,*p*-tetraphenylene and [5]CPP. DFT calculations of **3** revealed that the total strain energies are smaller than those of [2.2]paracyclophane and [6]CPP. Nevertheless, the local strain of the most distorted paraphenylene unit of *cis*-**3a′** is smaller than [6]CPP but larger than [2.2]paracyclophane. ^1^H NMR analyses and theoretical calculations elucidated the stereoisomerism. The *ortho*-disubstituted biphenyl structure results in *cis* and *trans* isomers because this moiety cannot rotate at room temperature. Thus, we have demonstrated the enantioselective synthesis of substituted *o,m,o,p*-tetraphenylenes **3** and their structures and stereoisomerism.

## Data Availability

The data underlying this study are available in the published article and its Supporting Information.

## Supporting Information Available

Detailed experimental procedures, characterization data, crystal data, theoretical calculations, chiral HPLC charts, and NMR spectra of the compounds (PDF)
