# Supplementary material for: Enantioselective Synthesis, Crystal Structures, and Stereoisomerism of Substituted o,m,o,p-Tetraphenylenes
Source: Org Lett. 2024 Sep 10;26(37):7869–74. doi: 10.1021/acs.orglett.4c02712 (PMC11421083; doi:10.1021/acs.orglett.4c02712)

Supporting Information

Enantioselective Synthesis, Crystal Structures, and Stereoisomerism of Substituted *o*,*m*,*o*,*p*-Tetraphenylenes

Yuya Kawai,[1,‡] Tomohiro Oriki,[1,‡] Yu Sato,[1] Juntaro Nogami,[1] Yoshinobu Kamiya,[1] Shunsuke Suzuki,[1] and Ken Tanaka*,[1]

[1] Department of Chemical Science and Engineering, Tokyo Institute of Technology, O-okayama, Meguro-ku, Tokyo 152-8550, Japan

‡ Contributed equally

Table of Contents

1. Materials and Methods **S2**

1.1. General Experimental Information **S2**

1.2. General Analytical Information **S2**

2. Synthetic Experiments **S3–16**

2.1. Synthesis of Diynes **S3–S11**

2.2. Synthesis of *o*,*m*,*o*,*p*-Tetraphenylenes **S12–S16**

3. X-Ray Single Crystal Diffraction Analyses **S17–S26**

4. Theoretical Calculations **S27–S45**

5. Determination of Absolute Configuration of (+)-3c **S46–S51**

6. References **S52–S53**

7. Chiral HPLC Charts **S54–S61**

8. ^{1}H and ^{13}C NMR Spectra **S62–S83**

1. General

1.1. General Experimental Information

Dry-degassed CH_2Cl_2 (No. 041-32345) for the synthesis of **3a**–**f** and **4a**–**f** was obtained from Wako Pure Chemical Industries and used as received. THF for the synthesis of **S17** and **1a**–**f**, DMSO for the synthesis of **S9**, **S13**, and **S20**, 1,4-dioxane for the synthesis of **S9** and **S20**, CH_2Cl_2 for the synthesis of **S7**, Et_2O for the synthesis of **S16**, and toluene for the synthesis of **S16** were dried over Molecular Sieves 4A. $[Rh(cod)_2]BF_4$ was obtained from Umicore AG. H_8-BINAP was obtained from Takasago International Corporation. (2-((Triisopropylsilyl)ethynyl)phenyl)boronic acid (**S2**),[1] 1-((triisopropylsilyl)ethynyl)naphthalen-2-ol (**S6**),[2] and 1-bromonaphthalen-2-yl trifluoromethanesulfonate (**S15**)[3] were prepared according to the literature. Commercially available reagents were purchased from TCI Chemicals, Wako Pure Chemical Industries, Sigma-Aldrich, and Kanto Chemicals and used as received unless otherwise noted. Silica gel column chromatography was performed using silica gel [Silica Gel 60 N (spherical, neutral), Kanto Chemicals] and JIS (Japanese Industrial Standards) special-grade solvents. Silica gel preparative thin-layer chromatography (PTLC) was performed using silica gel (Wakogel® B-5F) and JIS special-grade solvents. All reactions were carried out under an atmosphere of argon or nitrogen in oven-dried glassware with magnetic stirring. For reactions that require heating, an oil bath was used as the heat source.

1.2. General Analytical Information

^{1}H and ^{13}C NMR data of new compounds were collected on a Bruker AVANCE III HD 400 spectrometer at ambient temperature. All ^{1}H NMR experiments are reported in δ units, parts per million (ppm), and were measured relative to the signals for residual chloroform (7.26 ppm). All ^{13}C NMR spectra are reported in ppm relative to deuterochloroform (77.01 ppm) and were obtained with ^{1}H decoupling. HRMS data were obtained on a Bruker micrOTOF Focus II. Melting points were determined on a Mettler MP50 and were uncorrected. Chiral HPLC analyses were performed on a JASCO HPLC 2000 series. Optical rotation values were measured on a JASCO P-2200. Electronic circular dichroism (ECD) spectra were obtained on a JASCO J-820 spectrometer. Single crystal X-ray diffraction data were collected using an XtaLAB mini II diffractometer with graphite monochromated Mo-Kα radiation.

2. Synthetic Experiments

2-1. Synthesis of Diynes

2,2''-Diethynyl-5'-methyl-1,1':3',1''-terphenyl (1a)

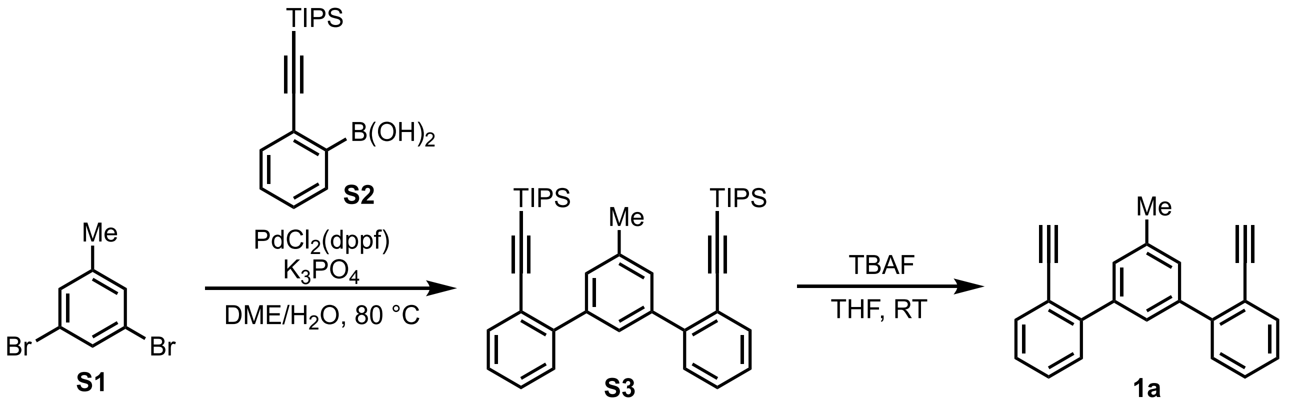

A solution of **S1** (2.50 g, 10.0 mmol), **S2**[1] (6.20 g, 20.5 mmol), $PdCl_2$(dppf) (0.0732 g, 0.100 mmol), and K_3PO_4 (6.37 g, 30.0 mmol) in degassed 1,2-dimethoxyethane (DME)/H_2O (4:1, 150 mL) was stirred at 80 °C for 3 h. The reaction mixture was diluted with water and extracted with ethyl acetate (100 mL x 3). The organic layer was washed with brine, dried over Na_2SO_4, filtered, and concentrated. The residue was passed through silica gel column chromatography (eluent: *n*-hexane) to give crude **S3**, which was used in the next step without further purification.

To a solution of the crude **S3** in THF (40 mL) was added tetrabutylammonium fluoride (TBAF, 20.5 mL, 20.5 mmol, 1.0 mol/L in THF) at 0 °C. After stirring at room temperature for 30 min, the reaction mixture was diluted with water and extracted with CH_2Cl_2 (100 mL x 3). The organic layer was washed with brine, dried over Na_2SO_4, filtered, and concentrated. The residue was purified by silica gel column chromatography (eluent: *n*-hexane/EtOAc = 4:1) to give **1a** (1.90 g, 6.51 mmol, 65% yield).

Yellow solid; mp 112.1–113.2 °C; ^{1}H NMR ($CDCl_3$, 400 MHz) δ 7.63–7.61 (m, 2H), 7.58 (dd, J = 1.5,1.5 Hz, 1H), 7.44–7.38 (m, 6H), 7.29 (ddd, J = 7.5, 7.1, 1.8 Hz, 2H), 3.07 (s, 2H), 2.46 (s, 3H); ^{13}C NMR ($CDCl_3$, 100 MHz) δ 144.4, 139.9, 137.2, 133.9, 129.7, 129.2, 129.0, 127.4, 126.9, 120.4, 83.3, 80.2, 21.5; HRMS (ESI) calcd for $C_{23}H_{17}$ $[M+H]^+$ 293.1325 found 293.1327.

2,2''-Diethynyl-4',6'-dimethyl-1,1':3',1''-terphenyl (1b)

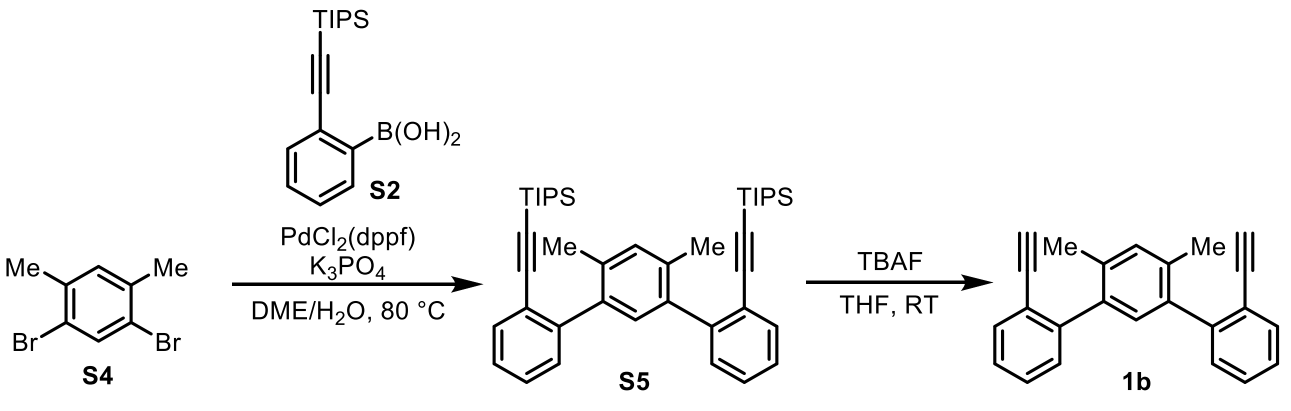

A solution of **S4** (0.264 g, 1.00 mmol), **S2**[1] (0.635 g, 2.10 mmol), $PdCl_2$(dppf) (7.3 mg, 0.010 mmol), and K_3PO_4 (0.425 g, 2.00 mmol) in degassed DME/H_2O (4:1, 10 mL) was stirred at 80 °C

for 2 h. The reaction mixture was diluted with water and extracted with ethyl acetate (10 mL x 3). The organic layer was washed with brine, dried over Na_2SO_4, filtered, and concentrated. The residue was passed through silica gel column chromatography (eluent: *n*-hexane) to give crude **S5**. This crude **S5** was used for the next reaction without further purification.

To a solution of the crude **S5** in THF (10 mL) was added TBAF (1.4 mL, 1.4 mmol, 1.0 mol/L in THF) at 0 °C. After stirring at room temperature for 30 min, the reaction mixture was diluted with water and extracted with CH_2Cl_2 (10 mL x 3). The organic layer was washed with brine, dried over Na_2SO_4, filtered, and concentrated. The residue was purified by silica gel column chromatography (eluent: *n*-hexane/EtOAc = 4:1) to give **1b** (0.177 g, 0.579 mmol, 58% yield).

White solid; mp 118.2–119.1 °C; ^{1}H NMR ($CDCl_3$, 400 MHz) δ 7.57 (dd, J = 7.6, 0.9 Hz, 2H), 7.36 (ddd, J = 7.5, 7.5, 1.4 Hz, 2H), 7.30–7.25 (m, 4H), 7.17 (s, 1H), 7.08 (s, 1H), 2.94 (s, 2H), 2.21 (s, 6H); ^{13}C NMR ($CDCl_3$, 100 MHz) δ 144.7, 137.4, 135.2, 132.9, 131.3, 131.1, 130.0, 128.5, 126.8, 121.9, 82.9, 79.7, 19.7; HRMS (ESI) calcd for $C_{24}H_{18}Na$ [M+Na]$^+$ 329.1301 found 329.1308.

1-((Triisopropylsilyl)ethynyl)naphthalen-2-yl trifluoromethanesulfonate (S7)

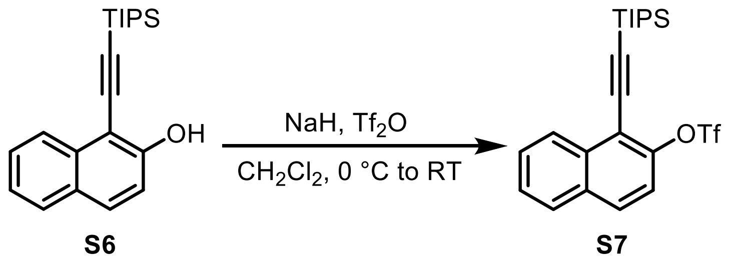

To a solution of **S6**[2] (1.39 g, 4.27 mmol) in CH_2Cl_2 (50.0 mL) were added 55wt% sodium hydride (NaH, 279.5 mg, 6.41 mmol) at 0 °C. After stirring at 0 °C for 30 minutes, trifluoromethanesulfonic anhydride (Tf_2O, 1.51 g, 5.34 mmol) was added, and the mixture was stirred at room temperature for 1 h. The reaction mixture was diluted with water, and extracted with CH_2Cl_2 (100 mL x 3). The organic layer was washed with brine, dried over Na_2SO_4, filtered, and concentrated. The residue was purified by silica gel column chromatography (eluent: *n*-hexane/EtOAc = 10:1) to give **S7** (1.55 g, 3.39 mmol, 79% yield).

Red oil; ^{1}H NMR ($CDCl_3$, 400 MHz) δ 8.41 (dd, J = 8.4, 0.4 Hz, 1H), 7.89–7.85 (m, 2H), 7.67 (ddd, J = 7.0, 7.0, 1.3 Hz, 1H), 7.59 (ddd, J = 6.9, 6.9, 1.3 Hz, 1H), 7.38 (d, J = 9.1 Hz, 1H), 1.30–1.18 (m, 21H); ^{13}C NMR ($CDCl_3$, 100 MHz) δ 148.6, 134.2, 131.9, 130.2, 128.3, 127.4, 126.7, 119.1, 119.1, 118.8 (q, J = 319.1 Hz), 115.3, 105.6, 97.3, 18.7, 11.3; HRMS (ESI) calcd for $C_{22}H_{27}F_3O_3SSiNa$ [M+Na]$^+$ 479.1294 found 479.1285.

((3'-Bromo-5'-methyl-[1,1'-biphenyl]-2-yl)ethynyl)triisopropylsilane (S8)

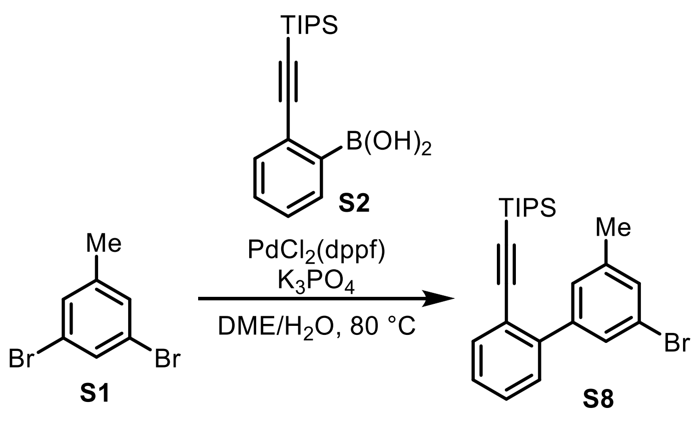

A solution of **S1** (2.50 g, 10.0 mmol), **S2**[1] (1.51 g, 5.00 mmol), PdCl2(dppf) (0.0731 g, 0.100 mmol), and K_3PO_4 (6.37 g, 30.0 mmol) in degassed DME/H_2O (4:1, 100 mL) was stirred at 80 °C for 11 h. The reaction mixture was diluted with water and extracted with ethyl acetate (100 mL x 3). The organic layer was washed with brine, dried over Na_2SO_4, filtered, and concentrated. The residue was passed through silica gel column chromatography (eluent: *n*-hexane) to give **S8** (1.27 g, 2.98 mmol, 60% yield).

Yellow oil; ^{1}H NMR ($CDCl_3$, 400 MHz) δ 7.60–7.58 (m, 1H), 7.50–7.49 (m, 1H), 7.37–7.33 (m, 1H), 7.30–7.27 (m, 4H), 2.35 (s, 3H), 1.03–1.01 (m, 21H); ^{13}C NMR ($CDCl_3$, 100 MHz) δ 142.9, 142.5, 139.5, 133.8, 130.9, 129.3, 129.3, 128.8, 128.4, 127.3, 122.0, 121.8, 105.8, 94.5, 21.2, 18.6, 11.2 HRMS (ESI) calcd for $C_{24}H_{31}BrSiNa$ [M+Na]$^+$ 449.1271 found 449.1263.

Triisopropyl((3'-methyl-5'-(4,4,5,5-tetramethyl-1,3,2-dioxaborolan-2-yl)-[1,1'-biphenyl]-2-yl)ethynyl)silane (S9)

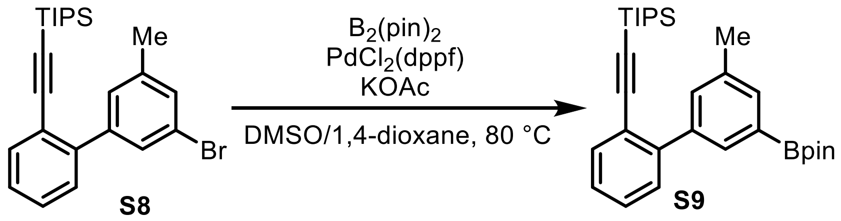

A solution of **S8** (0.822 g, 1.92 mmol), B2pin2 (0.500 g, 1.97 mmol), PdCl2(dppf) (0.0131 g, 0.0179 mmol), and KOAc (0.528 g, 5.37 mmol) in degassed DMSO/1,4-dioxane (1:1, 10 mL) was stirred at 80 °C for 3 h. The reaction mixture was diluted with water and extracted with ethyl acetate (10 mL x 3). The organic layer was washed with brine, dried over Na_2SO_4, filtered, and concentrated. The residue was passed through silica gel column chromatography (eluent: *n*-hexane/EtOAc = 15:1) to give **S9** (0.644 g, 1.36 mmol, 71% yield).

Colorless oil; ^{1}H NMR ($CDCl_3$, 400 MHz) δ 7.73 (s, 1H), 7.60 (s, 1H), 7.58–7.56 (m, 1H), 7.52–7.51 (m, 1H), 7.36–7.30 (m, 2H), 7.27–7.23 (m, 1H), 2.37 (s, 3H),1.34 (s, 12H), 1.00–0.99 (m, 21H); ^{13}C NMR ($CDCl_3$, 100 MHz) δ 144.4, 140.2, 136.5, 134.4, 133.6, 133.0, 132.6, 129.6, 128.2, 126.7, 122.0, 106.4, 93.6, 83.7, 24.9, 21.3, 18.6, 11.2; HRMS (ESI) calcd for $C_{30}H_{43}BO_2SiNa$ [M+Na]$^+$ 497.3018 found 497.3032.

1-Ethynyl-2-(2'-ethynyl-5-methyl-[1,1'-biphenyl]-3-yl)naphthalene (1c)

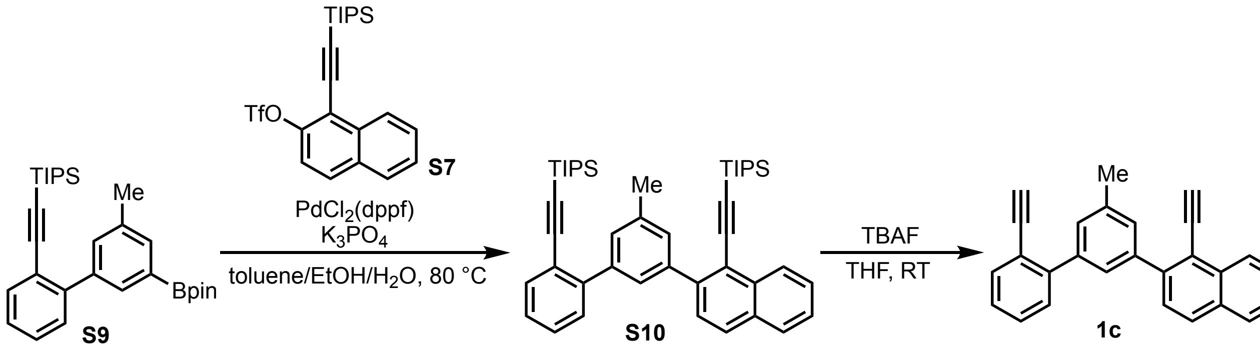

A solution of **S9** (0.237 g, 0.500 mmol), **S7** (0.228 g, 0.500 mmol), PdCl2(dppf) (0.0231 g, 0.0200 mmol), and K_3PO_4 (0.276 g, 2.00 mmol) in degassed toluene/EtOH/H_2O (3:1:1, 10 mL) was stirred at 80 °C for 13 h. The reaction mixture was diluted with water and extracted with ethyl acetate (20 mL x 3). The organic layer was washed with brine, dried over Na_2SO_4, filtered, and concentrated. The residue was passed through silica gel column chromatography (eluent: *n*-

hexane/EtOAc = 4:1) to give crude **S10**. This crude **S10** was used for the next reaction without further purification.

To a solution of the crude **S10** in THF (5 mL) was added TBAF (0.89 mL, 0.89 mmol, 1.0 mol/L in THF) at 0 °C. After stirring at room temperature for 30 min, the reaction mixture was diluted with water and extracted with ethyl acetate (10 mL x 3). The organic layer was washed with brine, dried over Na_2SO_4, filtered, and concentrated. The residue was purified by silica gel column chromatography (eluent: *n*-hexane/EtOAc = 4:1) to give **1c** (0.103 g, 0.301 mmol, 60% yield).

Yellow oil; ^{1}H NMR ($CDCl_3$, 400 MHz) δ 8.50 (d, J = 8.4 Hz, 1H), 7.87 (m, 2H), 7.70 (s, 1H), 7.64–7.59 (m, 2H), 7.58–7.51 (m, 3H), 7.48–7.45 (m, 2H), 7.41 (ddd, J = 7.7, 7.3, 1.3 Hz, 1H), 7.30 (ddd, J = 7.5, 7.4, 1.5 Hz, 1H), 3.49 (s, 1H), 3.08 (s, 1H), 2.49 (s, 3H); ^{13}C NMR ($CDCl_3$, 100 MHz) δ 144.4, 143.5, 140.4, 139.9, 137.2, 134.1, 133.9, 132.1, 129.7, 129.6, 129.3, 129.0, 129.0, 128.1, 127.9, 127.6, 127.3, 127.0, 126.7, 126.4, 120.5, 117.2, 85.6, 83.3, 81.2, 80.3, 21.5; HRMS (APCI) calcd for $C_{27}H_{19}$ $[M+H]^+$ 343.1481 found 343.1474.

((3'-Bromo-5'-chloro-[1,1'-biphenyl]-2-yl)ethynyl)triisopropylsilane (S12)

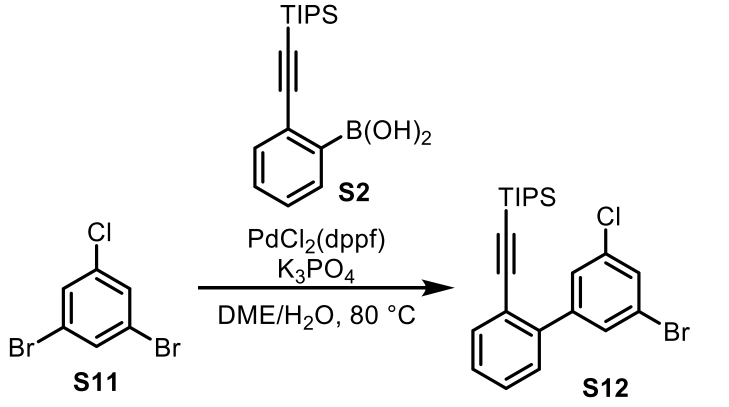

A solution of **S11** (3.39 g, 12.5 mmol), **S2**[1] (1.90 g, 6.27 mmol), $PdCl_2$(dppf) (0.0459 g, 0.0627 mmol), and K_3PO_4 (2.66 g, 12.5 mmol) in degassed DME/H_2O (4:1, 150 mL) was stirred at 80 °C for 4 h. The reaction mixture was diluted with water and extracted with ethyl acetate (200 mL x 3). The organic layer was washed with brine, dried over Na_2SO_4, filtered, and concentrated. The residue was passed through silica gel column chromatography (eluent: *n*-hexane) to give crude **S12** (1.84 g, 4.11 mmol, 66% yield).

Yellow oil; ^{1}H NMR ($CDCl_3$, 400 MHz) δ 7.61–7.59 (m, 2H), 7.51–7.48 (m, 2H), 7.39–7.28 (m, 3H), 1.04–1.03 (m, 21H); ^{13}C NMR ($CDCl_3$, 100 MHz) δ 143.8, 141.3, 134.6, 133.8, 130.6, 130.1, 129.1, 128.6, 128.3, 127.9, 122.2, 122.0, 105.2, 95.4, 18.6, 11.2; HRMS (ESI) calcd for $C_{23}H_{28}BrClSiNa$ $[M+Na]^+$ 469.0724 found 469.0735.

((3'-Chloro-5'-(4,4,5,5-tetramethyl-1,3,2-dioxaborolan-2-yl)-[1,1'-biphenyl]-2-yl)ethynyl)-triisopropylsilane (S13)

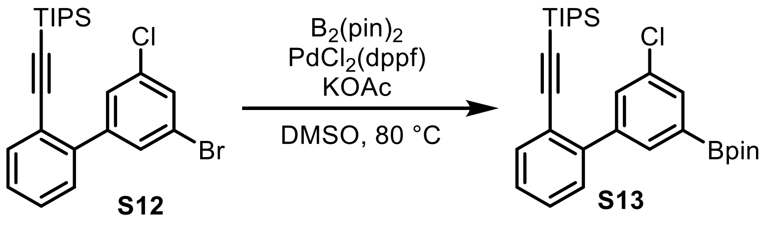

A solution of **S12** (0.448 g, 1.00 mmol), B_2pin_2 (0.267 g, 1.05 mmol), $PdCl_2$(dppf) (0.0146 g, 0.0200 mmol), and KOAc (0.295 g, 3.00 mmol) in degassed DMSO (12 mL) was stirred at 80 °C

for 15 h. The reaction mixture was diluted with water and extracted with ethyl acetate (20 mL x 3). The organic layer was washed with brine, dried over Na_2SO_4, filtered, and concentrated. The residue was passed through silica gel column chromatography (eluent: *n*-hexane/EtOAc = 10:1) to give **S13** (0.482 g, 0.973 mmol, 97% yield).

Colorless oil; ^{1}H NMR ($CDCl_3$, 400 MHz) δ 7.79 (dd, J = 1.6, 1.0 Hz, 1H), 7.74 (dd, J = 2.1, 0.9 Hz, 1H), 7.69 (dd, J = 2.0, 1.8 Hz, 1H), 7.60–7.57 (m, 1H), 7.37–7.26 (m, 3H), 1.34 (s, 12H), 1.02–1.00 (m, 21H); ^{13}C NMR ($CDCl_3$, 100 MHz) δ 142.8, 142.0, 133.6, 133.6, 133.5, 133.4, 132.1, 129.4, 128.4, 127.3, 122.0, 105.7, 94.6, 84.1, 24.9, 18.6, 18.6, 11.2; HRMS (ESI) calcd for $C_{29}H_{40}BClO_2SiNa$ [M+Na]$^+$ 517.2471 found 517.2481.

2-(5-Chloro-2'-ethynyl-[1,1'-biphenyl]-3-yl)-1-ethynylnaphthalene (1d)

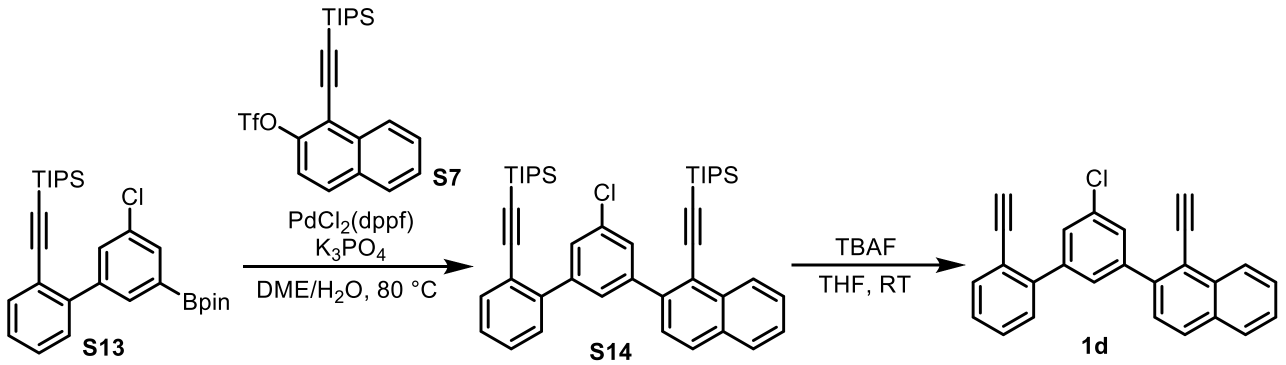

A solution of **S13** (0.213 g, 0.430 mmol), **S7** (0.457 g, 0.473 mmol), $PdCl_2$(dppf) (6.3 mg, 0.0086 mmol), and K_3PO_4 (0.183 g, 0.860 mmol) in degassed DME/H_2O (4:1, 12.5 mL) was stirred at 80 °C for 3 h. The reaction mixture was diluted with water and extracted with ethyl acetate (20 mL x 3). The organic layer was washed with brine, dried over Na_2SO_4, filtered, and concentrated. The residue was passed through silica gel column chromatography (eluent: *n*-hexane/EtOAc = 10:1) to give crude **S14**. This crude **S14** was used for the next reaction without further purification.

To a solution of the crude **S14** in THF (6 mL) was added TBAF (0.75 mL, 0.75 mmol, 1.0 mol/L in THF) at 0 °C. After stirring at room temperature for 30 min, the reaction mixture was diluted with water and extracted with ethyl acetate (10 mL x 3). The organic layer was washed with brine, dried over Na_2SO_4, filtered, and concentrated. The residue was purified by silica gel column chromatography (eluent: *n*-hexane/DCM = 3:1) to give **1d** (0.0961 g, 0.265 mmol, 62% yield).

Red oil; ^{1}H NMR ($CDCl_3$, 400 MHz) δ 8.51–8.48 (m, 1H), 7.89 (m, 2H), 7.74 (dd, J = 1.6, 1.5 Hz, 1H), 7.73 (dd, J = 1.9, 1.6 Hz, 1H), 7.66–7.52 (m, 5H), 7.45–7.40 (m, 2H), 7.36–7.31 (m, 1H), 3.54 (s, 1H), 3.13 (s, 1H); ^{13}C NMR ($CDCl_3$, 100 MHz) δ 142.9, 142.1, 141.9, 141.5, 134.0, 134.0, 133.4, 132.3, 129.5, 129.2, 129.2, 129.0, 128.8, 128.4, 128.2, 127.6, 127.6, 127.1, 126.7, 126.7, 120.5, 117.5, 86.2, 82.8, 80.9, 80.7; HRMS (ESI) calcd for $C_{26}H_{16}Cl$ [M+H]$^+$ 363.0935 found 363.0924.

((1-Bromonaphthalen-2-yl)ethynyl)triisopropylsilane (S16)

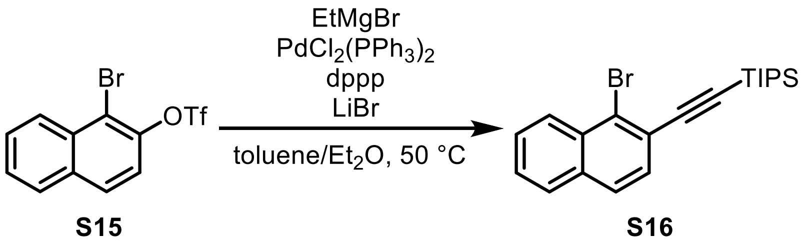

A solution of EtMgBr (3.83 mL, 11.5 mmol, 3.0 mol/L in Et_2O) and triisopropylsilylacetylene (2.19 g, 12.0 mmol) in toluene/Et_2O (1:1, 30 mL) was stirred at 50 °C for 1 h. To the resulting mixture were added $PdCl_2(PPh_3)_2$ (0.140 g, 0.200 mmol), dppp (0.0825 g, 0.200 mmol), LiBr (0.870 g, 10.0 mmol), and **S15**[3] (3.55 g, 10.0 mmol). After stirring at room temperature for 8 h, the reaction mixture was diluted with water and extracted with Et_2O (100 mL x 3). The organic layer was washed with brine, dried over Na_2SO_4, filtered, and concentrated. The residue was purified by silica gel column chromatography (eluent: *n*-hexane) to give **S16** (2.85 g, 7.36 mmol, 74% yield).

White solid; mp 54.7–55.0 °C; ^1H NMR ($CDCl_3$, 400 MHz) δ 8.29 (d, J = 8.7 Hz, 1H), 7.78 (d, J = 8.1 Hz, 1H), 7.71 (d, J = 8.4 Hz, 1H), 7.62–7.57 (m, 1H), 7.54–7.49 (m, 2H), 1.19–1.18 (m, 21H); ^{13}C NMR ($CDCl_3$, 100 MHz) δ 133.7, 132.2, 129.5, 128.1, 127.9, 127.8, 127.3, 127.1, 126.7, 123.7, 106.0, 97.2, 18.7, 11.4; HRMS (APCI) calcd for $C_{21}H_{28}BrSi$ $[M+H]^+$ 387.1138 found 387.1140.

(2-((Triisopropylsilyl)ethynyl)naphthalen-1-yl)boronic acid (S17)

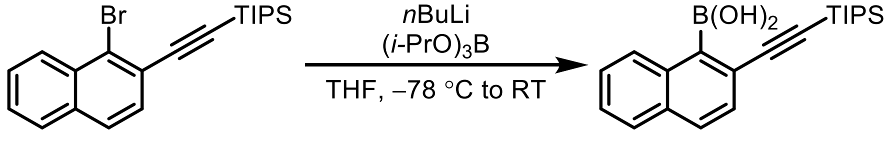

S16 **S17**

A 1.6 M solution of *n*-BuLi (4.62 mL, 7.39 mmol, 1.6 mol/L in THF) was added dropwise to a solution of **S16** (2.36 g, 6.08 mmol) in THF (30 mL) at –78 °C. After stirring at –78 °C for 1 h, $B(Oi\text{-}Pr)_3$ (1.37 g, 7.30 mmol) was added at –78 °C. The reaction mixture was stirred for 21 h while the temperature was raised to room temperature. The reaction was quenched with 1 M aqueous HCl solution (50 mL). After stirring at room temperature for 2 h, the solution was extracted with ethyl acetate (100 mL x 3), dried over Na_2SO_4, filtered, and concentrated. The residue was purified by silica gel column chromatography (eluent: *n*-hexane/EtOAc = 10:1) to give **S17** (1.15 g, 3.26 mmol, 54% yield).

Yellow solid; mp 69.3–69.9 °C; ^1H NMR ($CDCl_3$, 400 MHz) δ 8.48 (d, J = 8.4 Hz, 1H), 7.83–7.79 (m, 2H), 7.55–7.47 (m, 3H), 5.57–5.54 (m, 2H), 1.21–1.15 (m, 21H); ^{13}C NMR ($CDCl_3$, 100 MHz) δ 135.6, 132.8, 130.0, 129.2, 128.7, 128.3, 127.0, 126.6, 125.2, 108.3, 95.5, 18.7, 11.3; HRMS (ESI) calcd for $C_{21}H_{29}BO_2SiNa$ $[M+Na]^+$ 375.1922 found 375.1929.

2-Ethynyl-1-(2'-ethynyl-5-methyl-[1,1'-biphenyl]-3-yl)naphthalene (1e)

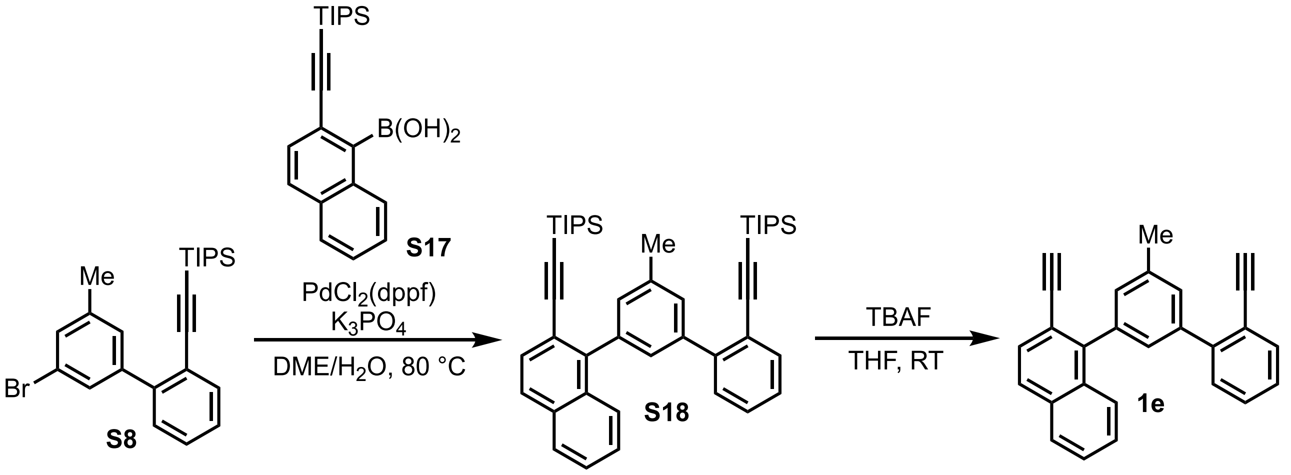

A solution of **S8** (0.232 g, 0.543 mmol), **S17** (0.191 g, 0.543 mmol), $PdCl_2$(dppf) (7.9 mg, 0.011 mmol), and K_3PO_4 (0.231 g, 1.09 mmol) in degassed DME/H_2O (4:1, 12.5 mL) was stirred at 80 °C

for 13 h. The reaction mixture was diluted with water and extracted with ethyl acetate (20 mL x 3). The organic layer was washed with brine, dried over Na_2SO_4, filtered, and concentrated. The residue was passed through silica gel column chromatography (eluent: *n*-hexane/DCM = 4:1) to give crude **S18**. This crude **S18** was used for the next reaction without further purification.

To a solution of the crude **S18** in THF (10 mL) was added TBAF (1.00 mL, 1.00 mmol, 1.0 mol/L in THF) at 0 °C. After stirring at room temperature for 30 min, the reaction mixture was diluted with water and extracted with ethyl acetate (20 mL x 3). The organic layer was washed with brine, dried over Na_2SO_4, filtered, and concentrated. The residue was purified by silica gel column chromatography (eluent: *n*-hexane/DCM = 4:1) to give **1e** (0.131 g, 0.382 mmol, 70% yield).

Yellow oil; ^{1}H NMR ($CDCl_3$, 400 MHz) δ 7.84 (d, J = 8.1 Hz, 1H), 7.79 (d, J = 8.5 Hz, 1H), 7.70 (d, J = 8.5 Hz, 1H), 7.63 (d, J = 8.5 Hz, 1H), 7.60 (dd, J = 7.7, 1.1 Hz, 1H), 7.50–7.46 (m, 4H), 7.41–7.36 (m, 2H), 7.27 (ddd, J = 7.5, 7.5, 1.4 Hz, 1H), 7.25–7.24 (m, 1H), 3.05 (s, 1H), 3.01 (s, 1H), 2.48 (s, 3H); ^{13}C NMR ($CDCl_3$, 100 MHz) δ 144.4, 143.8, 139.9, 138.1, 137.3, 133.9, 133.3, 132.3, 130.2, 129.7, 129.2, 129.1, 128.9, 128.5, 127.9, 127.4, 127.2, 127.0, 126.6, 126.4, 120.5, 118.9, 83.7, 83.3, 80.7, 80.3, 21.5; HRMS (ESI) calcd for $C_{27}H_{19}$ $[M+H]^+$ 343.1481 found 343.1480.

((1-(3-Bromo-5-chlorophenyl)naphthalen-2-yl)ethynyl)triisopropylsilane (S19)

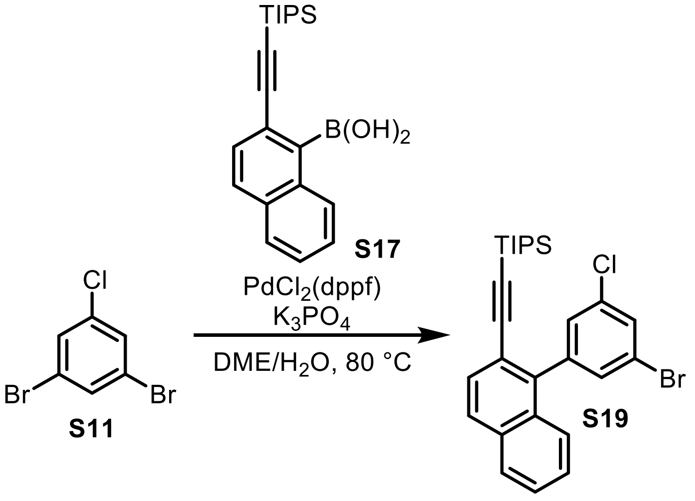

A solution of **S11** (0.811 g, 3.00 mmol), **S17** (0.352 g, 1.00 mmol), $PdCl_2$(dppf) (7.3 mg, 0.010 mmol), and K_3PO_4 (0.425 g, 2.00 mmol) in degassed DME/H_2O (4:1, 10 mL) was stirred at 80 °C for 14 h. The reaction mixture was diluted with water and extracted with ethyl acetate (20 mL x 3). The organic layer was washed with brine, dried over Na_2SO_4, filtered, and concentrated. The residue was passed through silica gel column chromatography (eluent: *n*-hexane) to give **S19** (0.266 g, 0.534 mmol, 53% yield).

Yellow solid; mp 71.0–72.3 °C; ^{1}H NMR ($CDCl_3$, 400 MHz) δ 7.85 (d, J = 8.0 Hz, 1H), 7.81 (d, J = 8.4 Hz, 1H), 7.60 (d, J = 8.5 Hz, 1H), 7.58 (dd, J = 1.9, 1.9 Hz, 1H), 7.52–7.41 (m, 4H), 7.35 (dd, J = 1.8, 1.4 Hz, 1H), 1.00–0.99 (m, 21H); ^{13}C NMR ($CDCl_3$, 100 MHz) δ 142.4, 140.1, 134.9, 132.9, 131.7, 131.6, 130.4, 129.4, 128.8, 128.2, 128.1, 127.0, 126.6, 126.0, 122.6, 120.6, 105.8, 96.2, 18.5, 11.1; HRMS (APCI) calcd for $C_{27}H_{31}BrClSi$ $[M+H]^+$ 497.1061 found 497.1075.

((1-(3-Chloro-5-(4,4,5,5-tetramethyl-1,3,2-dioxaborolan-2-yl)phenyl)naphthalen-2-yl)-ethynyl)triisopropylsilane (S20)

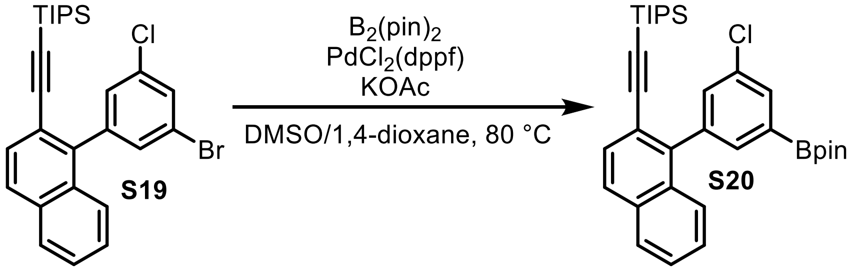

A solution of **S19** (0.224 g, 0.450 mmol), B_2pin_2 (0.137 g, 0.540 mmol), $PdCl_2(dppf)$ (6.6 mg, 0.0090 mmol), and KOAc (0.133 g, 1.35 mmol) in degassed DMSO/1,4-dioxane (1:1, 10 mL) was stirred at 80 °C for 17 h. The reaction mixture was diluted with water and extracted with ethyl acetate (20 mL x 3). The organic layer was washed with brine, dried over Na_2SO_4, filtered, and concentrated. The residue was passed through silica gel column chromatography (eluent: *n*-hexane/EtOAc = 10:1) to give **S20** (0.167 g, 0.306 mmol, 68% yield).

White solid; mp 81.2–81.7 °C; ^{1}H NMR ($CDCl_3$, 400 MHz) δ 7.84–7.82 (m, 2H), 7.78 (d, J = 8.5 Hz, 1H), 7.69 (dd, J = 1.5, 1.0 Hz, 1H), 7.59 (d, J = 8.5 Hz, 1H), 7.48–7.45 (m, 3H), 7.41–7.37 (m, 1H), 1.33 (s, 12H), 0.96–0.94 (m, 21H); ^{13}C NMR ($CDCl_3$, 100 MHz) δ 141.9, 140.5, 134.8, 133.9, 133.5, 132.9, 132.9, 132.0, 130.9, 128.8, 127.9, 127.6, 126.6, 126.6, 126.4, 120.5, 106.3, 95.3, 84.1, 24.9, 18.5, 11.1; HRMS (ESI) calcd for $C_{33}H_{42}BClO_2SiNa$ $[M+Na]^+$ 567.2628 found 567.2622.

1-(3-Chloro-5-(1-ethynylnaphthalen-2-yl)phenyl)-2-ethynylnaphthalene (1f)

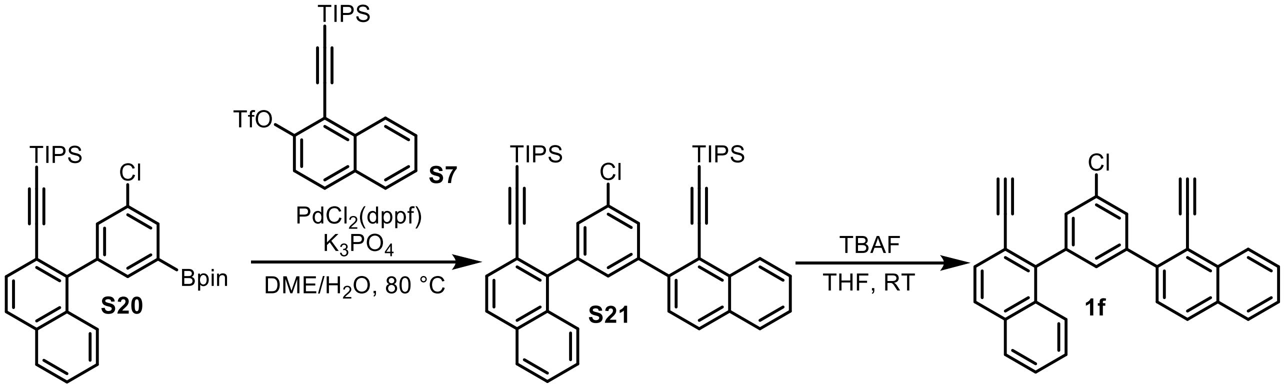

A solution of **S20** (0.136 g, 0.250 mmol), **S7** (0.126 g, 0.275 mmol), $PdCl_2(dppf)$ (7.3 mg, 0.010 mmol), and K_3PO_4 (0.106 g, 0.500 mmol) in degassed DME/H_2O (4:1, 12.5 mL) was stirred at 80 °C for 3 h. The reaction mixture was diluted with water and extracted with ethyl acetate (30 mL x 3). The organic layer was washed with brine, dried over Na_2SO_4, filtered, and concentrated. The residue was passed through silica gel column chromatography (eluent: *n*-hexane/EtOAc = 10:1) to give crude **S21**. This crude **S21** was used for the next reaction without further purification.

To a solution of the crude **S21** in THF (6 mL) was added TBAF (0.44 mL, 0.44 mmol, 1.0 mol/L in THF) at 0 °C. After being stirred at room temperature for 30 min, the reaction mixture was diluted with water, and extracted with ethyl acetate (20 mL x 3). The organic layer was washed with brine, dried over Na_2SO_4, filtered, and concentrated. The residue was purified by silica gel column chromatography (eluent: *n*-hexane/DCM = 3:1) to give **1f** (0.0736 g, 0.178 mmol, 71% yield).

Red oil; ^{1}H NMR ($CDCl_3$, 400 MHz) δ 8.47 (dd, J = 8.5, 0.8 Hz, 1H), 7.90–7.81 (m, 4H), 7.77 (dd, J = 1.9, 1.8 Hz, 1H), 7.71 (dd, J = 8.5, 0.6 Hz, 1H), 7.65–7.59 (m, 3H), 7.56 (d, J = 8.6 Hz, 1H), 7.54–7.42 (m, 4H), 3.52 (s, 1H), 3.08 (s, 1H); ^{13}C NMR ($CDCl_3$, 100 MHz) δ 142.2, 142.0, 141.7,

139.9, 134.0, 133.6, 133.3, 132.3, 131.9, 130.3, 129.5, 129.2, 129.0, 128.9, 128.2, 128.1, 128.0, 127.5, 127.1, 126.8, 126.8, 126.7, 126.7, 126.7, 119.1, 117.6, 86.3, 83.2, 81.4, 80.7; HRMS (ESI) calcd for $C_{30}H_{17}ClNa$ $[M+Na]^+$ 435.0911 found 435.0915.

2-2. Synthesis of *o,m,o,p*-Tetraphenylenes

o,m,o,p-Tetraphenylene 3a and *o,m,o,m*-tetraphenylene 4a

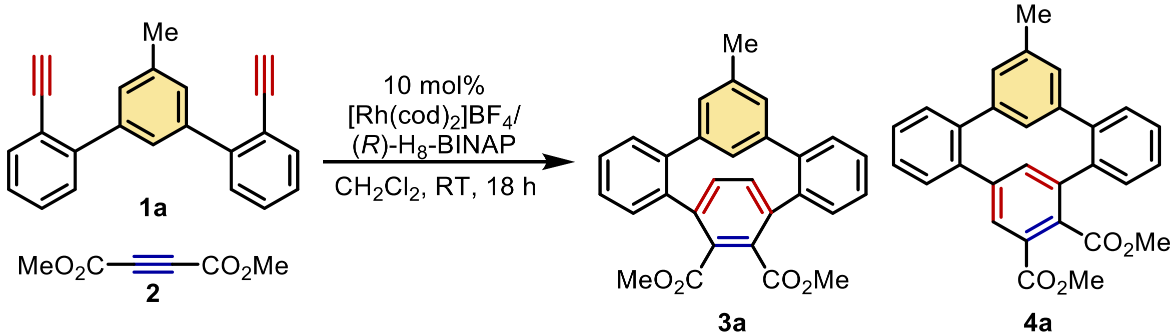

(R)-H$_8$-BINAP (6.3 mg, 0.010 mmol) and [Rh(cod)$_2$]BF$_4$ (4.1 mg, 0.010 mmol) were dissolved in CH_2Cl_2 (1.0 mL), and the mixture was stirred at room temperature for 10 min. H_2 was introduced to the resulting solution in a Schlenk tube. After stirring at room temperature for 30 min, the resulting mixture was concentrated under reduced pressure. The residue was dissolved in CH_2Cl_2 (1.0 mL) and added to a solution of **1a** (29.3 mg, 0.100 mmol) and **2** (15.6 mg, 0.110 mmol) in CH_2Cl_2 (3.0 mL). The mixture was stirred at room temperature for 18 h. The resulting mixture was concentrated and purified by silica gel PTLC (eluent: *n*-hexane/EtOAc = 3:1) to give **3a** and **4a** (17.8 mg, 0.0410 mmol, 41% yield, **3a**/**4a** = 84:16).

Pale yellow solid; mp 107.8–110.5 °C (**3a**/**4a** = 84:16); ^{1}H NMR (CDCl$_3$, 400 MHz) δ 7.71–7.64 (m, 2H, **3a**; 6H, **4a**), 7.54–7.39 (m, 8H, **3a**; 4H, **4a**), 7.21 (s, 1H, **4a**), 6.90 (s, 2H, **3a**), 6.57 (s, 1H, **4a**), 6.49 (s, 1H, **4a**), 5.15 (s, 1H, **3a**), 3.77 (s, 3H, **4a**), 3.58 (s, 3H, **4a**), 3.44 (broad, 6H, **4a**), 2.34 (s, 3H, **3a**), 2.09 (s, 3H, **4a**); ^{13}C NMR (CDCl$_3$, 100 MHz) δ 168.5, 165.9, 144.4, 144.1, 143.9, 143.3, 142.6, 142.1, 140.0, 137.9, 137.4, 137.2, 136.8, 136.3, 136.0, 134.5, 134.1, 134.0, 130.0, 129.2, 128.5, 128.5, 128.3, 128.1, 127.9, 127.4, 127.4, 126.8, 126.5, 126.5, 126.4, 125.5, 124.1, 123.9, 52.3, 52.0, 51.9, 21.5, 21.1; HRMS (ESI) calcd for $C_{29}H_{22}O_4Na$ [M+Na]$^+$ 457.1410 found 457.1410.

o,m,o,p-Tetraphenylene *cis*-3b and *o,m,o,m*-tetraphenylene 4b

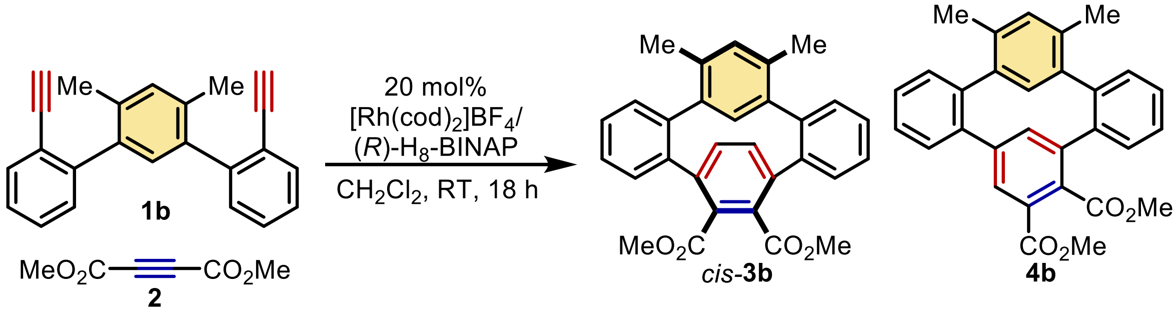

(R)-H$_8$-BINAP (12.6 mg, 0.0200 mmol) and [Rh(cod)$_2$]BF$_4$ (8.1 mg, 0.020 mmol) were dissolved in CH_2Cl_2 (1.0 mL), and the mixture was stirred at room temperature for 10 min. H_2 was introduced to the resulting solution in a Schlenk tube. After stirring at room temperature for 30 min, the resulting mixture was concentrated under reduced pressure. The residue was dissolved in CH_2Cl_2 (1.0 mL) and added to a solution of **1b** (30.6 mg, 0.100 mmol) and **2** (15.6 mg, 0.110 mmol) in CH_2Cl_2 (3.0 mL). The mixture was stirred at room temperature for 18 h. The resulting mixture was concentrated and purified by silica gel PTLC (eluent: *n*-hexane/EtOAc = 3:1) to give *cis*-**3b** and **4b** (12.5 mg, 0.0279 mmol, 28% yield, *cis*-**3b**/**4b** = 78:22). Subsequent recrystallization from hexane/EtOAc gave *cis*-**3b** (7.1 mg, 0.0158 mmol, 16% yield).

Pale yellow solid; mp 110.1–112.1 °C (*cis*-**3b**); ^{1}H NMR (CDCl$_3$, 400 MHz) δ 7.73–7.71 (m, 2H, **3b**), 7.69–7.67 (m, 1H, **4b**), 7.65–7.63 (m, 1H, **4b**), 7.47–7.43 (m, 2H, **3b**; 1H, **4b**), 7.41 (s, 2H, **3b**), 7.38–7.36 (m, 4H, **3b**; 1H, **4b**), 7.20 (s, 1H, **4b**), 6.97 (s, 1H, **3b**), 6.54 (s, 1H, **4b**), 5.12 (s, 1H, **3b**), 3.78 (s, 3H, **4b**), 3.62 (s, 3H, **4b**), 3.49 (s, 6H, **3b**), 2.15 (s, 6H, **3b**), 1.86 (s, 3H, **4b**), 1.75 (s, 3H,

4b); ^{13}C NMR ($CDCl_3$, 100 MHz) δ 168.5, 166.0, 165.9, 143.2, 143.0, 142.8, 142.6, 142.3, 141.4, 140.9, 140.0, 139.2, 138.0, 136.8, 136.3, 135.6, 135.5, 135.1, 134.2, 133.5, 133.3, 133.0, 132.1, 130.6, 129.8, 128.3, 128.2, 128.0, 127.7, 127.6, 127.5, 127.2, 126.8, 126.7, 125.2, 124.5, 52.3, 51.9, 51.9, 20.1, 18.9, 18.5; HRMS (ESI) calcd for $C_{30}H_{24}O_4Na$ $[M+Na]^+$ 471.1567 found 471.1603.

(+)-*o*,*m*,*o*,*p*-Tetraphenylene (+)-3c

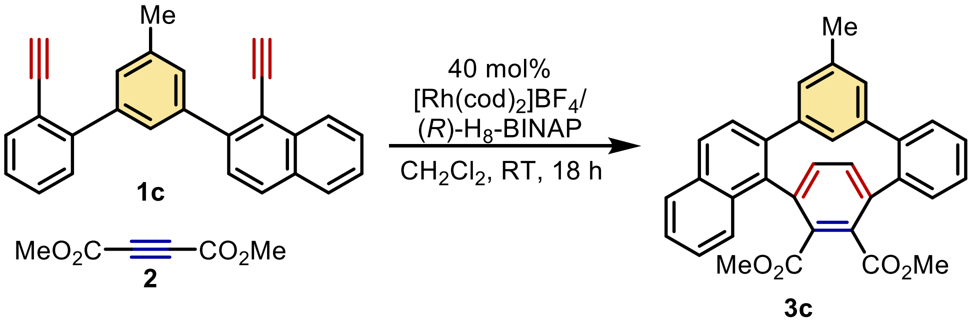

(R)-H_8-BINAP (18.9 mg, 0.0300 mmol) and $[Rh(cod)_2]BF_4$ (12.2 mg, 0.0300 mmol) were dissolved in CH_2Cl_2 (1.0 mL), and the mixture was stirred at room temperature for 10 min. H_2 was introduced to the resulting solution in a Schlenk tube. After stirring at room temperature for 30 min, the resulting mixture was concentrated under reduced pressure. The residue was dissolved in CH_2Cl_2 (1.0 mL) and added to a solution of **1c** (25.7 mg, 0.0750 mmol) and **2** (11.7 mg, 0.0825 mmol) in CH_2Cl_2 (5.0 mL). The mixture was stirred at room temperature for 18 h. The resulting mixture was concentrated and purified by silica gel PTLC (eluent: *n*-hexane/EtOAc = 3:1) to give **3c** (13.2 mg, 0.0272 mmol, 36% yield, 98% ee).

Pale yellow solid; mp 120.5–121.3 °C; $[\alpha]^{25}_D$ +138.4° (c 4.78 mg/cm^3, $CHCl_3$, 96% ee), +119.6° (c 4.78 mg/cm^3, CH_2Cl_2, 96% ee), +323.5° (c 4.78 mg/cm^3, acetone, 96% ee); ^{1}H NMR ($CDCl_3$, 400 MHz) δ 8.34 (s, 1H), 7.94 (m, 2H), 7.74–7.69 (m, 3H), 7.63–7.55 (m, 4H), 7.50–7.42 (m, 2H), 6.95 (s, 2H), 5.28 (s, 1H), 3.47 (s, 3H), 3.23 (s, 3H), 2.37 (s, 3H); ^{13}C NMR ($CDCl_3$, 100 MHz) δ 166.0, 165.9, 144.1, 142.0, 140.7, 140.0, 137.1, 136.8, 135.6, 134.5, 134.5, 132.8, 130.4, 130.1, 130.0, 128.4, 128.4, 128.2, 128.2, 127.9, 127.6, 127.1, 127.1, 127.0, 126.6, 126.6, 126.1, 125.4, 124.2, 124.2, 52.0, 51.8, 21.6; HRMS (ESI) calcd for $C_{33}H_{24}O_4Na$ $[M+Na]^+$ 507.1567 found 507.1613; CHIRALPAK IF-3, *n*-hexane/2-propanol = 93:7, 1.0 mL min^{-1}, retention times: 11.18 min (major isomer) and 13.42 min (minor isomer).

o,*m*,*o*,*p*-Tetraphenylene 3d and *o*,*m*,*o*,*m*-tetraphenylene 4d

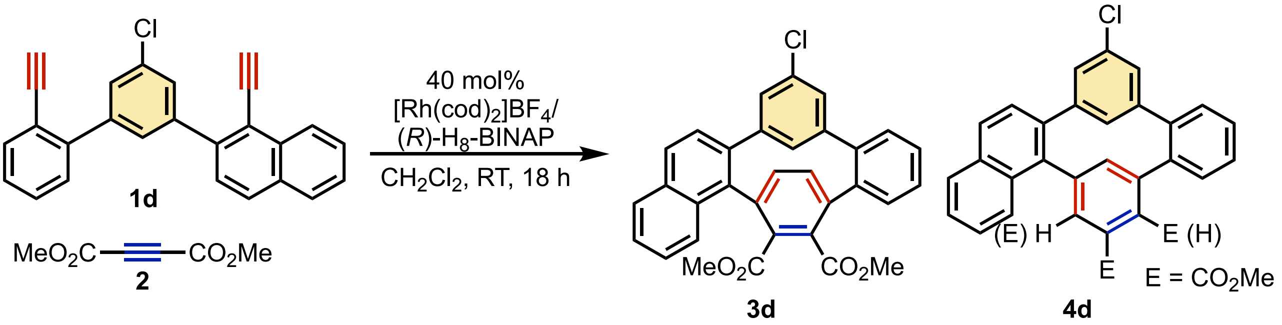

(R)-H_8-BINAP (20.3 mg, 0.0322 mmol) and $[Rh(cod)_2]BF_4$ (13.1 mg, 0.0322 mmol) were dissolved in CH_2Cl_2 (1.0 mL), and the mixture was stirred at room temperature for 10 min. H_2 was introduced to the resulting solution in a Schlenk tube. After stirring at room temperature for 30 min, the resulting mixture was concentrated under reduced pressure. The residue was dissolved in CH_2Cl_2 (1.0 mL) and added to a solution of **1d** (29.2 mg, 0.0804 mmol) and **2** (12.6 mg, 0.0884 mmol) in CH_2Cl_2 (5.4 mL). The mixture was stirred at room temperature for 18 h. The resulting mixture was concentrated and purified by silica gel PTLC (eluent: *n*-hexane/EtOAc = 3:1) to give **3d** and **4d** (13.8 mg, 0.0273 mmol, 34% yield, **3d**/**4d** = 90:10, 97% ee).

Pale yellow solid; mp 100.2–103.1 °C (**3d**/**4d** = 90:10); ^{1}H NMR ($CDCl_3$, 400 MHz) δ 8.34 (s, 1H, **3d**; 1H, **4d**), 7.96–7.93 (m, 2H, **3d**; 1H, **4d**), 7.75–7.44 (m, 9H, **3d**; 9H, **4d**), 7.34 (s, 1H, **4d**), 7.21 (s, 2H, **4d**), 7.15 (s, 2H, **3d**), 6.43 (s, 1H, **4d**), 5.30 (s, 1H, **3d**), 3.49 (s, 3H, **3d**), 3.40 (s, 3H, **4d**), 3.28 (s, 3H, **3d**, 3H, **4d**); ^{13}C NMR ($CDCl_3$, 100 MHz) δ 167.0, 165.9, 143.7, 142.7, 142.0, 141.5, 139.7, 139.2, 138.2, 137.4, 137.1, 135.5, 134.8, 134.4, 133.7, 133.1, 132.8, 132.6, 132.3, 130.3, 130.3, 129.7, 129.1, 129.0, 128.9, 128.9, 128.6, 128.4, 128.0, 127.8, 127.1, 127.0, 126.9, 126.6, 126.5, 125.3, 124.5, 52.2, 52.1, 52.0; HRMS (ESI) calcd for $C_{32}H_{21}ClO_4Na$ $[M+Na]^+$ 527.1021 found 527.1065; CHIRALPAK IF-3, *n*-hexane/2-propanol = 93:7, 1.0 mL min^{-1}, retention times: 11.75 min (minor isomer) and 13.59 min (major isomer).

***o*,*m*,*o*,*p*-Tetraphenylenes *cis*-3e and *trans*-3e, and *o*,*m*,*o*,*m*-tetraphenylene 4e**

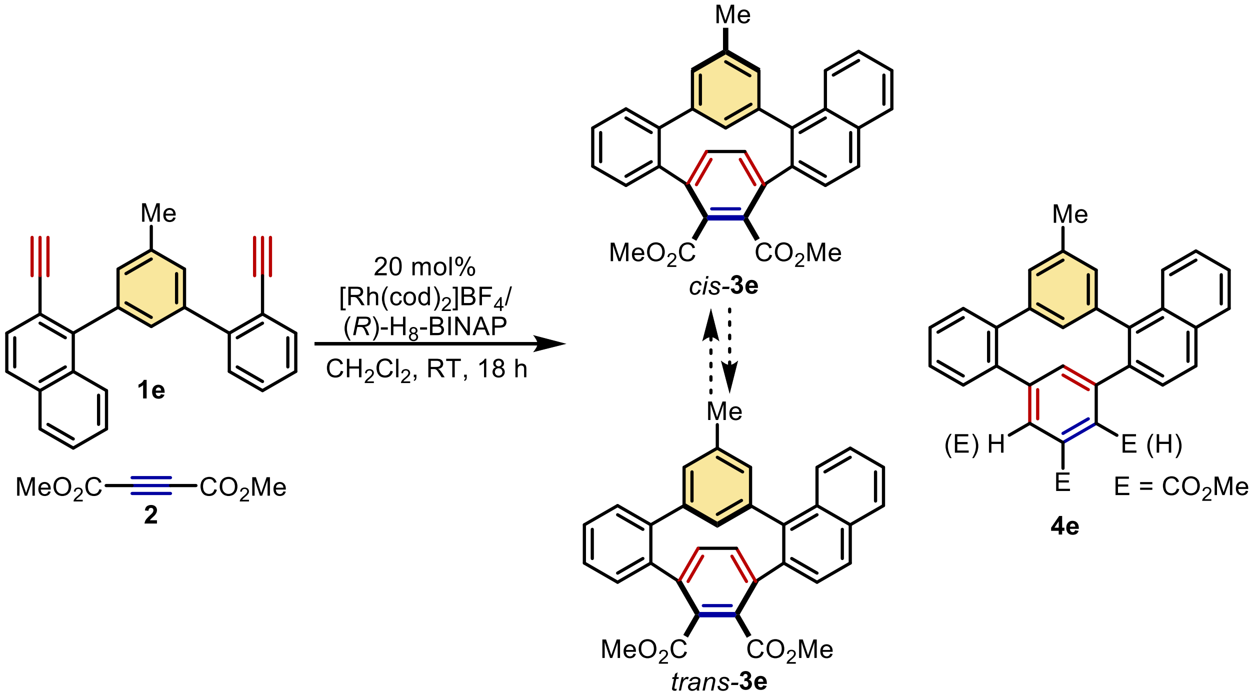

(*R*)-H$_8$-BINAP (18.2 mg, 0.0288 mmol) and $[Rh(cod)_2]BF_4$ (11.7 mg, 0.0288 mmol) were dissolved in CH_2Cl_2 (1.0 mL), and the mixture was stirred at room temperature for 10 min. H_2 was introduced to the resulting solution in a Schlenk tube. After stirring at room temperature for 30 min, the resulting mixture was concentrated under reduced pressure. The residue was dissolved in CH_2Cl_2 (1.0 mL) and added to a solution of **1e** (24.7 mg, 0.0721 mmol) and **2** (11.3 mg, 0.0793 mmol) in CH_2Cl_2 (4.8 mL). The mixture was stirred at room temperature for 18 h. The resulting mixture was concentrated and purified by silica gel PTLC (eluent: *n*-hexane/EtOAc = 10:1) to give **3e** and **4e** (16.6 mg, 0.0343 mmol, 48% yield, *cis*-**3e**/*trans*-**3e**/**4e** = 76:19:5, *cis*-**3e**: 40% ee).

Pale yellow solid; mp 98.2–102.7 °C (*cis*-**3e**/*trans*-**3e**/**4e** = 76:19:5); ^{1}H NMR ($CDCl_3$, 400 MHz) δ 8.14–8.11 (m, 1H, **4e**), 8.10–8.07 (m, 1H, *cis*-**3e**; 1H, *trans*-**3e**), 7.98 (d, J = 8.3 Hz, 1H, *cis*-**3e**), 7.94–7.92 (m, 1H, *cis*-**3e**; 2H, **4e**), 7.91–7.89 (m, 2H, *trans*-**3e**), 7.87 (d, J = 8.3 Hz, 1H, *cis*-**3e**), 7.83–7.79 (m, 1H, *trans*-**3e**, 1H, **4e**), 7.71–7.65 (m, 1H, *cis*-**3e**; 2H, *trans*-**3e**), 7.60–7.58 (m, 1H, *trans*-**3e**; 1H, **4e**), 7.56 (s, 1H, *trans*-**3e**), 7.55–7.42 (m, 7H, *cis*-**3e**; 2H, *trans*-**3e**; 8H, **4e**), 6.94–6.94 (m, 2H, *cis*-**3e**), 6.88 (d, J = 0.6 Hz, 1H, *trans*-**3e**), 6.83 (d, J = 0.6 Hz, 1H, *trans*-**3e**), 6.73 (d, J = 8.1 Hz, 1H, *trans*-**3e**), 6.66–6.64 (m, 1H, *trans*-**3e**, 1H, **4e**), 6.53–6.52 (m, 1H, **4e**), 5.54 (s, 1H, *trans*-**3e**), 5.44 (s, 1H, *cis*-**3e**), 3.79 (s, 3H, **4e**), 3.63 (s, 3H, *trans*-**3e**), 3.57 (s, 3H, **4e**), 3.56 (s, 3H, *trans*-**3e**), 3.47 (s, 3H, *cis*-**3e**), 3.41 (s, 3H, *cis*-**3e**), 2.34 (s, 3H, *cis*-**3e**), 2.32 (s, 3H, *trans*-**3e**), 2.10 (s, 3H, **4e**); ^{13}C NMR ($CDCl_3$, 100 MHz) δ 167.5, 167.3, 166.0, 165.8, 144.8, 144.2, 143.6, 143.0, 142.1, 141.7, 141.4, 141.0, 140.7, 140.3, 139.9, 139.1, 138.1, 137.0, 136.8, 136.3, 136.2, 135.5, 135.3, 135.2, 134.4, 133.7, 133.5, 133.4, 132.9, 132.4, 132.1, 131.9, 131.8, 131.6, 131.3, 130.7, 128.4, 128.2, 128.1, 128.0, 127.6, 127.2, 126.8, 126.7, 126.6, 126.3, 126.1, 125.9, 125.8, 125.4, 124.9 124.0, 52.2, 51.9, 21.5, 21.4; HRMS (ESI) calcd for $C_{33}H_{24}O_4Na$ $[M+Na]^+$ 507.1567 found

507.1581; HRMS (ESI) calcd for $C_{32}H_{21}ClO_4Na$ [M+Na]$^+$ 527.1021 found 527.1065; CHIRALPAK IF-3, *n*-hexane/2-propanol = 93:7, 1.0 mL min^{-1}, retention times: 9.63 min (major isomer) and 10.31 min (minor isomer).

***o,m,o,p*-Tetraphenylenes *cis*-3f and *trans*-3f, and *o,m,o,m*-tetraphenylene 4f**

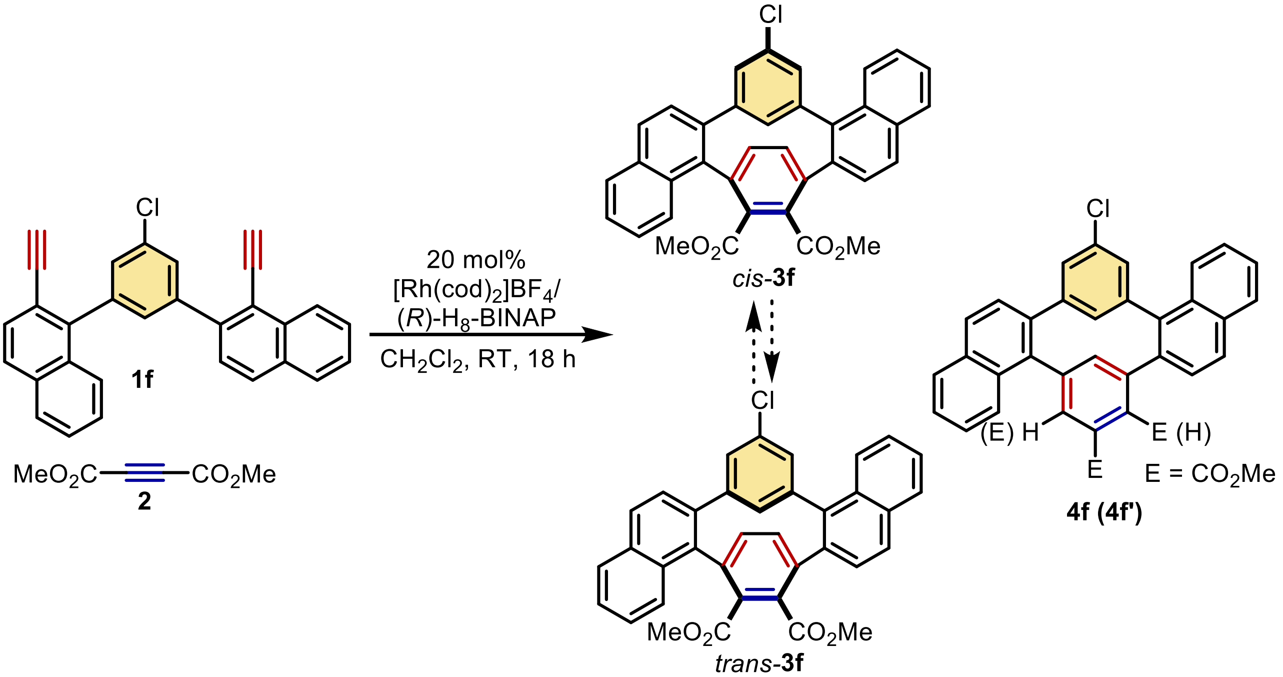

【Isolation of *cis*-**3f**/*trans*-**3f**/**4f**】

(*R*)-H_8-BINAP (15.6 mg, 0.0248 mmol) and $[Rh(cod)_2]BF_4$ (10.1 mg, 0.0248 mmol) were dissolved in CH_2Cl_2 (1.0 mL), and the mixture was stirred at room temperature for 10 min. H_2 was introduced to the resulting solution in a Schlenk tube. After stirring at room temperature for 30 min, the resulting mixture was concentrated under reduced pressure. The residue was dissolved in CH_2Cl_2 (1.0 mL) and added to a solution of **1f** (25.6 mg, 0.0620 mmol) and **2** (9.7 mg, 0.068 mmol) in CH_2Cl_2 (4.0 mL). The mixture was stirred at room temperature for 18 h. The resulting mixture was concentrated and purified by silica gel PTLC (eluent: *n*-hexane/EtOAc = 3:1) to give **3f** and **4f** (11.9 mg, 0.0214 mmol, 35% yield, *cis*-**3f**/*trans*-**3f**/**4f** = 75:13:12, *cis*-**3f**: 93% ee).

Pale yellow solid; ^{1}H NMR ($CDCl_3$, 400 MHz) δ 8.32 (d, J = 8.2 Hz, 1H, *cis*-**3f**), 8.11–7.48 (m, 13H, *cis*-**3f**, 12H, *trans*-**3f**; 16H, **4f**, 14H, **4f'**), 7.21 (s, 1H, *cis*-**3f**), 7.17 (s, 1H, *cis*-**3f**), 7.10–7.09 (m, 2H, *trans*-**3f**), 7.01–6.99 (m, 2H, **4f'**), 6.91 (d, J = 8.2 Hz, 1H, *trans*-**3f**), 6.86–6.85 (m, 1H, **4f'**), 6.79 (d, J = 8.0 Hz, 1H, *trans*-**3f**), 5.75 (s, 1H, *trans*-**3f**), 5.63 (s, 1H, *cis*-**3f**), 3.57–3.56 (m, 3H, *trans*-**3f**, 3H, **4f'**), 3.49 (s, 3H, *cis*-**3f**), 3.38 (s, 3H, *cis*-**3f**), 3.20 (s, 3H, **4f'**), 3.15 (s, 3H, *trans*-**3f**), 2.95 (s, 3H, **4f**), 2.88 (s, 3H, **4f**); ^{13}C NMR ($CDCl_3$, 100 MHz) δ 165.8, 165.8, 144.0, 143.9, 142.3, 141.4, 140.5, 139.8, 139.6, 138.7, 138.0, 137.3, 137.1, 136.5, 135.8, 135.4, 134.9, 134.2, 133.9, 133.7, 133.5, 133.2, 133.1, 132.6, 132.5, 131.6, 131.4, 131.1, 130.8, 130.7, 130.4, 130.2, 130.0, 129.7, 128.7, 128.6, 128.5, 128.4, 128.2, 128.0, 127.9, 127.7, 127.4, 127.3, 127.1, 127.0, 126.8, 126.7, 126.5, 126.4, 126.3, 126.2, 126.1, 125.6, 123.8, 52.2, 52.1, 36.5; HRMS (ESI) calcd for $C_{36}H_{23}ClO_4Na$ [M+Na]$^+$ 577.1177 found 577.1202; CHIRALPAK IF-3, *n*-hexane/2-propanol = 93:7, 1.0 mL min^{-1}, retention times: 11.52 min (minor isomer) and 13.97 min (major isomer).

【Isolation of *cis*-**3f**/*trans*-**3f**】

(*R*)-H_8-BINAP (15.7 mg, 0.0249 mmol) and $[Rh(cod)_2]BF_4$ (10.1 mg, 0.0248 mmol) were dissolved in CH_2Cl_2 (1.0 mL), and the mixture was stirred at room temperature for 10 min. H_2 was introduced to the resulting solution in a Schlenk tube. After stirring at room temperature for 30 min, the resulting mixture was concentrated under reduced pressure. The residue was dissolved in CH_2Cl_2 (1.0 mL) and added to a solution of **1f** (25.6 mg, 0.0620 mmol) and **2** (9.9 mg, 0.070 mmol)

in CH_2Cl_2 (4.0 mL). The mixture was stirred at room temperature for 18 h. The resulting mixture was concentrated and purified by repeated silica gel PTLC (1st PTLC eluent: *n*-hexane/EtOAc = 3:1, 2nd PTLC eluent: *n*-hexane/ CH_2Cl_2 = 1:1, 3rd PTLC eluent: *n*-hexane/EtOAc = 3:1) to give **3f** (4.2 mg, 0.0076 mmol, 12% yield, *cis*-**3f**/*trans*-**3f** = 89:11, *cis*-**3f**: 93% ee).

White solid; mp 112.5–113.9 °C (*cis*-**3f**/*trans*-**3f** = 89:11); ^{1}H NMR ($CDCl_3$, 400 MHz) δ 8.32 (d, J = 8.2 Hz, 1H, *cis*-**3f**), 8.12–8.10 (m, 1H, *trans*-**3f**), 8.06–8.02 (m, 2H, *cis*-**3f**; 1H, *trans*-**3f**), 7.98–7.93 (m, 3H, *cis*-**3f**; 5H, *trans*-**3f**), 7.90 (d, J = 8.3 Hz, 1H, *cis*-**3f**), 7.75 (d, J =8.4 Hz, 1H, *trans*-**3f**), 7.67 (d, J =3.6 Hz, 1H, *cis*-**3f**), 7.65 (d, J =4.0 Hz, 1H, *cis*-**3f**), 7.64–7.57 (m, 2H, *cis*-**3f**; 1H, *trans*-**3f**), 7.56–7.47 (m, 3H, *cis*-**3f**; 3H, *trans*-**3f**), 7.21 (dd, J = 1.8, 1.8 Hz, 1H, *cis*-**3f**), 7.17 (dd, J = 1.8, 1.8 Hz, 1H, *cis*-**3f**), 7.10 (s, 1H, *trans*-**3f**), 7.09 (s, 1H, *trans*-**3f**), 6.91 (d, J = 8.1 Hz, 1H, *trans*-**3f**), 6.80 (d, J = 8.1 Hz, 1H, *trans*-**3f**), 5.75 (dd, J = 1.5, 1.5 Hz, 1H, *trans*-**3f**), 5.63 (dd, J = 1.6, 1.6 Hz, 1H, *cis*-**3f**), 3.56 (s, 3H, *trans*-**3f**), 3.49 (s, 3H, *cis*-**3f**), 3.38 (s, 3H, *cis*-**3f**), 3.15 (s, 3H, *trans*-**3f**); ^{13}C NMR ($CDCl_3$, 100 MHz) δ 165.79, 165.76, 143.9, 142.3, 141.4, 139.7, 138.74, 138.71 138.1, 137.3, 135.4, 134.9, 134.2, 133.8, 133.5, 133.2, 133.1, 132.5, 131.4, 131.1, 130.4, 130.2, 130.1, 128.8, 128.6, 128.5, 128.4, 128.0, 127.9, 127.3, 127.1, 127.0, 126.8, 126.7, 126.5, 126.3, 126.2, 126.1, 123.8, 52.4, 52.15, 52.08.

Identification of structural isomers and diastereomers

For the identification of **3** and **4**, isomers with the **H (bald)** of the *meta*-bridged benzene ring entering the shielded region by the *para*-bridged benzene ring in high field shifts below 6.0 ppm were designated **3**, and those without were designated **4**.

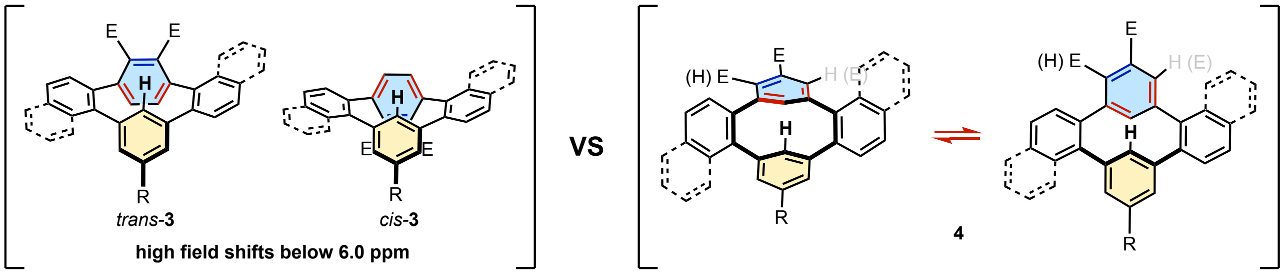

For the identification of *trans*-**3** and *cis*-**3**, isomers with the **H (bald)** of the *para*-bridged benzene ring entering the shielded region by the *meta*-bridged benzene ring in high field shifts below 7.0 ppm were designated *trans*-**3**, and those without were designated *cis*-**3**.

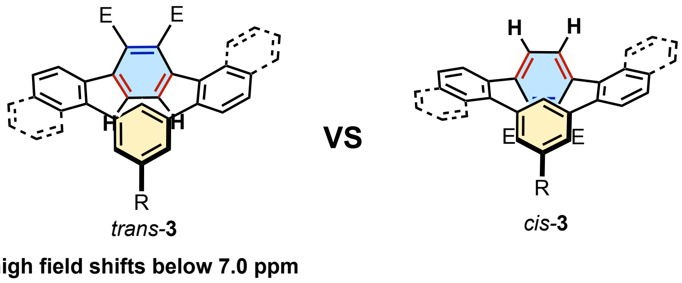

3. X-Ray Single Crystal Diffraction Analyses

Single crystals of **3a** suitable for an X-ray analysis were grown by diffusing *n*-hexane into a *tert*-butyl methyl ether solution.

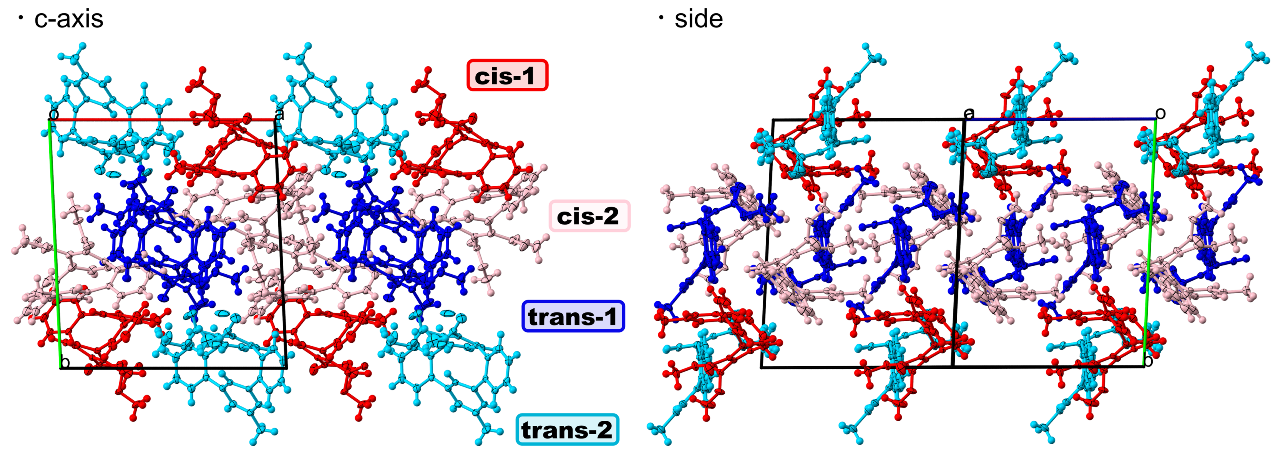

Figure S1. X-ray crystal packing structures of *cis*- and *trans*-**3a** showing thermal ellipsoids at the 50% probability level.

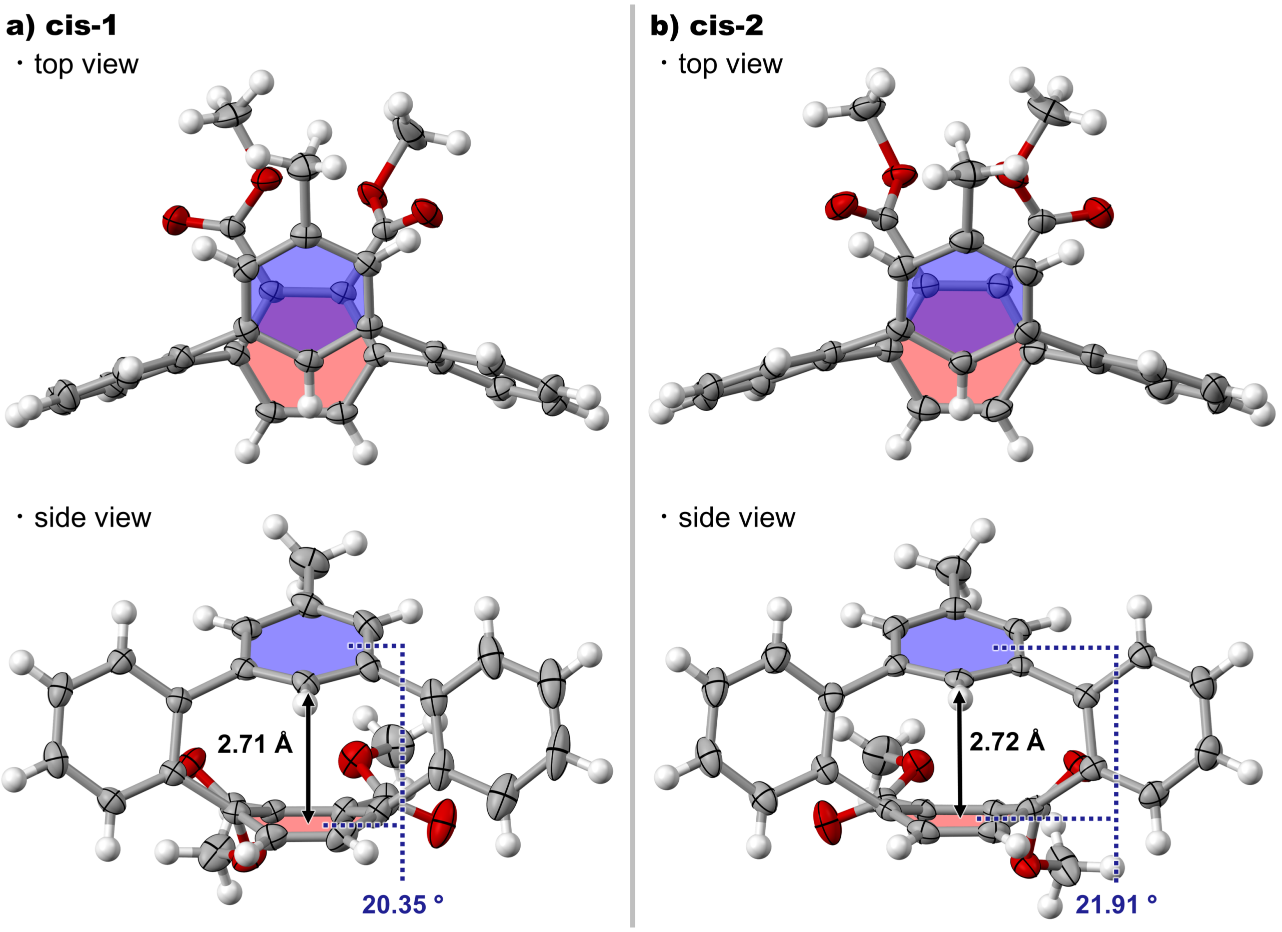

Figure S2. X-ray crystal structures of *cis*-**3a** showing thermal ellipsoids at the 50% probability level. The distances between the red plane and the edge carbon atom of the blue plane are indicated in black. The dihedral angles between the red plane and the blue plane are indicated in blue.

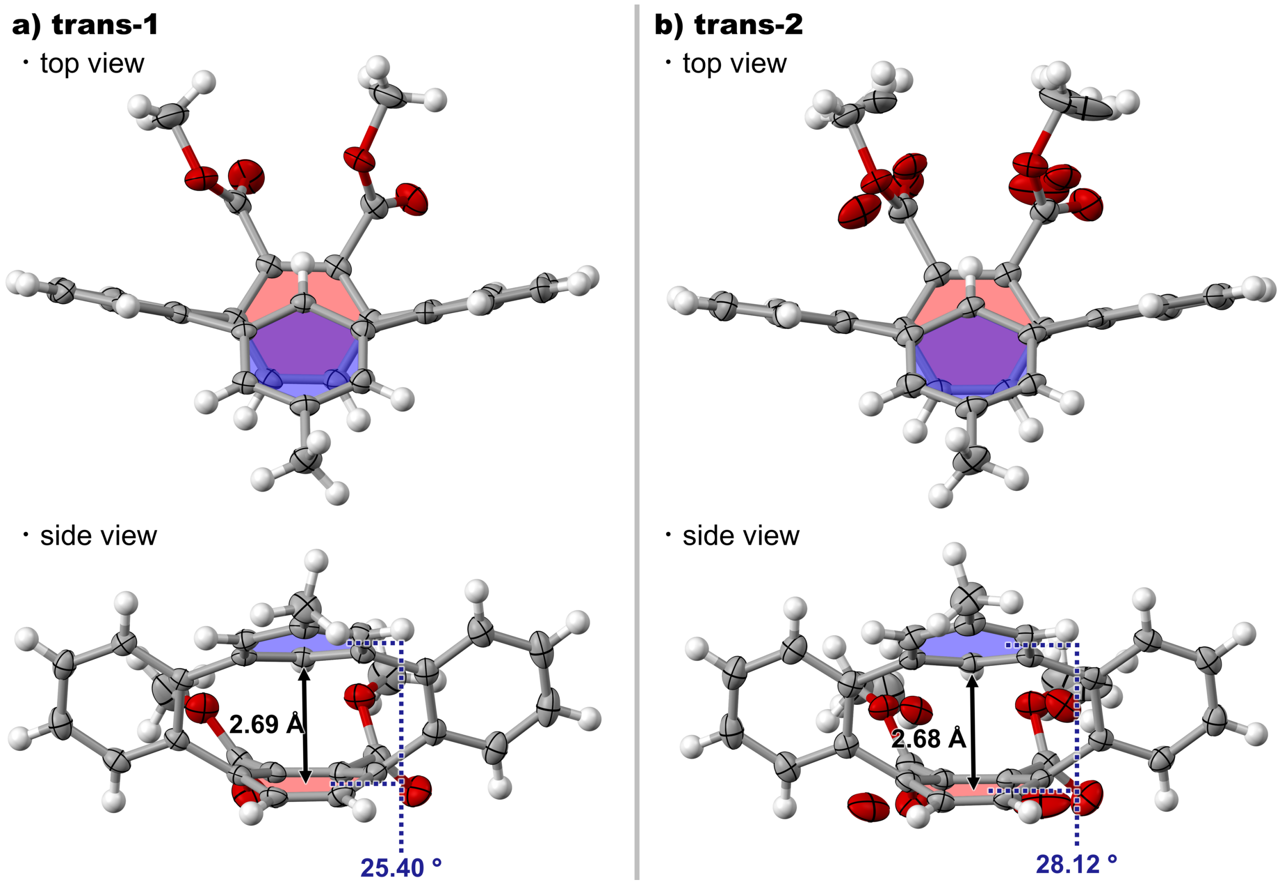

Figure S3. X-ray crystal structures of *trans*-**3a** showing thermal ellipsoids at the 50% probability level. The distances between the red plane and the edge carbon atom of the blue plane are indicated in black. The dihedral angles between the red plane and the blue plane are indicated in blue.

Table S1. Crystal data and data collection parameters of **3a**.

**3a** / CCDC 2362959	
Empirical formula	$C_{29}H_{22}O_4$
Formula weight	434.46
Colour	clear light yellow
Shape	plate
Temperature / K	169.99(10)
Wavelength / Å	0.71073
Crystal system	Triclinic
Space group	*P*-1
Unit cell dimensions	a = 15.1341(3) Å α = 88.603(2)°<br>b = 16.4252(3) Å β = 79.874(2)°<br>c = 18.4318(4) Å γ = 87.118(2)°
Volume / Å^3	4504.15(16)
Z	8
D_{calc} / g cm^{-3}	1.281
μ (Cu Kα) / mm^{-1}	0.085
F(000)	1824.0
Crystal size / mm^3	0.36 × 0.3 × 0.16
Theta range for data collection / °	3.374 to 58.262
Index ranges	-20<=h<=20, -22<=k<=22, -25<=l<=25
Reflections collected	94372
Independent reflections	23837 [R(int) = 0.0356, R(sigma) = 0.0536]
Data / restraints / parameters	23837 / 8 / 1259
Goodness-of-fit on F^2	1.040
Final R indices [I>2sigma(I)]	R_1 = 0.0678, wR_2 = 0.1420
R indices (all data)	R_1 = 0.1116, wR_2 = 0.1597
Largest diff. peak / hole / eÅ^{-3}	0.53 / -0.32

Alert level B

PLAT230_ALERT_2_B Hirshfeld Test Diff for C03H --C03J. 9.5 s.u.

Response: Due to low quality of the crystal, but this does not indicate an incorrect atom assignment.

Single crystals of *cis*-**3b** suitable for an X-ray analysis were grown by diffusing *n*-hexane into a CH_2Cl_2 solution.

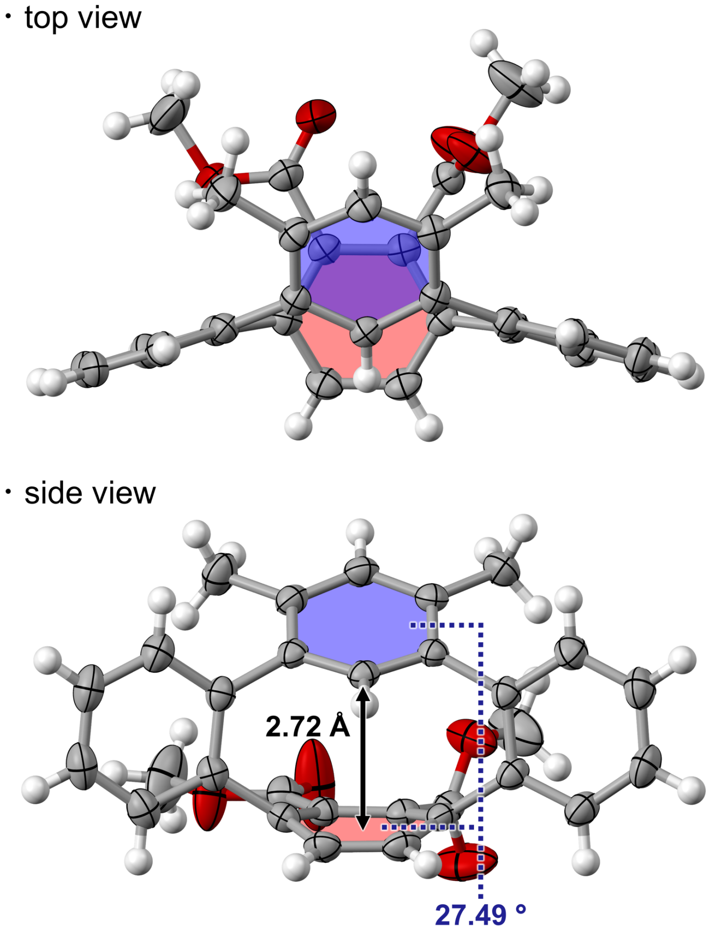

Figure S4. X-ray crystal structures of *cis*-**3b** showing thermal ellipsoids at the 50% probability level. The distance between the red plane and the edge carbon atom of the blue plane is indicated in black. The dihedral angle between the red plane and the blue plane is indicated in blue.

Table S2. Crystal data and data collection parameters of *cis*-**3b**.

cis-**3b** / CCDC 2362956	
Empirical formula	$C_{30}H_{24}O_4$
Formula weight	448.49
Colour	clear light yellow
Shape	plate
Temperature / K	293(1)
Wavelength / Å	0.71073
Crystal system	Monoclinic
Space group	$P2_1/n$
Unit cell dimensions	a = 12.4592(3) Å α = 90°<br>b = 9.3145(3) Å β = 92.315(3)°<br>c = 19.6308(5) Å γ = 90°
Volume / Å^3	2276.32(11)
Z	4
D_{calc} / g cm^{-3}	1.309
μ (Cu Kα) / mm^{-1}	0.086
F(000)	944.0
Crystal size / mm^3	0.4 × 0.2 × 0.2
Theta range for data collection / °	4.842 to 61.086
Index ranges	-17<=h<=17, -13<=k<=13, -28<=l<=26
Reflections collected	20044
Independent reflections	6689 [R(int) = 0.0199, R(sigma) = 0.0336]
Data / restraints / parameters	6689 / 0 / 311
Goodness-of-fit on F^2	1.028
Final R indices [I>2sigma(I)]	R_1 = 0.0593, wR_2 = 0.1466
R indices (all data)	R_1 = 0.0899, wR_2 = 0.1628
Largest diff. peak / hole / eÅ^{-3}	0.39 / -0.37

Single crystals of (±)-**3d** suitable for an X-ray analysis were grown by diffusing *n*-hexane into a CH_2Cl_2-EtOAc (1:2) solution.

・top view

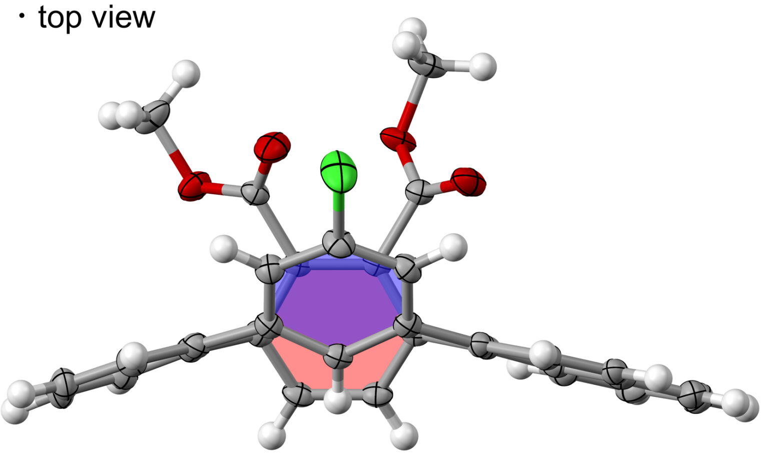

・side view

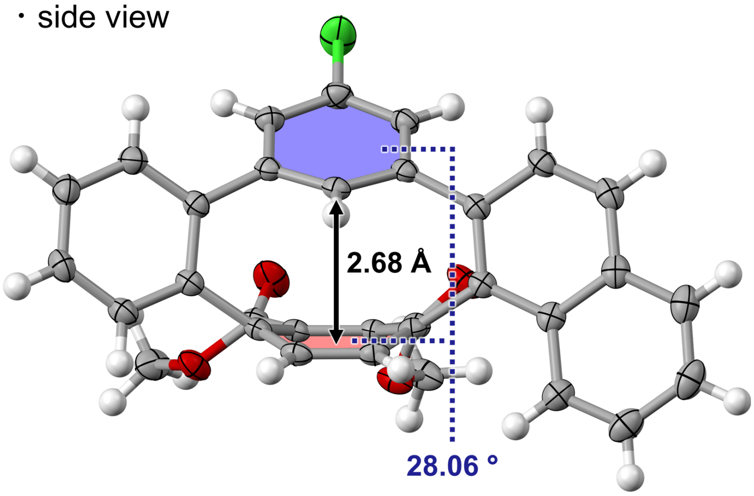

Figure S5. X-ray crystal structures of (±)-**3d** showing thermal ellipsoids at the 50% probability level. The distance between the red plane and the edge carbon atom of the blue plane is indicated in black. The dihedral angle between the red plane and the blue plane is indicated in blue.

Table S3. Crystal data and data collection parameters of (±)-**3d**.

(±)-**3d** / CCDC 2362957	
Empirical formula	$C_{32}H_{21}ClO_4$
Formula weight	504.94
Colour	clear light yellow
Shape	Plate
Temperature / K	169.99(10)
Wavelength / Å	0.71073
Crystal system	Triclinic
Space group	*P*-1
Unit cell dimensions	a = 9.4052(3) Å α = 101.164(3)°<br>b = 10.4009(3) Å β = 98.597(2)°<br>c = 12.7042(4) Å γ = 95.333(3)°
Volume / Å^3	1195.99(7)
Z	2
D_{calc} / g cm^{-3}	1.402
μ (Cu Kα) / mm^{-1}	0.199
F(000)	524.0
Crystal size / mm^3	0.3 × 0.25 × 0.2
Theta range for data collection / °	4.644 to 61.178
Index ranges	-13<=h<=13, -14<=k<=14, -18<=l<=17
Reflections collected	20957
Independent reflections	6889 [R(int) = 0.0221, R(sigma) = 0.0343]
Data / restraints / parameters	6889 / 0 / 336
Goodness-of-fit on F^2	1.042
Final R indices [I>2sigma(I)]	R_1 = 0.0427, wR_2 = 0.1044
R indices (all data)	R_1 = 0.0622, wR_2 = 0.1133
Largest diff. peak / hole / eÅ^{-3}	0.36 / -0.40

Single crystals of (±)-*cis*-**3e** suitable for an X-ray analysis were grown by diffusing *n*-hexane into a CH_2Cl_2-EtOAc (2:1) solution.

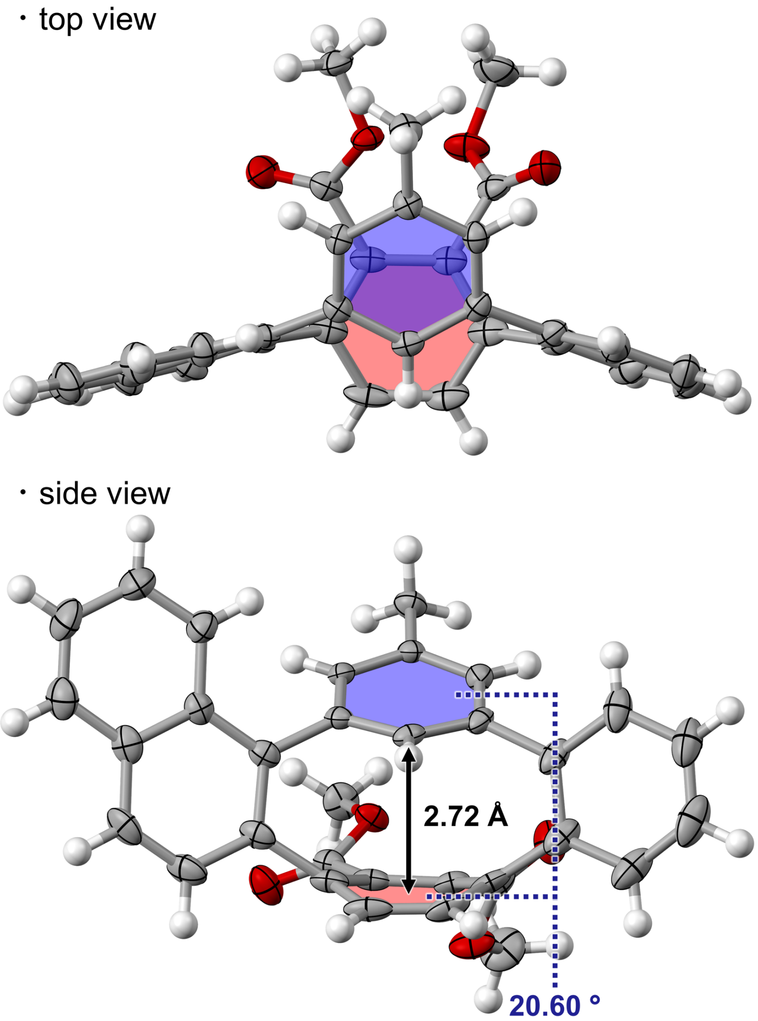

Figure S6. X-ray crystal structures of (±)-*cis*-**3e** showing thermal ellipsoids at the 50% probability level. The distance between the red plane and the edge carbon atom of the blue plane is indicated in black. The dihedral angle between the red plane and the blue plane is indicated in blue.

Table S4. Crystal data and data collection parameters of (±)-*cis*-**3e**.

(±)-*cis*-**3e** / CCDC 2362958	
Empirical formula	$C_{33}H_{24}O_4$
Formula weight	484.52
Colour	clear light yellow
Shape	Plate
Temperature / K	169.99(10)
Wavelength / Å	0.71073
Crystal system	Monoclinic
Space group	$P2_1/c$
	a = 10.6066(3) Å α = 90°
Unit cell dimensions	b = 22.2412(7) Å β = 91.652(3)°
	c = 10.3938(4) Å γ = 90°
Volume / $Å^3$	2450.92(14)
Z	4
D_{calc} / g cm^{-3}	1.313
μ (Cu Kα) / mm^{-1}	0.086
F(000)	1016.0
Crystal size / mm^3	0.25 × 0.15 × 0.07
Theta range for data collection / °	5.308 to 58.26
Index ranges	-14<=h<=14, -30<=k<=30, -14<=l<=14
Reflections collected	55087
Independent reflections	6607 [R(int) = 0.0536, R(sigma) = 0.0530]
Data / restraints / parameters	6607 / 0 / 337
Goodness-of-fit on F^2	1.140
Final R indices [I>2sigma(I)]	R_1 = 0.0972, wR_2 = 0.1835
R indices (all data)	R_1 = 0.1350, wR_2 = 0.1990
Largest diff. peak / hole / e$Å^{-3}$	0.45 / -0.27

Alert level B

PLAT230_ALERT_2_B Hirshfeld Test Diff for C00R --C00T. 8.0 s.u.

Response: Due to low quality of the crystal, but this does not indicate an incorrect atom assignment.

Single crystals of (±)-*cis*-**3f** suitable for an X-ray analysis were grown by diffusing *n*-hexane into a CH_2Cl_2 solution.

Table S5. Crystal data and data collection parameters of (±)-*cis*-**3f.**

	(±)-*cis*-**3f** / CCDC 2362970
Empirical formula	$C_{36}H_{23}ClO_4$
Formula weight	555.034
Colour	clear light yellow
Shape	plate
Temperature / K	100.15
Wavelength / Å	0.71073
Crystal system	Orthorhombic
Space group	*Pbca*
Unit cell dimensions	a = 12.9795(4) Å α = 90°<br>b = 15.1210(4) Å β = 90°<br>c = 27.3995(8) Å γ = 90°
Volume / Å^3	5377.5(3)
Z	8
D_{calc} / g cm^{-3}	1.371
μ (Cu Kα) / mm^{-1}	0.184
F(000)	2306.6
Crystal size / mm^3	0.4 × 0.3 × 0.2
Theta range for data collection / °	4.4 to 60.94
Index ranges	-17<=h<=18, -20<=k<=21, -38<=l<=38
Reflections collected	35847
Independent reflections	7939 [R(int) = 0.0272, R(sigma) = 0.0287]
Data / restraints / parameters	7939 / 0 / 372
Goodness-of-fit on F^2	1.037
Final R indices [I>2sigma(I)]	R_1 = 0.0576, wR_2 = 0.1406
R indices (all data)	R_1 = 0.0797, wR_2 = 0.1537
Largest diff. peak / hole / eÅ^{-3}	0.54 / -0.46

4. Theoretical Calculations

All calculations were carried out using the Gaussian 16 program,[4]. The hybrid density functional method based on B3LYP[5,6] with a 6-31g(d) basis set or M06[7] with a 6-31g(d) basis set were used for geometry optimizations. Harmonic vibrational analysis at the same level was performed to confirm the number of imaginary frequencies for all stationary points (0 for minima and 1 for TSs). The intrinsic reaction coordinate (IRC) method was used to track minimum energy paths from transition structures to the corresponding local minima. Cartesian coordinates of optimized structures are listed in Tables S6–S22. Zero-point energy, enthalpy, and Gibbs free energy at 298.15 K and 1 atm were calculated by M06/6-31G(d) level of theory for estimation of rotational barriers, and estimated from the gas-phase studies. The results are shown in Table S23. Strain energies in Table 2 were calculated according to the reported procedure[8]. Strain distributions of *cis*-**3a'**, *cis*-**3c'**, *cis*-**3e'**, and *cis*-**3f'** (Figures S7, S8, S9, and 5) were calculated using the StrainViz program[9] developed by the Jasti group and VMD (v1.9.3) program[10] for visualization.

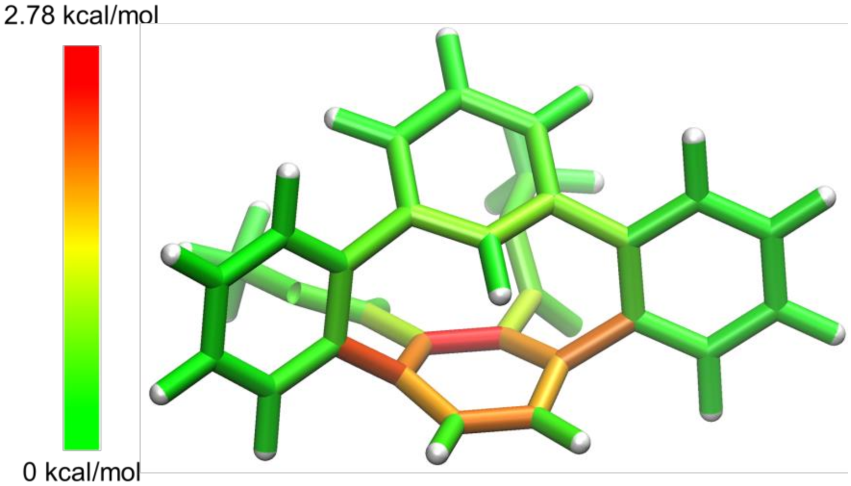

Figure S7. Strain analyses of *cis*-**3a'** by DFT calculations at the B3LYP/6-31G* level of theory.

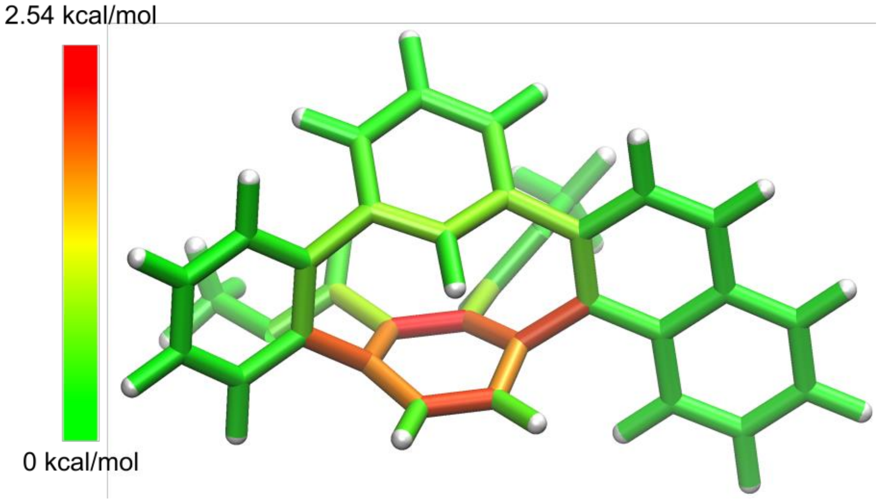

Figure S8. Strain analyses of *cis*-**3c'** by DFT calculations at the B3LYP/6-31G* level of theory.

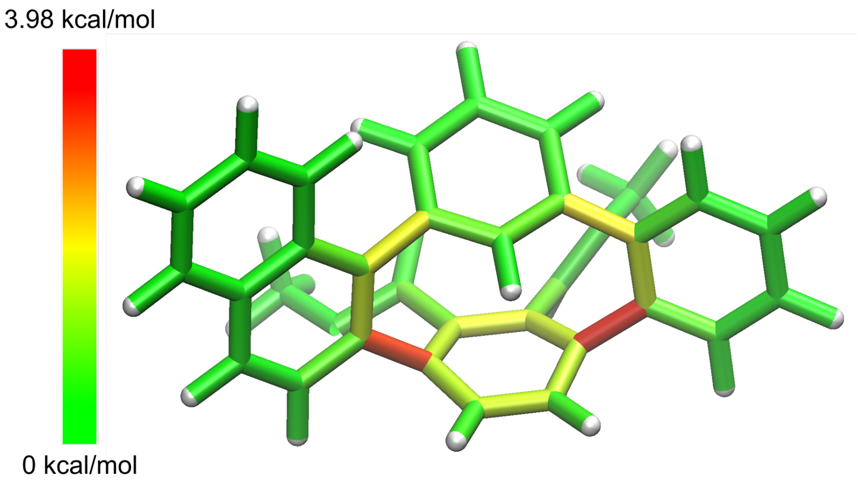

Figure S9. Strain analyses of *cis*-**3e'** by DFT calculations at the B3LYP/6-31G* level of theory.

Table S6. Cartesian coordinates of optimized *trans*-**3a'** [M06/6-31g(d) level of theory].

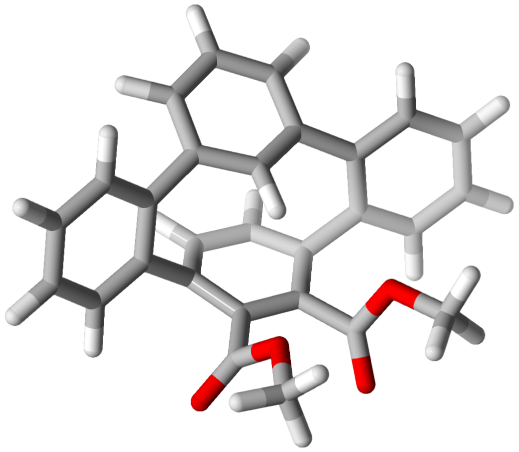

C	-1.29454	-3.46261	-0.46357	C	-0.81355	-0.87911	2.115121
C	-1.28189	-2.06971	-0.59946	C	0.571769	-0.90152	2.146893
C	-0.04303	-1.43653	-0.75386	C	1.301386	0.00782	1.376048
C	1.162722	-2.12005	-0.55079	C	0.639507	1.157157	0.925196
C	1.114682	-3.51165	-0.41658	C	-0.76057	1.201616	0.930223
C	-0.1047	-4.17987	-0.41953	C	-1.4738	2.328436	0.261715
C	-2.56319	-1.3241	-0.39947	O	-2.39129	2.945507	0.743313
C	-3.68947	-1.68887	-1.14136	O	-0.96366	2.571643	-0.95898
C	-4.92465	-1.08819	-0.92955	C	1.369626	2.273286	0.246887
C	-5.05951	-0.1046	0.042412	O	1.377365	3.414104	0.643546
C	-3.9561	0.275784	0.797777	O	1.986609	1.856404	-0.86412
C	-2.71376	-0.32302	0.599999	C	2.692259	2.868282	-1.57148
C	2.456337	-1.42147	-0.27735	C	-1.4462	3.755808	-1.58734
C	2.569923	-0.43433	0.73656	H	-2.24694	-3.97726	-0.33558
C	3.810508	0.124273	1.027411	H	-0.01701	-0.36331	-0.92651
C	4.950331	-0.27087	0.336229	H	2.039713	-4.06404	-0.25028
C	4.850063	-1.2366	-0.65845	H	-0.12903	-5.26294	-0.30978
C	3.61653	-1.8073	-0.95183	H	-3.58099	-2.4528	-1.91149
C	-1.482	0.071694	1.341083	H	-5.7821	-1.38778	-1.52991

H	-6.02303	0.371897	0.213516	H	1.086611	-1.75858	2.580679
H	-4.04457	1.05707	1.549982	H	3.118708	2.379041	-2.44918
H	3.874164	0.886297	1.804993	H	2.007342	3.671215	-1.8694
H	5.914301	0.174831	0.575276	H	3.48538	3.29368	-0.94613
H	5.735423	-1.54942	-1.2095	H	-0.99556	3.77317	-2.58184
H	3.538881	-2.56126	-1.73549	H	-2.53825	3.739771	-1.6585
H	-1.37294	-1.727	2.510266	H	-1.13452	4.633686	-1.00886

Table S7. Cartesian coordinates of optimized TS-**3a’** [M06/6-31g(d) level of theory].

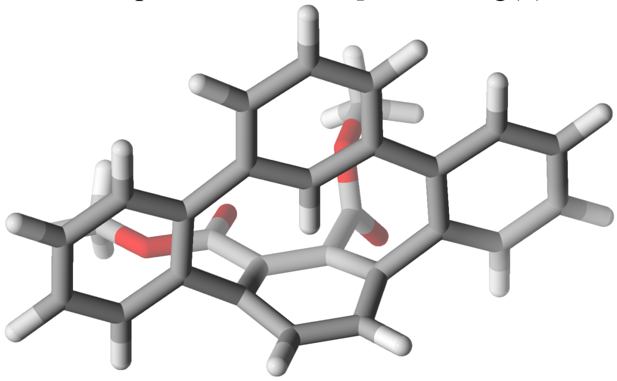

C	-1.35067	-3.056	-1.35757	O	-2.16713	3.156115	0.093161
C	-1.32978	-1.96755	-0.47368	O	-1.60389	1.610642	-1.45284
C	-0.05636	-1.50683	-0.14786	C	1.339447	2.31011	-0.36059
C	1.171726	-2.00808	-0.57009	O	0.727063	3.133185	-1.0052
C	1.089775	-3.08667	-1.46315	O	2.669511	2.18134	-0.41964
C	-0.15528	-3.58351	-1.83876	C	3.323301	3.053125	-1.3345
C	-2.5643	-1.32338	0.098907	C	-2.34598	2.351934	-2.41522
C	-3.81063	-1.83777	-0.28526	H	-2.2799	-3.51115	-1.69278
C	-5.01824	-1.31582	0.157056	H	-0.01747	-0.6853	0.503371
C	-5.03325	-0.2285	1.017888	H	1.97489	-3.55803	-1.88437
C	-3.82187	0.31057	1.429203	H	-0.19578	-4.42191	-2.53266
C	-2.60169	-0.21163	1.00482	H	-3.85019	-2.68331	-0.96729
C	2.465497	-1.44	-0.0492	H	-5.95052	-1.76347	-0.18319
C	2.614106	-0.32754	0.84204	H	-5.97203	0.198926	1.3642
C	3.878081	0.033555	1.306197	H	-3.80119	1.175457	2.09172
C	5.026568	-0.63471	0.912165	H	3.944511	0.884479	1.983645
C	4.906834	-1.69986	0.02962	H	5.999979	-0.32707	1.289369
C	3.655436	-2.08313	-0.42657	H	5.788399	-2.24681	-0.30037
C	-1.34062	0.423539	1.486924	H	3.606341	-2.93811	-1.09591
C	-0.61373	-0.1406	2.540192	H	-1.12327	-0.78127	3.259423
C	0.775552	-0.14393	2.474023	H	1.342758	-0.80058	3.1334
C	1.41991	0.408357	1.359929	H	4.38423	2.802064	-1.27478
C	0.710962	1.384379	0.618926	H	2.948263	2.896821	-2.35151
C	-0.68882	1.373949	0.686037	H	3.157102	4.099302	-1.05563
C	-1.54371	2.182555	-0.24595	H	-2.30835	1.76947	-3.33776

H	-3.3823	2.485666	-2.08621	H	-1.88347	3.335172	-2.55984

Table S8. Cartesian coordinates of optimized *cis*-**3a'** [M06/6-31g(d) level of theory].

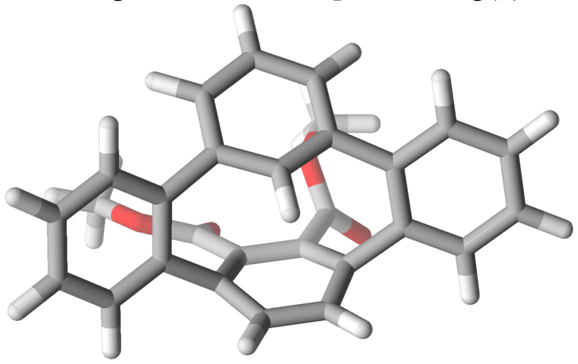

C	-1.27744	-0.33015	-2.50722	O	-1.31116	2.085899	-0.68511
C	-1.29191	-1.0597	-1.31396	C	1.344933	1.980015	0.657453
C	-0.06619	-1.5109	-0.81671	O	0.846376	3.080627	0.742496
C	1.158427	-1.07129	-1.32911	O	2.577803	1.770075	0.180407
C	1.13823	-0.31845	-2.50696	C	3.269896	2.941919	-0.23475
C	-0.07207	0.002382	-3.11495	C	-1.94995	3.233652	-1.22671
C	-2.56267	-1.19979	-0.54019	H	-2.21561	0.046542	-2.91643
C	-3.72351	-1.62567	-1.19102	H	-0.06284	-2.15209	0.057093
C	-4.93977	-1.71765	-0.52502	H	2.075995	0.05408	-2.92037
C	-5.02385	-1.37483	0.819241	H	-0.07533	0.586665	-4.03409
C	-3.88602	-0.94057	1.489605	H	-3.6575	-1.90936	-2.24155
C	-2.66239	-0.84137	0.833514	H	-5.82381	-2.06227	-1.05884
C	2.43809	-1.2751	-0.587	H	-5.97443	-1.43925	1.34547
C	2.580978	-0.91183	0.778646	H	-3.94	-0.64926	2.538155
C	3.797482	-1.10558	1.424413	H	3.883478	-0.82919	2.475329
C	4.894648	-1.61934	0.740854	H	5.842059	-1.75802	1.258807
C	4.774044	-1.95049	-0.60305	H	5.62583	-2.35546	-1.14683
C	3.556484	-1.77821	-1.25315	H	3.454843	-2.06046	-2.30131
C	-1.41114	-0.43405	1.528801	H	-1.24519	-2.23157	2.694495
C	-0.71158	-1.37149	2.288597	H	1.225477	-2.22913	2.653338
C	0.678685	-1.36733	2.269608	H	4.251676	2.603109	-0.5713
C	1.360286	-0.43598	1.483233	H	2.73247	3.435924	-1.05159
C	0.667356	0.737911	1.120267	H	3.36929	3.647957	0.596676
C	-0.73072	0.728629	1.143094	H	-1.71807	3.228642	-2.29488
C	-1.54692	1.869385	0.614078	H	-3.03255	3.191767	-1.06652
O	-2.36129	2.465817	1.272933	H	-1.55013	4.137297	-0.75108

Table S9. Cartesian coordinates of optimized *trans*-**3c'** [M06/6-31g(d) level of theory].

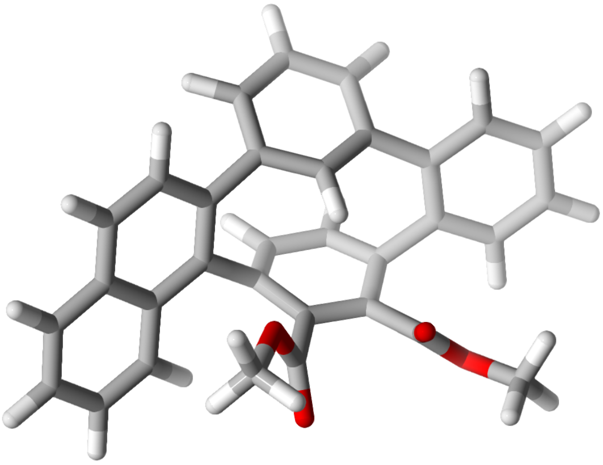

C	-0.20573	3.719226	0.179626	O	2.66658	-3.0104	-0.35937
C	-0.36833	2.367718	0.500912	C	3.404706	-3.89963	0.474671
C	0.780333	1.599571	0.72868	C	-2.51035	-2.1922	2.126628
C	2.056108	2.083229	0.417693	H	-1.08692	4.335076	-0.0022
C	2.185717	3.438324	0.092416	H	0.669986	0.567837	1.054176
C	1.065714	4.258653	0.020924	H	3.169526	3.834705	-0.15926
C	-1.73568	1.772088	0.407362	H	1.180365	5.310978	-0.23426
C	-2.79566	2.359603	1.142363	H	-2.56514	3.19606	1.801893
C	-4.07337	1.873541	1.068004	H	-4.87129	2.322158	1.660253
C	-4.38236	0.776807	0.230358	C	-5.68943	0.240864	0.149878
C	-3.34167	0.18767	-0.54367	C	-3.65577	-0.91496	-1.37696
C	-2.02002	0.698962	-0.44087	H	4.376097	-1.47318	-1.5689
C	3.24541	1.195489	0.234322	H	6.502584	-0.8384	-0.45594
C	3.228811	0.0827	-0.65117	H	6.539828	1.088729	1.119247
C	4.404804	-0.62844	-0.88331	H	4.470076	2.38122	1.533604
C	5.594061	-0.27246	-0.25737	H	-0.57313	1.615386	-2.55647
C	5.615772	0.807096	0.617198	H	1.870664	1.34266	-2.64465
C	4.453994	1.533107	0.849044	H	4.070505	-4.45197	-0.19135
C	-0.85356	0.054535	-1.10622	H	3.984667	-3.32968	1.210299
C	-0.10694	0.775191	-2.04221	H	2.731438	-4.58302	1.001515
C	1.269362	0.616087	-2.0982	H	-2.85958	-1.54143	2.930751
C	1.907706	-0.28495	-1.24248	H	-3.35237	-2.50648	1.497097
C	1.119027	-1.26836	-0.62563	H	-2.01005	-3.07786	2.531724
C	-0.26443	-1.08359	-0.53903	H	-5.15375	-2.26837	-2.0694
C	-1.06449	-2.02455	0.307447	H	-6.47715	0.700456	0.747591
O	-1.22357	-3.18966	0.034387	C	-5.96105	-0.83444	-0.65748
O	-1.59866	-1.40789	1.363557	H	-2.86419	-1.36734	-1.97419
C	1.746457	-2.27649	0.277026	C	-4.93234	-1.41676	-1.4288
O	1.470245	-2.40193	1.447621	H	-6.96985	-1.24029	-0.70796

Table S10. Cartesian coordinates of optimized TS-**3c’** [M06/6-31g(d) level of theory].

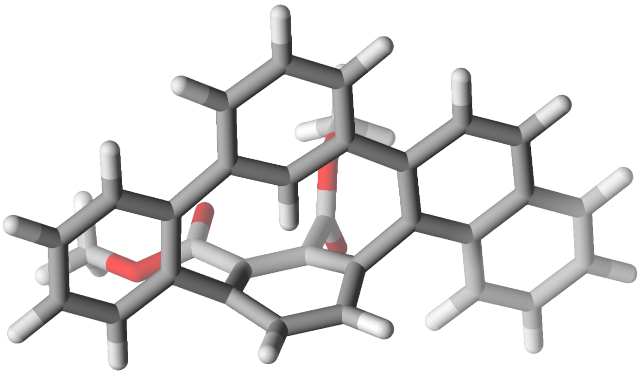

C	-0.2206	3.425464	1.282404	O	3.068836	-2.51424	0.284558
C	-0.4296	2.249715	0.546006	C	3.806512	-3.29038	1.221914
C	0.738347	1.613844	0.129739	C	-1.80204	-1.74602	3.042691
C	2.054666	2.01946	0.324184	H	-1.0432	4.01614	1.67806
C	2.20286	3.197891	1.070953	H	0.612938	0.722106	-0.40668
C	1.075374	3.867827	1.536479	H	3.178675	3.616018	1.307493
C	-1.78863	1.698713	0.207209	H	1.211295	4.778685	2.117727
C	-2.91616	2.446928	0.649011	H	-2.75547	3.376286	1.187693
C	-4.20766	2.0608	0.425541	H	-5.03267	2.677099	0.783742
C	-4.49823	0.86482	-0.26295	C	-5.82251	0.430242	-0.49932
C	-3.40612	0.080614	-0.7273	C	-3.69191	-1.1327	-1.40586
C	-2.06656	0.51097	-0.50485	H	4.140335	-1.43047	-2.19035
C	3.199459	1.248761	-0.27786	H	6.387098	-0.41277	-1.88938
C	3.108098	0.042208	-1.04732	H	6.59467	1.664269	-0.51114
C	4.254931	-0.51389	-1.61282	H	4.623423	2.688558	0.457307
C	5.510385	0.046912	-1.43713	H	-0.89735	0.65232	-2.97082
C	5.623246	1.199951	-0.67226	H	1.552887	0.422442	-3.19465
C	4.491212	1.775326	-0.11706	H	4.813128	-3.37735	0.80797
C	-0.95109	-0.31839	-1.05	H	3.834585	-2.79348	2.197403
C	-0.34303	0.042398	-2.25766	H	3.352027	-4.27952	1.343745
C	1.034804	-0.09734	-2.38899	H	-1.81288	-0.99419	3.833917
C	1.785092	-0.60154	-1.32006	H	-2.82283	-2.03993	2.775343
C	1.107998	-1.40595	-0.37308	H	-1.24144	-2.6329	3.359415
C	-0.27756	-1.24686	-0.23971	H	-5.18392	-2.47212	-2.13157
C	-1.0413	-1.92282	0.860882	H	-6.64495	1.048477	-0.13846
O	-1.5504	-3.00845	0.736713	C	-6.06555	-0.74441	-1.16423
O	-1.14721	-1.13754	1.933726	H	-2.86558	-1.7572	-1.74305
C	1.808228	-2.23808	0.641421	C	-4.98672	-1.53396	-1.61627
O	1.300221	-2.66249	1.655532	H	-7.08805	-1.07369	-1.34031

Table S11. Cartesian coordinates of optimized *cis*-**3c'** [M06/6-31g(d) level of theory].

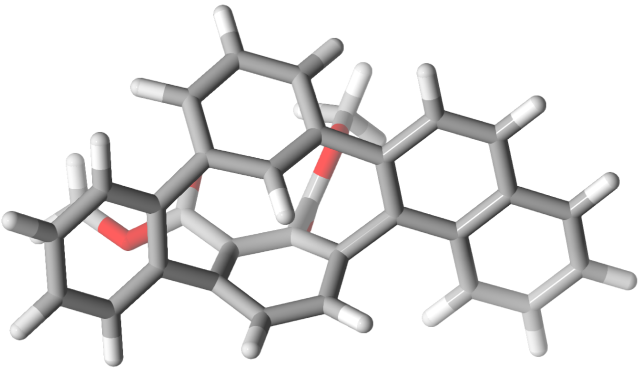

C	-0.17324	0.426528	-2.79469	O	3.273953	1.587022	1.30027
C	-0.43838	-0.56089	-1.8393	C	4.201685	2.574896	0.868581
C	0.650382	-1.23689	-1.2838	C	-1.75595	3.545494	-0.76401
C	1.969481	-0.81349	-1.48247	H	-1.00331	0.979143	-3.2355
C	2.201179	0.181985	-2.43525	H	0.463582	-2.05169	-0.59246
C	1.135905	0.763459	-3.11628	H	3.217536	0.542517	-2.5985
C	-1.83399	-0.73971	-1.33604	H	1.327035	1.545742	-3.84871
C	-2.87838	-0.96032	-2.26693	H	-2.61628	-1.06893	-3.31923
C	-4.18537	-1.06392	-1.86905	H	-4.97256	-1.25042	-2.59997
C	-4.5392	-0.93319	-0.50739	C	-5.88132	-1.02788	-0.06838
C	-3.51095	-0.69217	0.449928	C	-3.87377	-0.53399	1.811183
C	-2.16148	-0.59819	0.017665	H	3.963003	-1.56679	2.685198
C	3.081395	-1.27801	-0.60049	H	6.050791	-2.43235	1.650122
C	2.990062	-1.2019	0.813954	H	6.248526	-2.5268	-0.8284
C	4.054152	-1.63227	1.601015	H	4.350068	-1.80405	-2.24621
C	5.224434	-2.10719	1.020425	H	-1.21117	-2.42656	1.765752
C	5.335728	-2.1568	-0.36461	H	1.213809	-2.68821	2.154158
C	4.273321	-1.74439	-1.16034	H	5.13897	2.34961	1.380866
C	-1.02683	-0.4386	0.96981	H	4.336614	2.520604	-0.21816
C	-0.54599	-1.57237	1.632721	H	3.849013	3.578133	1.130193
C	0.816409	-1.71744	1.856698	H	-2.12666	3.529916	-1.79128
C	1.69924	-0.73864	1.39273	H	-2.55553	3.828562	-0.07123
C	1.179153	0.541709	1.136385	H	-0.92625	4.254193	-0.66342
C	-0.1996	0.692299	0.945942	H	-5.44583	-0.5016	3.254091
C	-0.76647	2.061146	0.716169	H	-6.65821	-1.21687	-0.80973
O	-0.77138	2.898192	1.585563	C	-6.19986	-0.88184	1.257353
O	-1.29621	2.224881	-0.49638	H	-3.0919	-0.32639	2.54137
C	2.054329	1.683465	0.753718	C	-5.18441	-0.62791	2.204961
O	1.720472	2.591094	0.025189	H	-7.23604	-0.95518	1.582754

Table S12. Cartesian coordinates of optimized *trans*-**3e'** [M06/6-31g(d) level of theory].

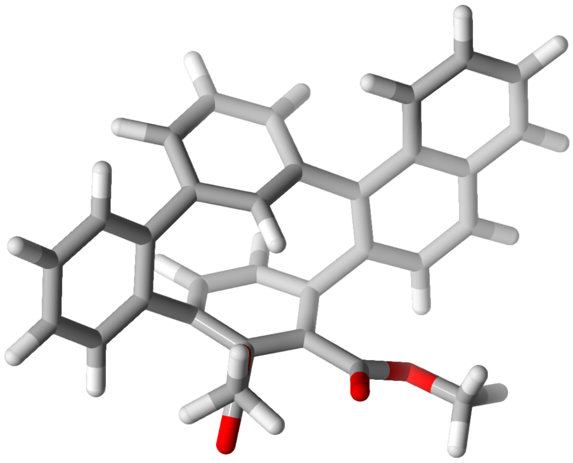

C	1.034316	3.501149	0.355346	O	-1.2781	-3.30727	0.479391
C	1.320818	2.229254	-0.14544	C	-1.8336	-4.37368	-0.28171
C	0.252595	1.354938	-0.39601	C	3.331572	-1.99526	-2.49646
C	-1.04277	1.640528	0.046314	C	-5.77903	0.225901	-0.57828
C	-1.29339	2.920823	0.562287	C	-3.64974	1.874772	-1.30162
C	-0.27333	3.856224	0.669975	C	-4.89458	2.070364	-1.84509
C	2.749859	1.793482	-0.19085	C	-5.97593	1.244085	-1.47451
C	3.690835	2.520009	-0.92057	H	1.853874	4.191077	0.557653
C	5.03622	2.165942	-0.91663	H	0.463594	0.384323	-0.83839
C	5.466838	1.076945	-0.16859	H	-2.29401	3.163611	0.919549
C	4.548366	0.34526	0.577797	H	-0.48655	4.846147	1.070294
C	3.201438	0.695028	0.585417	H	3.351062	3.367206	-1.51676
C	-2.12045	0.609096	0.211454	H	5.748668	2.742111	-1.50468
C	-1.93861	-0.5018	1.046013	H	6.518147	0.794797	-0.1636
C	-3.03976	-1.31582	1.397039	H	4.871667	-0.51752	1.159945
C	-4.2894	-1.06836	0.899713	H	-2.86999	-2.14671	2.077804
C	-4.50398	-0.0087	-0.01055	H	-5.13531	-1.69258	1.188464
C	-3.41255	0.839811	-0.36076	H	1.769345	1.333934	2.83767
C	2.149479	-0.09679	1.277985	H	-0.61761	0.765909	3.016344
C	1.377987	0.477144	2.289273	H	-2.78658	-4.6135	0.19574
C	0.03433	0.14782	2.399169	H	-1.99303	-4.06477	-1.32004
C	-0.54424	-0.76744	1.519128	H	-1.16675	-5.24245	-0.2692
C	0.320398	-1.62142	0.800222	H	3.202009	-1.40369	-3.40466
C	1.665021	-1.25528	0.663561	H	4.384073	-2.0048	-2.19059
C	2.565933	-1.96487	-0.30561	H	2.99071	-3.02513	-2.65297
O	3.283294	-2.88866	-0.01475	H	-6.60185	-0.429	-0.29024
O	2.528277	-1.36966	-1.50272	H	-2.81956	2.51016	-1.60393
C	-0.15288	-2.77479	-0.01244	H	-5.04769	2.866043	-2.57213
O	0.418478	-3.2223	-0.98218	H	-6.96042	1.411738	-1.9077

Table S13. Cartesian coordinates of optimized TS-**3e'** [M06/6-31g(d) level of theory].

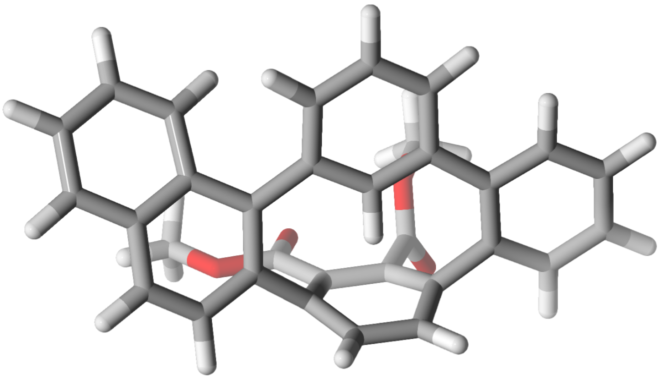

C	1.348321	3.130527	-0.9308	O	-1.82036	-2.54099	-0.26451
C	1.602434	1.993491	-0.1499	C	-2.56166	-3.22779	-1.26535
C	0.46749	1.305196	0.271554	C	2.765469	-1.48821	-3.03281
C	-0.86923	1.574577	-0.03717	C	-5.76092	0.614662	0.371818
C	-1.06052	2.793141	-0.71416	C	-3.6697	1.48675	-1.23111
C	0.035577	3.530643	-1.1553	C	-4.97258	1.675294	-1.62361
C	2.986816	1.528354	0.212797	C	-6.03827	1.27887	-0.79356
C	4.066443	2.361403	-0.11242	H	2.150563	3.72894	-1.35686
C	5.389968	2.016124	0.124779	H	0.636635	0.509163	0.937154
C	5.697895	0.797663	0.711559	H	-2.05094	3.204719	-0.87924
C	4.65785	-0.04829	1.073271	H	-0.14596	4.455623	-1.70072
C	3.324965	0.290064	0.85107	H	3.871962	3.331544	-0.56308
C	-1.97396	0.675368	0.453977	H	6.181446	2.709837	-0.15387
C	-1.79684	-0.37051	1.39327	H	6.73114	0.509458	0.89409
C	-2.88894	-0.88328	2.125479	H	4.867852	-1.01061	1.539718
C	-4.16846	-0.47387	1.88304	H	-2.67896	-1.63346	2.887827
C	-4.42736	0.357355	0.77139	H	-5.00342	-0.84583	2.476364
C	-3.33585	0.881151	0.010701	H	2.234378	0.101855	3.345719
C	2.253419	-0.63269	1.32227	H	-0.20464	-0.19827	3.602588
C	1.673	-0.4332	2.580038	H	-3.60078	-3.2038	-0.93048
C	0.300086	-0.61283	2.729878	H	-2.46024	-2.7237	-2.23259
C	-0.46066	-0.98444	1.617338	H	-2.2104	-4.26038	-1.36699
C	0.202445	-1.66784	0.56687	H	2.539379	-0.70694	-3.761
C	1.577708	-1.46594	0.417665	H	3.848121	-1.59509	-2.90605
C	2.330892	-1.92482	-0.7971	H	2.343383	-2.44797	-3.35257
O	3.067988	-2.87757	-0.80614	H	-6.5659	0.233265	1.000661
O	2.151956	-1.07808	-1.81532	H	-2.87661	1.741535	-1.927
C	-0.508	-2.42395	-0.49929	H	-5.17922	2.116972	-2.59704
O	0.039533	-2.9272	-1.45587	H	-7.0677	1.456054	-1.09912

Table S14. Cartesian coordinates of optimized *cis*-**3e’** [M06/6-31g(d) level of theory].

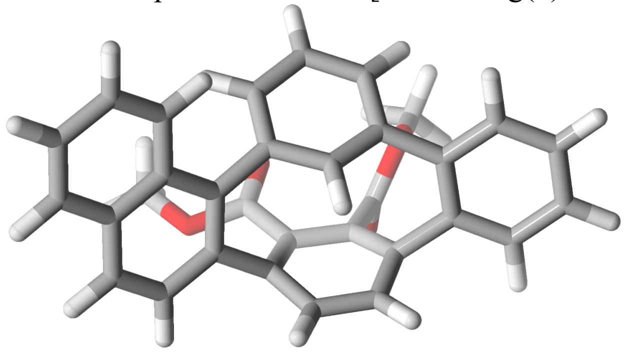

C	1.382707	1.142656	-2.14606	O	-1.39099	-2.89028	0.091165
C	1.375899	1.53064	-0.80325	C	-1.96811	-3.72551	-0.90504
C	0.156649	1.49736	-0.11753	C	3.862997	-1.95287	-2.03105
C	-0.9807	0.888225	-0.65958	C	-5.87886	0.91522	0.345584
C	-0.93148	0.481365	-1.99767	C	-3.73144	1.862457	-1.16166
C	0.227576	0.655675	-2.74582	C	-5.00983	2.278774	-1.4361
C	2.676565	1.78716	-0.11175	C	-6.09941	1.79552	-0.68159
C	3.555048	2.755764	-0.59634	H	2.323486	1.151322	-2.69654
C	4.799219	2.965018	-0.00848	H	0.120845	1.851433	0.908528
C	5.191855	2.196557	1.079724	H	-1.7972	-0.01478	-2.4381
C	4.337161	1.215862	1.574396	H	0.252647	0.332392	-3.78493
C	3.095548	0.992071	0.989507	H	3.243438	3.366284	-1.44415
C	-2.14637	0.491439	0.193175	H	5.460291	3.735505	-0.40175
C	-1.96468	-0.37971	1.270666	H	6.163193	2.356455	1.544291
C	-3.06471	-0.81463	2.041069	H	4.637135	0.600407	2.422407
C	-4.33641	-0.41175	1.736348	H	-2.87902	-1.49747	2.86965
C	-4.57132	0.472357	0.658481	H	-5.18638	-0.76175	2.322519
C	-3.46832	0.936879	-0.1194	H	1.701524	1.182445	3.283551
C	2.101967	0.021019	1.522874	H	-0.66845	0.503059	3.410844
C	1.325365	0.404755	2.617855	H	-2.9427	-4.02569	-0.51548
C	-0.00682	0.019288	2.691698	H	-2.08443	-3.17504	-1.8458
C	-0.5674	-0.72488	1.652026	H	-1.33759	-4.60242	-1.08666
C	0.305916	-1.44059	0.814395	H	4.120275	-1.2854	-2.85667
C	1.655393	-1.07275	0.766857	H	4.76845	-2.30685	-1.52691
C	2.609181	-1.85758	-0.08446	H	3.292513	-2.81513	-2.39444
O	2.959556	-2.97165	0.217929	H	-6.7087	0.543058	0.947165
O	3.054574	-1.1848	-1.14636	H	-2.89652	2.250756	-1.74175
C	-0.18945	-2.3852	-0.22231	H	-5.18459	2.992315	-2.23961
O	0.407944	-2.67261	-1.23582	H	-7.1095	2.130228	-0.91095

Table S15. Cartesian coordinates of optimized *trans*-**3f’** [M06/6-31g(d) level of theory].

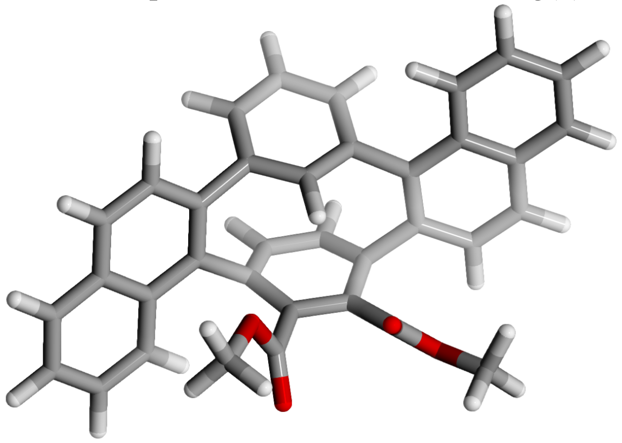

C	-0.35086	3.536306	-0.39388	C	-6.18674	0.877017	0.54492
C	-0.63579	2.282265	0.152965	C	-4.49855	-0.65221	-1.07256
C	0.429025	1.401916	0.392563	C	6.39084	0.042325	0.502616
C	1.709337	1.658349	-0.11003	C	4.366274	1.846163	1.157625
C	1.958957	2.917426	-0.67282	C	-6.68022	-0.20818	-0.13371
C	0.947468	3.864345	-0.76847	C	-5.82608	-0.97918	-0.95143
C	-2.07001	1.876018	0.26643	C	5.625794	1.996625	1.680345
C	-2.95661	2.656504	1.048297	C	6.654283	1.091212	1.344319
C	-4.28579	2.341492	1.147091	H	-1.16797	4.233031	-0.58333
C	-4.8216	1.236406	0.445886	H	0.229186	0.447689	0.87637
C	-3.95697	0.456031	-0.37465	H	2.948687	3.133573	-1.07514
C	-2.57885	0.790983	-0.45002	H	1.157472	4.839757	-1.20451
C	2.754797	0.59663	-0.27936	H	-2.55035	3.50411	1.599975
C	2.509021	-0.52367	-1.08348	H	-4.9506	2.937159	1.773122
C	3.552369	-1.42728	-1.38846	H	3.327773	-2.27907	-2.02698
C	4.815305	-1.23739	-0.89612	H	5.618802	-1.93005	-1.14754
C	5.09905	-0.14571	-0.04503	H	-1.26372	1.333085	-2.77616
C	4.061688	0.78169	0.270438	H	1.107961	0.742717	-3.06681
C	-1.5754	-0.04972	-1.16069	H	2.892103	-5.01209	-0.23503
C	-0.84886	0.48827	-2.22674	H	3.040059	-3.77441	1.059842
C	0.485887	0.150203	-2.39612	H	1.653293	-4.89818	1.063471
C	1.097363	-0.74763	-1.51871	H	-3.30882	-1.0466	3.19835
C	0.26688	-1.56318	-0.73426	H	-4.07021	-2.0438	1.911523
C	-1.06814	-1.19765	-0.5388	H	-2.70018	-2.70505	2.852047
C	-1.88256	-1.95533	0.464157	H	-6.83876	1.481388	1.176285
O	-2.21089	-3.10736	0.314638	H	-3.84152	-1.24904	-1.70506
O	-2.22268	-1.19414	1.506269	H	7.171941	-0.67274	0.242935
C	0.859033	-2.5575	0.206862	H	3.576288	2.541879	1.433649
O	0.677802	-2.54855	1.402612	H	-7.73151	-0.47675	-0.04627
O	1.631069	-3.44697	-0.42732	H	-6.22372	-1.83757	-1.48962
C	2.344654	-4.3377	0.426427	H	5.832244	2.817659	2.364751
C	-3.13196	-1.79485	2.42317	H	7.650912	1.223235	1.761757

Table S16. Cartesian coordinates of optimized TS-**3f'** [M06/6-31g(d) level of theory].

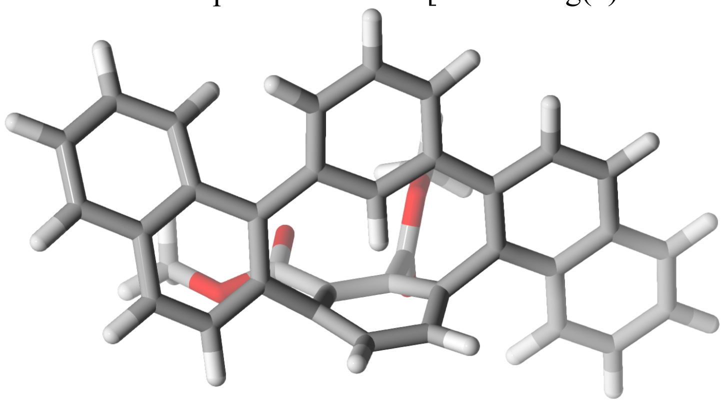

C	-0.49549	3.32977	0.834327	C	-6.46171	0.817229	-0.36904
C	-0.85931	2.141923	0.182221	C	-4.56231	-1.12342	-1.0248
C	0.210793	1.360609	-0.24655	C	6.37207	0.324187	-0.7046
C	1.576446	1.586839	-0.0623	C	4.438924	1.431342	0.952181
C	1.874513	2.847159	0.487821	C	-6.86421	-0.42758	-0.78107
C	0.848304	3.676302	0.934068	C	-5.90203	-1.40742	-1.10654
C	-2.28457	1.717734	-0.043	C	5.772896	1.572925	1.24812
C	-3.29785	2.669189	0.263736	C	6.759254	1.054581	0.387695
C	-4.63395	2.401847	0.155636	H	-1.234	4.006594	1.257732
C	-5.08788	1.134957	-0.26715	H	-0.04295	0.51436	-0.81336
C	-4.11313	0.156184	-0.60659	H	2.893111	3.217333	0.54774
C	-2.72621	0.462394	-0.51346	H	1.111198	4.634133	1.380682
C	2.596701	0.593017	-0.55455	H	-3.00443	3.666435	0.580163
C	2.301393	-0.50232	-1.4037	H	-5.36743	3.171727	0.396412
C	3.314652	-1.12766	-2.1612	H	3.015841	-1.91909	-2.84843
C	4.627485	-0.77695	-2.02881	H	5.400526	-1.23949	-2.64198
C	5.003594	0.115851	-1.00125	H	-1.8689	0.030584	-3.04789
C	3.994079	0.754243	-0.21562	H	0.523105	-0.40568	-3.49687
C	-1.73502	-0.55095	-0.9798	H	4.1351	-3.40744	0.797422
C	-1.26023	-0.47229	-2.29634	H	3.470086	-2.24187	1.989692
C	0.083514	-0.72888	-2.55328	H	2.82266	-3.9036	1.925387
C	0.923715	-1.06113	-1.48518	H	-2.22338	-0.2186	3.956432
C	0.32975	-1.62589	-0.33206	H	-3.24713	-1.53267	3.278987
C	-1.01894	-1.3545	-0.07961	H	-1.57882	-1.89175	3.808657
C	-1.66041	-1.77603	1.208404	H	-7.19319	1.582943	-0.10938
O	-2.10428	-2.88287	1.385758	H	-3.82569	-1.88985	-1.26416
O	-1.72624	-0.76862	2.079123	H	7.113284	-0.14746	-1.35035
C	1.14774	-2.20725	0.767627	H	3.706678	1.784794	1.671479
O	0.805007	-2.26445	1.927461	H	-7.92403	-0.66512	-0.85367
O	2.319242	-2.67734	0.320858	H	-6.22608	-2.39786	-1.42077
C	3.237137	-3.08448	1.328256	H	6.066179	2.073749	2.169228
C	-2.23074	-1.13315	3.360704	H	7.814386	1.193672	0.615375

Table S17. Cartesian coordinates of optimized *cis*-**3f'** [M06/6-31g(d) level of theory].

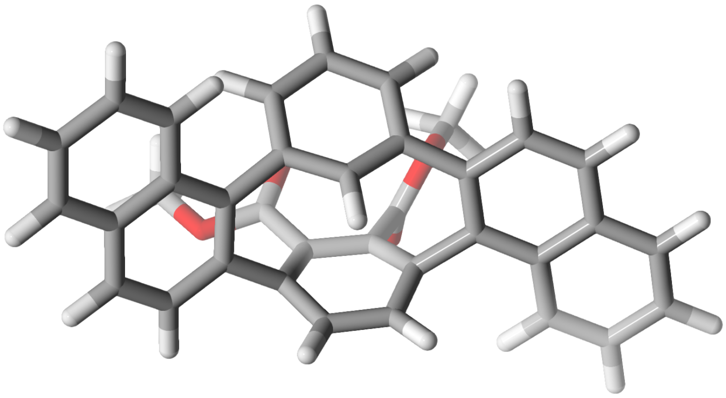

C	-0.5323	0.634203	2.581196	C	-6.20027	1.867123	-0.30688
C	-0.7346	1.188523	1.31366	C	-4.53748	0.169432	-1.77753
C	0.381411	1.365041	0.489327	C	6.326298	1.4018	-0.80213
C	1.626614	0.80755	0.802069	C	4.342932	1.959533	1.078392
C	1.789865	0.235003	2.068108	C	-6.69306	1.191284	-1.39353
C	0.72843	0.194396	2.965702	C	-5.85262	0.330293	-2.13267
C	-2.13407	1.399538	0.831799	C	5.613239	2.45205	1.241908
C	-3.00875	2.227527	1.575905	C	6.621032	2.165379	0.297289
C	-4.31981	2.392727	1.212561	H	-1.38893	0.480519	3.237555
C	-4.84909	1.720391	0.087934	H	0.251877	1.847902	-0.47462
C	-3.99554	0.863567	-0.66672	H	2.744076	-0.22436	2.328827
C	-2.63808	0.715228	-0.27923	H	0.867337	-0.25892	3.945503
C	2.691376	0.628245	-0.23553	H	-2.60924	2.752962	2.44317
C	2.42753	-0.11869	-1.38669	H	-4.97475	3.04761	1.787772
C	3.440126	-0.3557	-2.3413	H	3.193118	-0.94718	-3.22242
C	4.708546	0.120784	-2.15009	H	5.493167	-0.07723	-2.88061
C	5.024057	0.884684	-1.00308	H	-1.59427	1.327224	-2.6886
C	4.007477	1.149417	-0.03663	H	0.784136	0.902005	-3.20129
C	-1.65578	-0.0808	-1.06696	H	3.914945	-3.85854	-0.2453
C	-1.06855	0.513606	-2.18704	H	3.202187	-3.2352	1.282143
C	0.266496	0.270642	-2.47853	H	2.455515	-4.63162	0.467788
C	1.02075	-0.54258	-1.62963	H	-3.11025	-2.09027	3.230085
C	0.330217	-1.43881	-0.79601	H	-3.77383	-3.00906	1.834675
C	-1.02674	-1.21417	-0.53329	H	-2.19709	-3.4738	2.530283
C	-1.79727	-2.19141	0.303448	H	-6.84274	2.528751	0.274806
O	-2.06223	-3.29791	-0.09838	H	-3.89116	-0.50345	-2.34083
O	-2.19967	-1.69394	1.473708	H	7.090637	1.18251	-1.54822
C	1.03735	-2.44606	0.040019	H	3.569475	2.196462	1.806504
O	0.616187	-2.89453	1.082924	H	-7.73445	1.311655	-1.68674
O	2.214155	-2.8042	-0.49323	H	-6.25393	-0.21155	-2.98726
C	2.988919	-3.68866	0.307208	H	5.84442	3.073864	2.105161
C	-2.86427	-2.63183	2.31394	H	7.625973	2.558843	0.439482

Table S18. Cartesian coordinates of optimized **S1** [B3LYP/6-31g(d) level of theory].

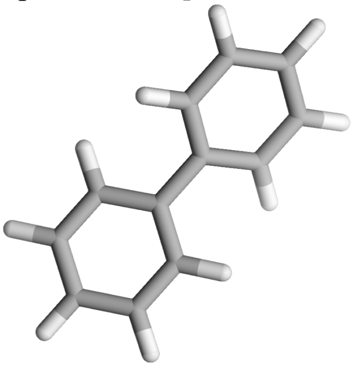

C	-11.166	-0.53602	0.032638	C	-9.16916	0.575249	-1.02011
C	-10.7803	-1.86589	-0.20713	H	-9.73913	-2.08805	-0.42434
C	-11.7067	-2.9054	-0.1376	H	-11.3829	-3.92702	-0.3192
C	-13.0409	-2.63889	0.174905	H	-13.7632	-3.44894	0.229707
C	-13.4395	-1.32291	0.416411	H	-14.4773	-1.10225	0.652196
C	-12.5126	-0.28393	0.345315	H	-12.8395	0.739369	0.508331
C	-10.1784	0.571542	-0.04229	H	-10.9818	1.650825	1.643895
C	-10.2268	1.645606	0.862607	H	-9.35345	3.501491	1.508337
C	-9.3008	2.68542	0.792274	H	-7.58124	3.484454	-0.23937
C	-8.30349	2.674404	-0.18456	H	-7.47512	1.598804	-1.86065
C	-8.24191	1.613918	-1.0904	H	-9.12843	-0.23111	-1.7472

Table S19. Cartesian coordinates of optimized **S2** [B3LYP/6-31g(d) level of theory].

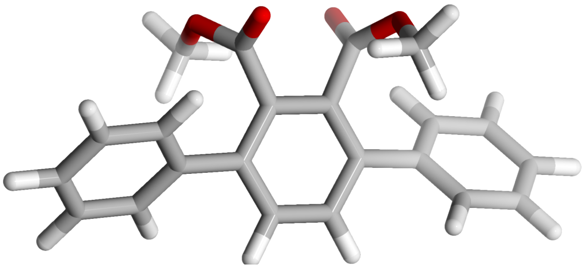

C	-8.78189	0.774466	-0.33251	C	-12.9178	-4.94818	0.844864
C	-8.27733	-0.48684	0.022651	C	-13.2698	-3.72659	1.420571
C	-9.11779	-1.57033	0.237997	C	-12.5037	-2.58608	1.180988
C	-10.5112	-1.45575	0.108348	C	-10.8187	2.191396	-0.93445
C	-11.0292	-0.19713	-0.25517	O	-11.2536	2.282334	-2.06426
C	-10.179	0.904167	-0.45818	O	-10.8955	3.238366	-0.10023
C	-7.83547	1.902548	-0.55715	C	-10.6561	3.05848	1.308788
C	-6.80118	2.139247	0.365748	C	-12.5199	0.059963	-0.32297
C	-5.8847	3.170053	0.163772	O	-13.0774	0.688827	0.553192
C	-5.98134	3.981296	-0.96833	O	-13.2261	-0.41961	-1.35704
C	-7.00033	3.753599	-1.89431	C	-12.5481	-0.90507	-2.53164
C	-7.91971	2.724239	-1.69392	H	-7.2027	-0.61602	0.112279
C	-11.3681	-2.64877	0.355891	H	-8.69479	-2.52681	0.530905
C	-11.0213	-3.88663	-0.21419	H	-6.72853	1.519552	1.255771
C	-11.7899	-5.02462	0.025578	H	-5.09808	3.341819	0.893665

H	-5.26766	4.785073	-1.12717	H	-12.7804	-1.64655	1.647899
H	-7.07997	4.375398	-2.78174	H	-10.9482	4.004502	1.766407
H	-8.69716	2.548877	-2.43015	H	-11.2788	2.24935	1.697386
H	-10.152	-3.94984	-0.86357	H	-9.59769	2.867549	1.505756
H	-11.5097	-5.9698	-0.43176	H	-13.3386	-1.06652	-3.26569
H	-13.5178	-5.83436	1.032751	H	-11.846	-0.15364	-2.90014
H	-14.142	-3.65832	2.064811	H	-12.0387	-1.85084	-2.32787

Table S20. Cartesian coordinates of optimized **S3** [B3LYP/6-31g(d) level of theory].

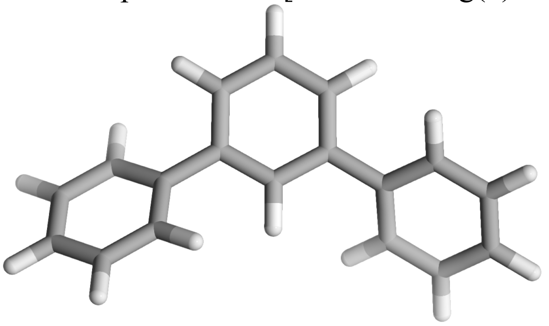

C	-8.7456	2.36401	-0.58662	C	-11.9017	-2.95159	1.501284
C	-8.19177	1.115798	-0.25774	C	-11.0578	-1.87375	1.237016
C	-9.06417	0.047394	-0.00257	H	-8.08989	3.199515	-0.81434
C	-10.4577	0.193861	-0.06738	H	-8.648	-0.9227	0.252907
C	-10.9802	1.454701	-0.39936	H	-12.0564	1.599261	-0.42978
C	-10.1278	2.526918	-0.65565	H	-10.5443	3.497764	-0.91136
C	-6.71903	0.927433	-0.18956	H	-6.73691	-1.02852	-1.09946
C	-6.11747	-0.25323	-0.65711	H	-4.2924	-1.3473	-0.96663
C	-4.73594	-0.4287	-0.59129	H	-2.84717	0.43728	-0.00645
C	-3.92404	0.573645	-0.05738	H	-3.88575	2.536789	0.835487
C	-4.50704	1.752803	0.410242	H	-6.33444	2.837668	0.735864
C	-5.8887	1.927087	0.34502	H	-12.7693	-0.46929	-1.33967
C	-11.3568	-0.9551	0.216649	H	-14.2751	-2.36649	-0.86036
C	-12.5313	-1.15259	-0.5291	H	-13.7233	-3.97434	0.957828
C	-13.3762	-2.22957	-0.26477	H	-11.6538	-3.64469	2.301043
C	-13.0651	-3.13458	0.751678	H	-10.1693	-1.72571	1.844525

Table S21. Cartesian coordinates of optimized **S4** [B3LYP/6-31g(d) level of theory].

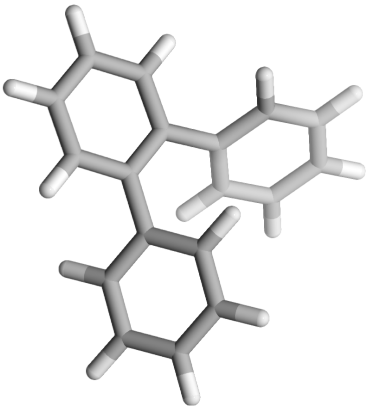

C	-7.11669	1.939063	-0.59163	C	-10.2078	-3.18167	2.002356
C	-6.58582	0.744068	-0.10817	C	-9.47647	-2.036	1.686819
C	-7.4443	-0.29579	0.240342	H	-6.46305	2.761669	-0.86918
C	-8.83956	-0.17712	0.12296	H	-5.51099	0.616093	-0.01162
C	-9.38016	1.044887	-0.34974	H	-7.03189	-1.24044	0.584091
C	-8.4977	2.078915	-0.70564	H	-8.91559	3.021357	-1.04882
C	-10.8445	1.313245	-0.45322	H	-11.3087	0.70668	1.563026
C	-11.7073	1.105946	0.635531	H	-13.7116	1.253235	1.397853
C	-13.0629	1.415886	0.54102	H	-14.6442	2.175881	-0.71707
C	-13.5865	1.936953	-0.64436	H	-13.1377	2.550519	-2.66307
C	-12.7412	2.148845	-1.73408	H	-10.7314	2.00091	-2.49257
C	-11.3832	1.842826	-1.63723	H	-10.7741	-1.39148	-1.38546
C	-9.67064	-1.368	0.466729	H	-12.0658	-3.42287	-0.83356
C	-10.6161	-1.88745	-0.43277	H	-11.714	-4.57883	1.342171
C	-11.3435	-3.03451	-0.12022	H	-10.0465	-3.67783	2.955975
C	-11.1442	-3.68587	1.099116	H	-8.75531	-1.64226	2.398451

Table S22. Cartesian coordinates of optimized **S5** [B3LYP/6-31g(d) level of theory].

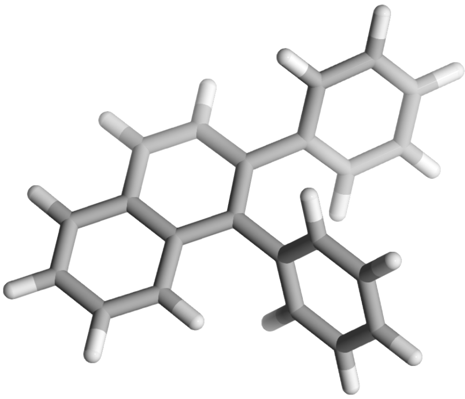

C	-9.06725	-1.15139	2.959337	C	-11.3121	-0.70924	-0.03503
C	-7.84415	-0.55968	2.554958	C	-11.2969	-1.28978	-1.31337
C	-7.75374	0.021993	1.246958	C	-12.4712	-1.41701	-2.05392
C	-8.89188	-0.02296	0.367193	C	-13.686	-0.96592	-1.53386
C	-10.0808	-0.61011	0.804231	C	-13.7174	-0.39006	-0.26316
C	-10.1473	-1.16508	2.115038	C	-12.5428	-0.26841	0.479522

C	-8.79032	0.602448	-0.9892
C	-7.94555	0.061948	-1.97219
C	-7.85175	0.64719	-3.2355
C	-8.59727	1.788464	-3.53653
C	-9.43649	2.3386	-2.56611
C	-9.53189	1.750605	-1.30478
C	-6.71837	-0.5212	3.420071
C	-5.54521	0.081984	3.030176
C	-5.45673	0.681743	1.752228
C	-6.52882	0.654695	0.88778
H	-9.13607	-1.59689	3.949026
H	-11.0753	-1.6345	2.4283
H	-10.3596	-1.64974	-1.72489
H	-12.4367	-1.8743	-3.03938
H	-14.6	-1.06406	-2.11356
H	-14.6564	-0.03297	0.151881
H	-12.5731	0.187555	1.465886
H	-7.36411	-0.82733	-1.74244
H	-7.19665	0.209781	-3.98458
H	-8.52438	2.245638	-4.51976
H	-10.0192	3.228297	-2.79029
H	-10.1889	2.180366	-0.55415
H	-6.80591	-0.97536	4.404475
H	-4.69122	0.107055	3.701676
H	-4.53507	1.172615	1.450952
H	-6.44496	1.128195	-0.0836

Table S23. Uncorrected and thermally-corrected (298K) energies of stationary points (Hartree) calculated by the M06/6-31G(d) level of theory for estimation of rotational barriers.

compound	Sum of electronic and zero-point Energies	Sum of electronic and thermal Energies	Sum of electronic and thermal Enthalpies	Sum of electronic and thermal Free Energies	imaginary frequency (cm^{-1})
trans-**3a'**	-1378.564508	-1378.538931	-1378.537987	-1378.619039	
cis-**3a'**	-1378.565886	-1378.540154	-1378.539210	-1378.620783	
trans-**3c'**	-1532.047367	-1532.018866	-1532.017921	-1532.105548	
cis-**3c'**	-1532.046705	-1532.018245	-1532.017301	-1532.104564	
trans-**3e'**	-1532.043567	-1532.015284	-1532.014340	-1532.101466	
cis-**3e'**	-1532.044930	-1532.016504	-1532.015560	-1532.102515	
trans-**3f'**	-1685.525868	-1685.494805	-1685.493861	-1685.586960	
cis-**3f'**	-1685.525669	-1685.494532	-1685.493587	-1685.586648	
TS-**3a'**	-1378.541283	-1378.516154	-1378.515210	-1378.596663	-99.3637
TS-**3c'**	-1532.021719	-1531.994000	-1531.993056	-1532.078883	-104.0359
TS-**3e'**	-1532.005712	-1531.978176	-1531.977232	-1532.062108	-90.8004
TS-**3f'**	-1685.486358	-1685.456149	-1685.455205	-1685.546111	-85.8319

Table S24. Uncorrected and thermally-corrected (298K) energies of stationary points (Hartree) calculated by the B3LYP/6-31G(d) level of theory for estimation of strain energies.

compound	Sum of electronic and zero-point Energies	Sum of electronic and thermal Energies	Sum of electronic and thermal Enthalpies	Sum of electronic and thermal Free Energies
trans-**3a'**	-1379.494275	-1379.468392	-1379.467448	-1379.550191
cis-**3a'**	-1379.494656	-1379.468696	-1379.467752	-1379.551058
trans-**3c'**	-1533.090720	-1533.062140	-1533.061196	-1533.149623
cis-**3c'**	-1533.090765	-1533.062094	-1533.061150	-1533.150258
trans-**3e'**	-1533.087607	-1533.059008	-1533.058064	-1533.146871
cis-**3e'**	-1533.088472	-1533.059832	-1533.058888	-1533.147713
trans-**3f'**	-1686.684098	-1686.652788	-1686.651844	-1686.746335
cis-**3f'**	-1686.684648	-1686.653286	-1686.652342	-1686.747146
**S1**	-463.124101	-463.115231	-463.114286	-463.158662
**S2**	-1149.732498	-1149.709658	-1149.708714	-1149.785427
**S3**	-694.100347	-694.086805	-694.085861	-694.141811
**S4**	-694.094272	-694.080673	-694.079728	-694.135548
**S5**	-847.687601	-847.671274	-847.670330	-847.732438

5. Determination of Absolute Configuration of (+)-3c

All calculations were carried out using the Gaussian 16 program,[4]. Conformational searches were carried out via ETKDG searching using molecular mechanism with MMFF force field in python 3. The results showed four lowest energy conformers, (*R*)-**3c-1**, (*R*)-**3c-2**, (*R*)-**3c-3**, and (*R*)-**3c-4**, each differing in the orientation of the ester carbonyl groups. The hybrid density functional method based on B3LYP[5,6] with a 6-31g(d) basis set was used for geometry optimizations of the conformers. Harmonic vibration frequency calculations at the same level were performed to verify all stationary points as local minima (with no imaginary frequency). Cartesian coordinates of optimized structures are listed in Tables S28–S31. Gibbs free energy at 298.15 K and 1 atm were calculated based on B3LYP, CAM-B3LYP, ωb97xd or M06[7] with a 6-311++G(2d, 2p) basis set and estimated from the gas-phase studies. The results are shown in Table S25. Specific rotations at 589 nm were calculated based on B3LYP, CAM-B3LYP, ωb97xd or M06 with a 6-311++G(2d, 2p) basis set. The calculated results for each conformer and the averaged values according to the Boltzmann distribution theory were summarized in Table S26 and S27. The solvation effect was examined by performing single-point self-consistent reaction field (SCRF)[11] calculations based on the polarizable continuum model (PCM) for gas-phase optimized structures.

Table S25. Gibbs free energies of (*R*)-**3c** conformers and their relative Gibbs free energies (Δ*G*).

	Conformer	Sum of electronic and thermal Free Energies (Hartree)	Δ*G* (kcal/mol)
B3LYP/6-311++G(2d,2p)	(*R*)-**3c-1**	-1572.893535	0.000000
	(*R*)-**3c-2**	-1572.891807	1.084337
	(*R*)-**3c-3**	-1572.891756	1.116340
	(*R*)-**3c-4**	-1572.891303	1.400602
CAM-B3LYP/6-311++G(2d,2p)	(*R*)-**3c-1**	-1572.065581	0.000000
	(*R*)-**3c-2**	-1572.063957	1.019076
	(*R*)-**3c-3**	-1572.064047	0.962600
	(*R*)-**3c-4**	-1572.063163	1.517319
ωb97xd/6-311++G(2d,2p)	(*R*)-**3c-1**	-1572.363694	0.000000
	(*R*)-**3c-2**	-1572.362562	0.710341
	(*R*)-**3c-3**	-1572.362563	0.709714
	(*R*)-**3c-4**	-1572.361926	1.109438
M06/6-311++G(2d,2p)	(*R*)-**3c-1**	-1571.770765	0.000000
	(*R*)-**3c-2**	-1571.769303	0.917420
	(*R*)-**3c-3**	-1571.769445	0.828313
	(*R*)-**3c-4**	-1571.767845	1.832329

Table S26. Relative Gibbs free energies (ΔG), the Boltzmann distributions, and calculated specific rotations at 589 nm for each conformer of (*R*)-**3c**.

	Conformer	ΔG (kcal/mol)	Boltzmann Distribution	Specific Rotation (589 nm)		
				$CHCl_3$	CH_2Cl_2	Acetone
B3LYP/ 6-311++G(2d,2p)	(*R*)-**3c-1**	0.000000	0.718104	277.72	278.62	281.12
	(*R*)-**3c-2**	1.084337	0.111535	191.98	194.08	197.02
	(*R*)-**3c-3**	1.116340	0.105570	-65.81	-68.39	-67.62
	(*R*)-**3c-4**	1.400602	0.064791	7.89	10.83	15.31
CAM-B3LYP/ 6-311++G(2d,2p)	(*R*)-**3c-1**	0.000000	0.694921	229.02	229.80	232.72
	(*R*)-**3c-2**	1.019076	0.120735	161.05	162.14	164.70
	(*R*)-**3c-3**	0.962600	0.133033	-18.12	-19.92	-19.33
	(*R*)-**3c-4**	1.517319	0.051311	51.34	53.07	56.06
ωb97xd/ 6-311++G(2d,2p)	(*R*)-**3c-1**	0.000000	0.574857	225.15	225.94	228.86
	(*R*)-**3c-2**	0.710341	0.169721	154.92	155.87	158.31
	(*R*)-**3c-3**	0.709714	0.169904	-11.33	-13.16	-12.71
	(*R*)-**3c-4**	1.109438	0.085519	48.53	50.24	53.16
M06/ 6-311++G(2d,2p)	(*R*)-**3c-1**	0.000000	0.670709	291.05	291.62	293.80
	(*R*)-**3c-2**	0.917420	0.138757	205.16	207.27	210.10
	(*R*)-**3c-3**	0.828313	0.161703	-63.20	-66.29	-65.75
	(*R*)-**3c-4**	1.832329	0.028830	29.81	32.57	36.71

Table S27. Averaged specific rotations according to the Boltzmann distribution theory of (*R*)-**3c** and experimental values in each solvent.

	$CHCl_3$	CH_2Cl_2	Acetone
B3LYP/ 6-311++G(2d,2p)	214	215	218
CAM-B3LYP/ 6-311++G(2d,2p)	179	179	182
ωb97xd/ 6-311++G(2d,2p)	158	158	161
M06/ 6-311++G(2d,2p)	214	215	217
Experimental Value	138	120	324

Table S28. Cartesian coordinates of optimized (*R*)-**3c-1** [B3LYP/6-31g(d) level of theory].

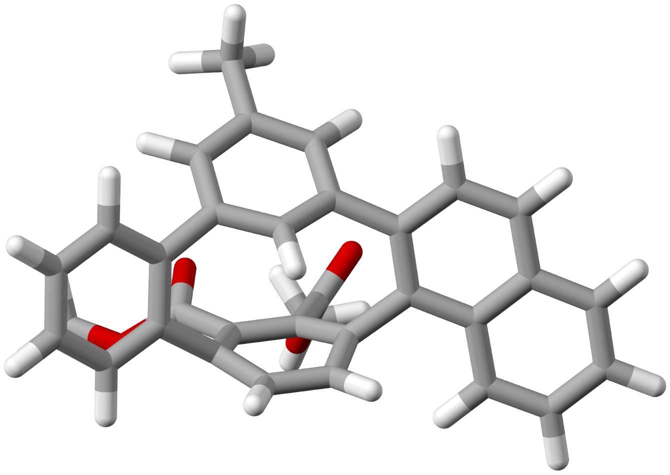

O	1.43670	1.26998	1.90341	C	-2.87240	-0.28787	-1.61368
C	0.81698	1.81126	1.01621	C	-3.92340	-0.01023	-2.49225
O	0.59591	3.14220	0.95037	C	-5.09061	-0.77377	-2.48223
C	0.98287	3.87815	2.12147	C	-5.21421	-1.83392	-1.58441
C	0.27053	1.13877	-0.21483	C	-4.16988	-2.12864	-0.70841
C	-1.09968	1.18508	-0.52726	C	-0.55425	-1.83749	-0.11782
C	-2.05156	1.80153	0.44988	H	0.78190	4.92441	1.88825
O	-3.15722	2.27108	-0.16729	H	0.38591	3.55340	2.97795
C	-4.15154	2.83205	0.70293	H	2.04307	3.72764	2.33987
O	-1.87089	1.89370	1.64765	H	-4.49541	2.08621	1.42459
C	-1.57357	0.45896	-1.64415	H	-3.74593	3.69048	1.24499
C	-0.64271	0.11422	-2.63229	H	-4.96883	3.13974	0.05017
C	0.71489	0.07039	-2.31890	H	-0.99220	-0.34856	-3.55181
C	1.13971	0.36754	-1.01564	H	1.40664	-0.41264	-3.00418
C	2.26766	-0.43020	-0.43514	H	6.77503	-1.46233	-0.20516
C	3.63274	-0.17880	-0.76552	H	7.39604	0.46141	-1.63276
C	4.65265	-1.04916	-0.25835	H	5.62934	1.99484	-2.49573
C	6.00959	-0.79392	-0.59277	H	3.26014	1.59959	-1.94593
C	6.35409	0.27657	-1.38581	H	5.04380	-2.80211	0.94881
C	5.34999	1.14572	-1.87767	H	2.67574	-3.20062	1.50505
C	4.02553	0.92466	-1.57412	H	0.97691	-1.74505	2.90502
C	4.27240	-2.13811	0.56577	H	-2.43266	-1.44146	4.30298
C	2.95190	-2.35796	0.87732	H	-0.70214	-1.43435	4.68163
C	1.91720	-1.52075	0.38004	H	-1.54561	-2.96523	4.42207
C	0.49930	-1.77812	0.80562	H	-3.20378	-1.64717	1.99109
C	0.17913	-1.81595	2.17051	H	-3.82657	0.82868	-3.17614
C	-1.15129	-1.85241	2.60549	H	-5.89894	-0.53835	-3.16936
C	-1.47508	-1.92362	4.07979	H	-6.11816	-2.43709	-1.57001
C	-2.17505	-1.75388	1.65421	H	-4.26104	-2.96719	-0.02304
C	-1.89352	-1.71207	0.28095	H	-0.32524	-1.88370	-1.17132
C	-2.98073	-1.38406	-0.70397				

Table S29. Cartesian coordinates of optimized (*R*)-**3c-2** [B3LYP/6-31g(d) level of theory].

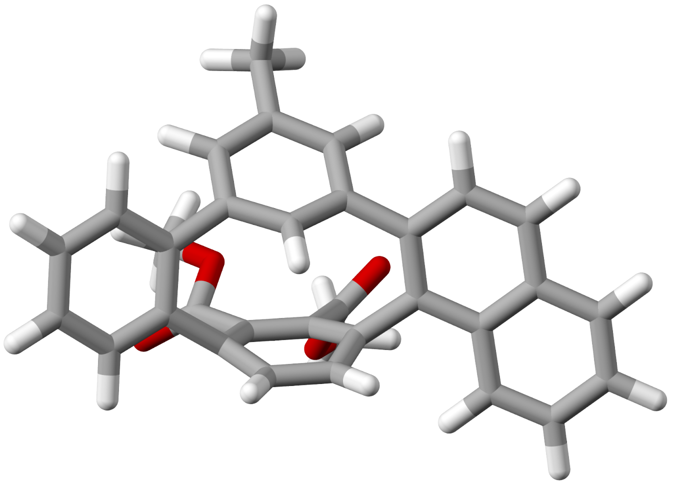

O	1.49464	2.07178	0.83229	C	-2.88711	-1.06664	-1.39647
C	0.81318	2.05662	-0.16823	C	-3.91758	-1.24763	-2.32227
O	0.51119	3.16450	-0.88587	C	-5.11494	-1.86127	-1.95441
C	0.98365	4.39791	-0.32313	C	-5.28926	-2.30478	-0.64399
C	0.26294	0.83604	-0.84741	C	-4.26807	-2.13400	0.29031
C	-1.09637	0.72065	-1.18505	C	-0.65095	-1.63401	0.77036
C	-2.11758	1.75006	-0.80417	H	2.07178	4.38173	-0.22040
O	-1.88361	2.29515	0.41037	H	0.67445	5.17513	-1.02273
C	-2.81392	3.31271	0.81021	H	0.53713	4.56471	0.66122
O	-3.06804	2.04759	-1.49600	H	-3.82944	2.90996	0.84366
C	-1.55938	-0.48049	-1.76736	H	-2.49134	3.62785	1.80296
C	-0.61536	-1.30692	-2.38777	H	-2.78896	4.15244	0.11012
C	0.73210	-1.19267	-2.04756	H	-0.95465	-2.18079	-2.93826
C	1.13029	-0.25493	-1.08464	H	1.42704	-1.97388	-2.34445
C	2.21817	-0.63802	-0.12802	H	6.68664	-1.43834	0.78222
C	3.59833	-0.61296	-0.48906	H	7.40013	-0.56487	-1.42061
C	4.57905	-1.09449	0.43882	H	5.70143	0.29781	-3.02915
C	5.95121	-1.06767	0.07184	H	3.30801	0.27548	-2.44380
C	6.34712	-0.58093	-1.15286	H	4.88737	-1.95169	2.40187
C	5.38166	-0.09315	-2.06691	H	2.49492	-1.97650	2.99276
C	4.04381	-0.10732	-1.74263	H	0.79974	-0.02705	3.37922
C	4.14594	-1.58142	1.69772	H	-0.94203	1.16771	4.68336
C	2.81171	-1.59184	2.02730	H	-2.66193	0.91357	4.33411
C	1.81474	-1.13226	1.12503	H	-1.74771	-0.30738	5.22327
C	0.37942	-1.11759	1.56970	H	-3.35302	-0.39049	2.39700
C	0.02108	-0.45350	2.75187	H	-3.78153	-0.88055	-3.33557
C	-1.32226	-0.26139	3.09928	H	-5.90771	-1.98585	-2.68698
C	-1.68775	0.41709	4.39971	H	-6.21680	-2.78767	-0.34797
C	-2.31713	-0.65376	2.19455	H	-4.40193	-2.49440	1.30692
C	-1.99762	-1.31500	0.99903	H	-0.39395	-2.21722	-0.09967
C	-3.05097	-1.52672	-0.05306				

Table S30. Cartesian coordinates of optimized (*R*)-**3c-3** [B3LYP/6-31g(d) level of theory].

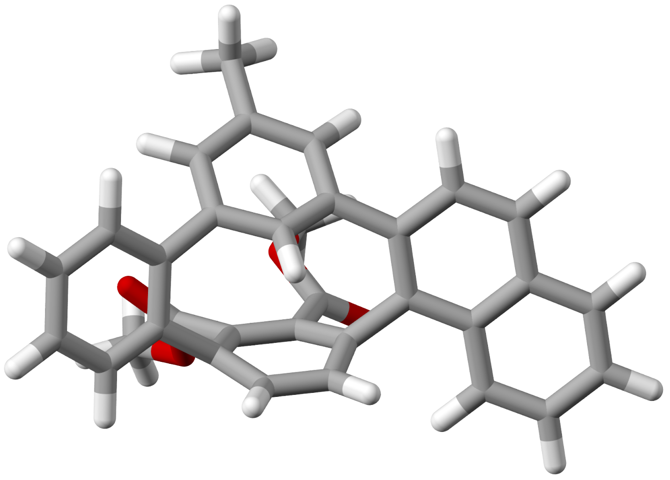

O	1.89095	2.49934	-0.44708	C	-2.91142	-1.14603	-1.27678
C	0.81260	2.01078	-0.18440	C	-3.95013	-1.38981	-2.17712
O	0.00548	2.47323	0.79561	C	-5.12041	-2.03152	-1.76716
C	0.48701	3.63988	1.48008	C	-5.25248	-2.44334	-0.44281
C	0.24656	0.81407	-0.88883	C	-4.21662	-2.21522	0.46491
C	-1.12212	0.67215	-1.17261	C	-0.61148	-1.60680	0.84052
C	-2.11253	1.75829	-0.87154	H	1.45529	3.43775	1.94495
O	-1.71686	2.92294	-1.43778	H	0.59636	4.47576	0.78349
C	-2.58911	4.03858	-1.20233	H	-0.26498	3.86393	2.23720
O	-3.15838	1.62477	-0.27601	H	-2.65883	4.25017	-0.13160
C	-1.59924	-0.55523	-1.68744	H	-2.13832	4.87832	-1.73218
C	-0.66792	-1.38989	-2.31965	H	-3.59068	3.83178	-1.58833
C	0.69087	-1.25220	-2.03757	H	-1.02019	-2.28257	-2.83022
C	1.11405	-0.27432	-1.12885	H	1.37979	-2.04020	-2.33080
C	2.22264	-0.61015	-0.17521	H	6.72132	-1.32671	0.66281
C	3.59613	-0.57921	-0.56482	H	7.37201	-0.53198	-1.58927
C	4.60290	-1.01343	0.35726	H	5.62854	0.27156	-3.17948
C	5.96543	-0.99020	-0.04308	H	3.25030	0.26138	-2.52943
C	6.32604	-0.54670	-1.29495	H	4.96666	-1.77971	2.34905
C	5.33482	-0.09556	-2.19981	H	2.59073	-1.79581	3.00013
C	4.00534	-0.10797	-1.84294	H	0.87365	0.08318	3.37650
C	4.20489	-1.44676	1.64787	H	-0.78485	1.19560	4.75783
C	2.88046	-1.45127	2.01130	H	-1.79734	-0.16983	5.23551
C	1.85404	-1.04861	1.11092	H	-2.50011	1.15833	4.30928
C	0.42988	-1.04815	1.59458	H	-3.29304	-0.33062	2.47517
C	0.08726	-0.36027	2.76951	H	-3.84330	-1.05927	-3.20715
C	-1.24980	-0.16974	3.13912	H	-5.92146	-2.20633	-2.48027
C	-1.60010	0.54443	4.42455	H	-6.15704	-2.94791	-0.11378
C	-2.26018	-0.59882	2.26788	H	-4.31819	-2.55121	1.49353
C	-1.95773	-1.30196	1.09384	H	-0.36598	-2.21485	-0.01567
C	-3.03129	-1.57717	0.07971				

Table S31. Cartesian coordinates of optimized (*R*)-**3c-4** [B3LYP/6-31g(d) level of theory].

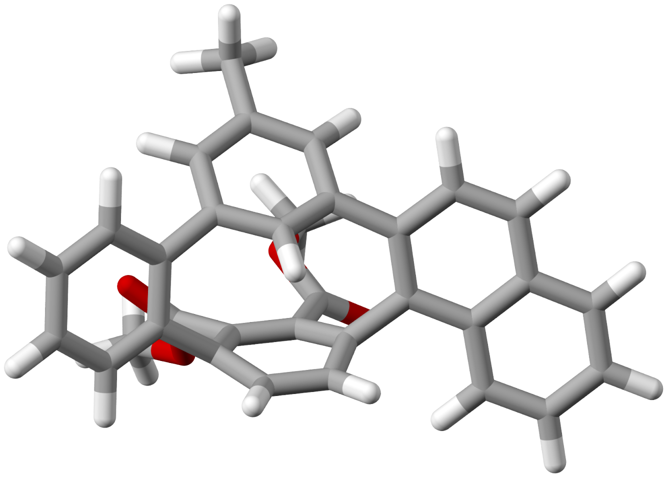

O	-0.22658	0.81825	3.22861	C	2.95275	-1.67846	-0.29525
C	-0.71255	0.62415	2.13120	C	4.00502	-2.56207	-0.04167
O	-1.77674	1.30464	1.66711	C	5.21367	-2.45819	-0.73066
C	-2.32830	2.27364	2.57297	C	5.37664	-1.46004	-1.69029
C	-0.22277	-0.43959	1.19847	C	4.33490	-0.57131	-1.95518
C	1.14048	-0.77936	1.22494	C	0.69397	-0.00832	-1.73971
C	2.14974	-0.02036	2.04669	H	-3.18358	2.70260	2.05052
O	2.15179	1.29935	1.77737	H	-2.64439	1.79389	3.50292
C	3.01703	2.08564	2.61093	H	-1.58899	3.04501	2.80411
O	2.90807	-0.55722	2.82372	H	2.68381	2.02997	3.65073
C	1.61837	-1.79623	0.37400	H	2.93416	3.10636	2.23587
C	0.68797	-2.71970	-0.11693	H	4.04819	1.73020	2.54179
C	-0.66440	-2.38114	-0.15192	H	1.03815	-3.58367	-0.67634
C	-1.08083	-1.12013	0.29813	H	-1.35067	-2.98509	-0.73986
C	-2.17360	-0.44130	-0.47278	H	-6.64517	-0.04926	-1.61425
C	-3.54970	-0.78081	-0.30327	H	-7.33804	-1.67007	0.12291
C	-4.53745	-0.17030	-1.14344	H	-5.62766	-2.71405	1.60692
C	-5.90448	-0.51487	-0.96808	H	-3.24266	-2.15243	1.34515
C	-6.28894	-1.41563	-0.00127	H	-4.86251	1.22467	-2.76673
C	-5.31690	-2.01021	0.83951	H	-2.47847	1.78913	-3.03088
C	-3.98364	-1.69941	0.69320	H	-0.81089	3.01974	-1.49263
C	-4.11637	0.76429	-2.12328	H	0.87382	4.76203	-1.06473
C	-2.78706	1.08108	-2.26668	H	1.77334	4.56309	-2.57065
C	-1.78175	0.48317	-1.45759	H	2.60010	4.36107	-1.02415
C	-0.35418	0.91729	-1.63667	H	3.35827	2.05153	-1.35936
C	-0.01944	2.27863	-1.57900	H	3.87526	-3.32480	0.72087
C	1.31670	2.69934	-1.56468	H	6.02259	-3.15054	-0.51427
C	1.65834	4.17186	-1.55063	H	6.31204	-1.37320	-2.23674
C	2.32896	1.73295	-1.50672	H	4.46255	0.19613	-2.71411
C	2.03456	0.36243	-1.55884	H	0.45512	-1.05021	-1.87634
C	3.10720	-0.65408	-1.28134				

6. References

[1] Pinardi, A. L.; Otero-Irurueta, G.; Palacio, I.; Martinez, J. I.; Sanchez-Sanchez, C.; Tello, M.; Rogero, C.; Cossaro, A.; Preobrajenski, A.; Gómez-Lor, B.; Jancarik, A.; Stará, I. G.; Starý, I.; Lopez, M. F.; Méndez, J.; Martin-Gago, J. A. Tailored Formation of *N*-Doped Nanoarchitectures by Diffusion-Controlled on-Surface (Cyclo)-Dehydrogenation of Heteroaromatics. *ACS Nano* **2013**, *7*, 3676–3684.

[2] Nishibe, S.; Kishi, T.; Ito, M.; Shibata, T. Synthesis of Dithia[5]helicenes and Enantioselective Synthesis of Helically Chiral Thia[6]helicenes via Rh-Catalyzed Intramolecular [2 + 2 + 2] Cycloaddition of Triynes. *J. Org. Chem.* **2023**, *88*, 7703–7711.

[3] Weimar, M.; Correa da Costa, R.; Lee, F.-H.; Fuchter, M. J. A Scalable and Expedient Route to 1-Aza[6]helicene Derivatives and Its Subsequent Application to a Chiral-Relay Asymmetric Strategy. *Org. Lett.* **2013**, *15*, 1706–1709.

[4] (a) *Gaussian 16, Revision A.03*, Frisch, M. J.; Trucks, G. W.; Schlegel, H. B.; Scuseria, G. E.; Robb, M. A.; Cheeseman, J. R.; Scalmani, G.; Barone, V.; Petersson, G. A.; Nakatsuji, H.; Li, X.; Caricato, M.; Marenich, A. V.; Bloino, J.; Janesko, B. G.; Gomperts, R.; Mennucci, B.; Hratchian, H. P.; Ortiz, J. V.; Izmaylov, A. F.; Sonnenberg, J. L.; Williams-Young, D.; Ding, F.; Lipparini, F.; Egidi, F.; Goings, J.; Peng, B.; Petrone, A.; Henderson, T.; Ranasinghe, D.; Zakrzewski, V. G.; Gao, J.; Rega, N.; Zheng, G.; Liang, W.; Hada, M.; Ehara, M.; Toyota, K.; Fukuda, R.; Hasegawa, J.; Ishida, M.; Nakajima, T.; Honda, Y.; Kitao, O.; Nakai, H.; Vreven, T.; Throssell, K.; Montgomery, J. A.; Jr., Peralta, J. E.; Ogliaro, F.; Bearpark, M. J.; Heyd, J. J.; Brothers, E. N.; Kudin, K. N.; Staroverov, V. N.; Keith, T. A.; Kobayashi, R.; Normand, J.; Raghavachari, K.; Rendell, A. P.; Burant, J. C.; Iyengar, S. S.; Tomasi, J.; Cossi, M.; Millam, J. M.; Klene, M.; Adamo, C.; Cammi, R.; Ochterski, J. W.; Martin, R. L.; Morokuma, K.; Farkas, O.; Foresman, J. B.; Fox, D. J. Gaussian, Inc., Wallingford CT, 2016. (b) *Gaussian 16, Revision C.02*, Frisch, M. J.; Trucks, G. W.; Schlegel, H. B.; Scuseria, G. E.; Robb, M. A.; Cheeseman, J. R.; Scalmani, G.; Barone, V.; Petersson, G. A.; Nakatsuji, H.; Li, X.; Caricato, M.; Marenich, A. V.; Bloino, J.; Janesko, B. G.; Gomperts, R.; Mennucci, B.; Hratchian, H. P.; Ortiz, J. V.; Izmaylov, A. F.; Sonnenberg, J. L.; Williams-Young, D.; Ding, F.; Lipparini, F.; Egidi, F.; Goings, J.; Peng, B.; Petrone, A.; Henderson, T.; Ranasinghe, D.; Zakrzewski, V. G.; Gao, J.; Rega, N.; Zheng, G.; Liang, W.; Hada, M.; Ehara, M.; Toyota, K.; Fukuda, R.; Hasegawa, J.; Ishida, M.; Nakajima, T.; Honda, Y.; Kitao, O.; Nakai, H.; Vreven, T.; Throssell, K.; Montgomery, J. A., Jr.; Peralta, J. E.; Ogliaro, F.; Bearpark, M. J.; Heyd, J. J.; Brothers, E. N.; Kudin, K. N.; Staroverov, V. N.; Keith, T. A.; Kobayashi, R.; Normand, J.; Raghavachari, K.; Rendell, A. P.; Burant, J. C.; Iyengar, S. S.; Tomasi, J.; Cossi, M.; Millam, J. M.; Klene, M.; Adamo, C.; Cammi, R.; Ochterski, J. W.; Martin, R. L.; Morokuma, K.; Farkas, O.; Foresman, J. B.; Fox, D. J. Gaussian, Inc., Wallingford CT, 2016.

[5] Becke, A. D. Density-Functional Thermochemistry. III. The Role of Exact Exchange. *J. Chem. Phys.* **1993**, *98*, 5648–5652.

[6] Lee, C.; Yang, W.; Parr, R. G. Development of the Colle-Salvetti Correlation-Energy Formula into a Functional of the Electron Density. *Phys. Rev. B* **1988**, *37*, 785–789.

[7] Zhao, Y.; Truhlar, D. G. The M06 Suite of Density Functionals for Main Group Thermochemistry, Thermochemical Kinetics, Noncovalent Interactions, Excited States, and Transition Elements: Two New Functionals and Systematic Testing of Four M06-Class Functionals and 12 Other Functionals. *Theor. Chem. Acc.* **2008**, *120*, 215–241.

[8] Segawa, Y.; Yagi, A.; Ito, H.; Itami, K. A Theoretical Study on the Strain Energy of Carbon Nanobelts. *Org. Lett.* **2016**, *18*, 1430–1433.

[9] Colwell, C. E.; Price, T. W.; Stauch, T.; Jasti, R. Strain Visualization for Strained Macrocycles. *Chem. Sci.* **2020**, *11*, 3923–3930.

[10] Humphrey, W.; Dalke, A.; Schulten, K. VMD: Visual Molecular Dynamics. *J. Mol. Graph.* **1996**, *14*, 33–38.

[11] Tomasi, J.; Persico, M. Molecular Interactions in Solution: An Over-view of Methods Based on Continuous Distributions of the Solvent. *Chem. Rev.* **1994**, *94*, 2027–2094.

7. Chiral HPLC Charts

***o*,*m*,*o*,*p*-Tetraphenylene 3c**
CHIRALPAK IF-3, *n*-hexane/2-propanol = 93:7, 1.0 mL min^{-1}

(±)-**3c**

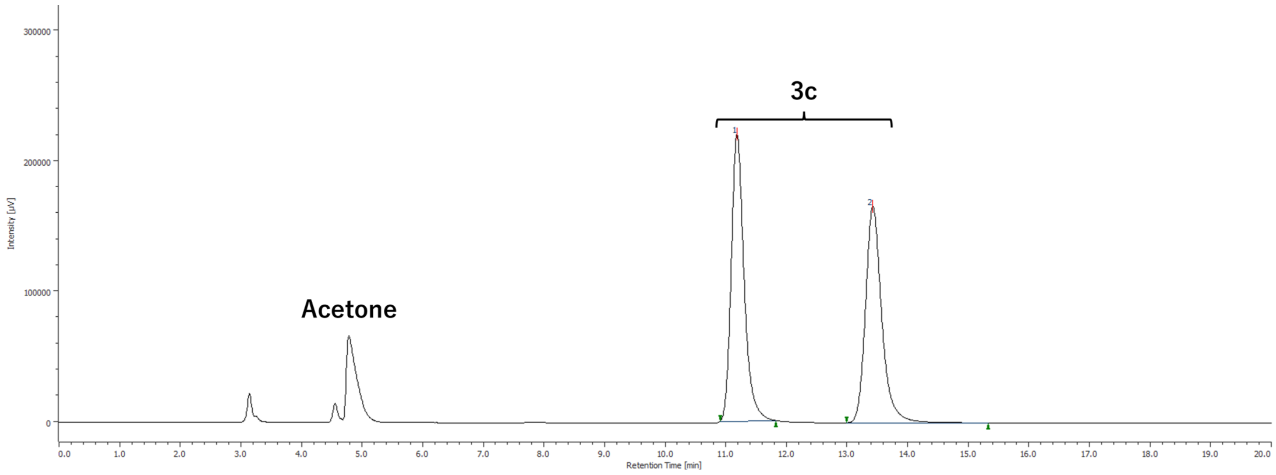

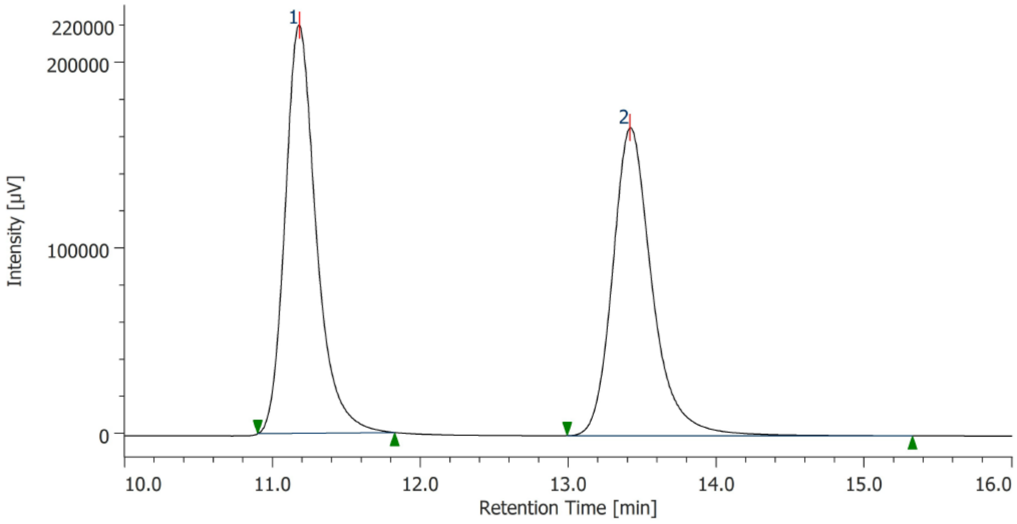

Peak No.	Retention Time (min)	Area (%)
1	11.183	51.016
2	13.417	48.984

(+)-**3c** using (*R*)-H_8-BINAP

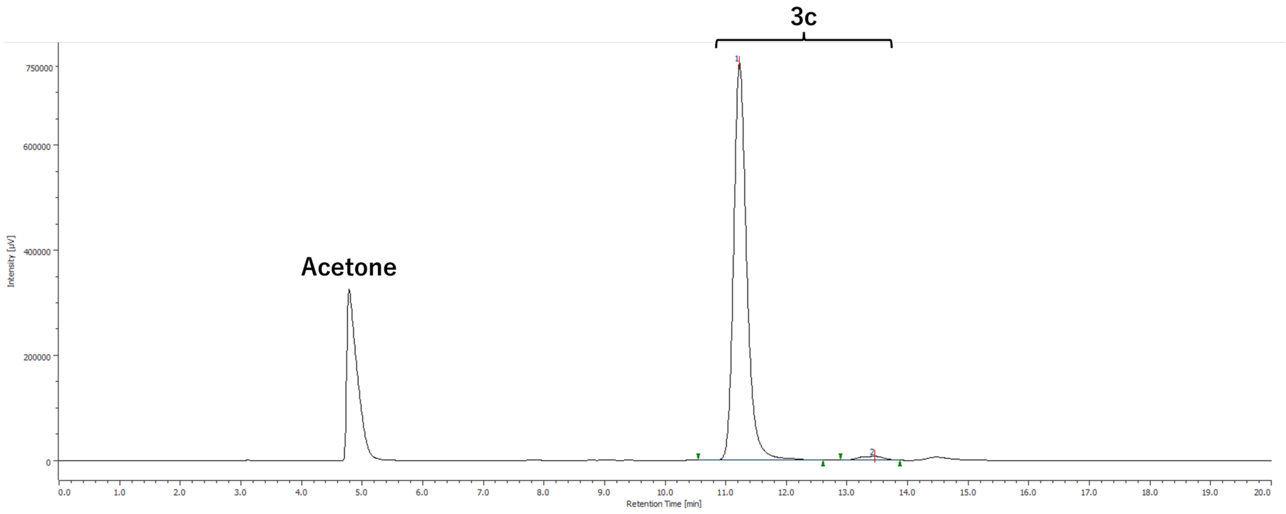

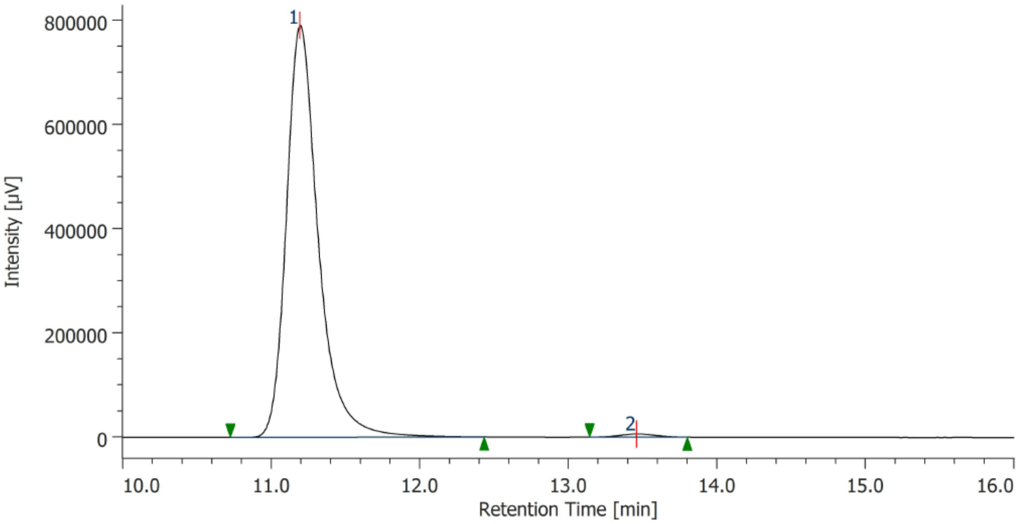

Peak No.	Retention Time (min)	Area (%)
1	11.192	99.154
2	13.458	0.846

***o*,*m*,*o*,*p*-Tetraphenylene 3d**
CHIRALPAK IF-3, *n*-hexane/2-propanol = 93:7, 1.0 mL min^{-1}

(±)-**3d**, (±)-**4d**

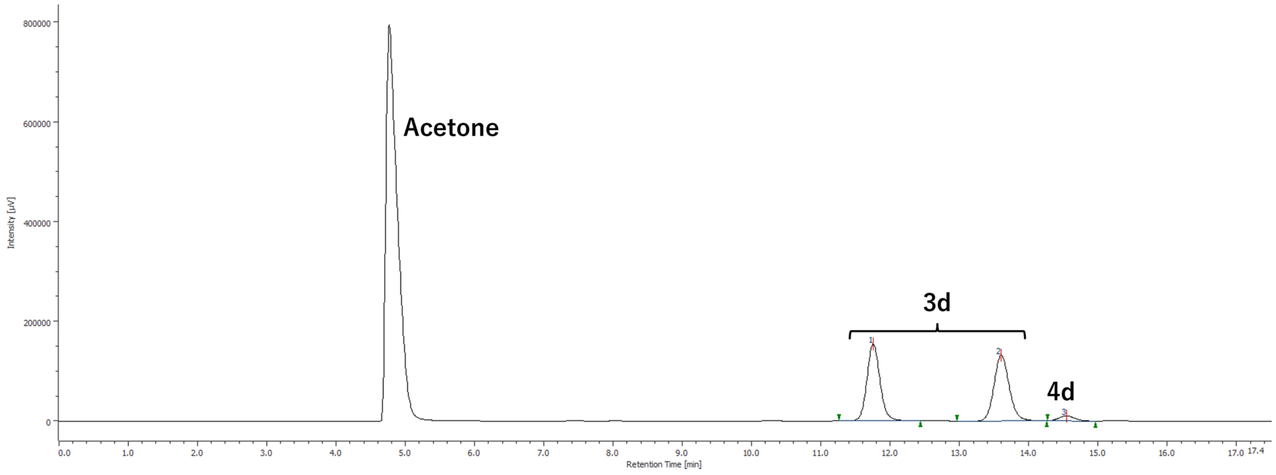

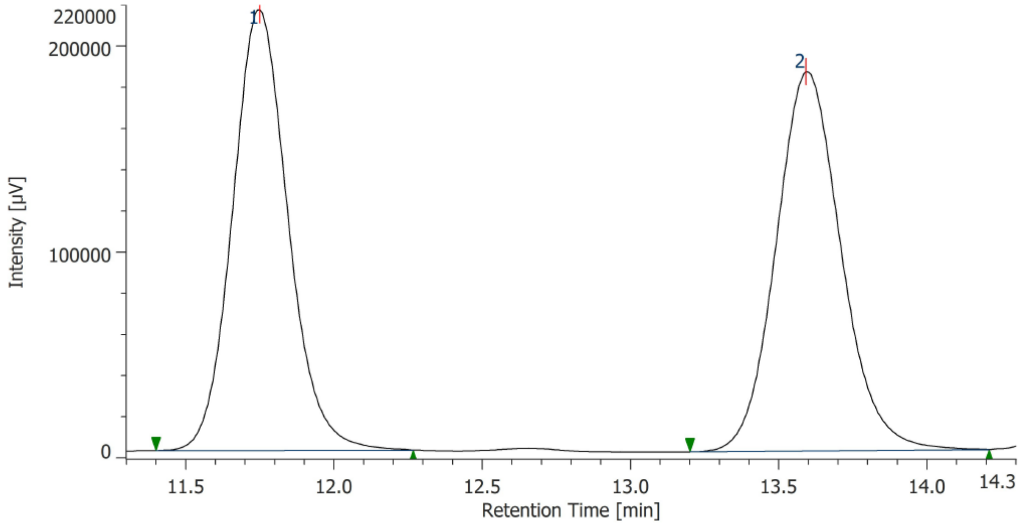

Peak No.	Retention Time (min)	Area (%)
1	11.750	46.815
2	13.592	46.564
3	14.533	6.621

3d and **4d** using (*R*)-H_8-BINAP

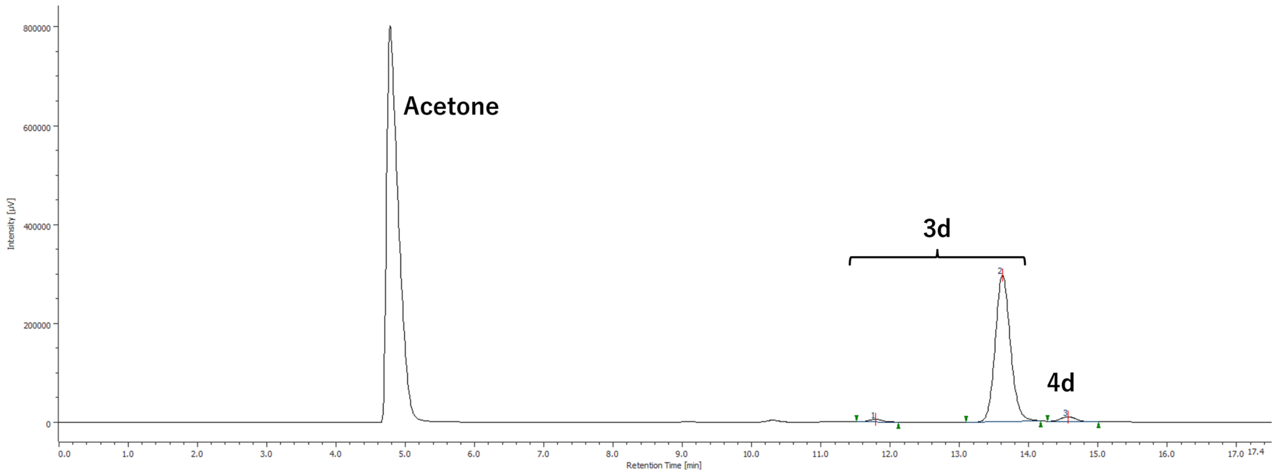

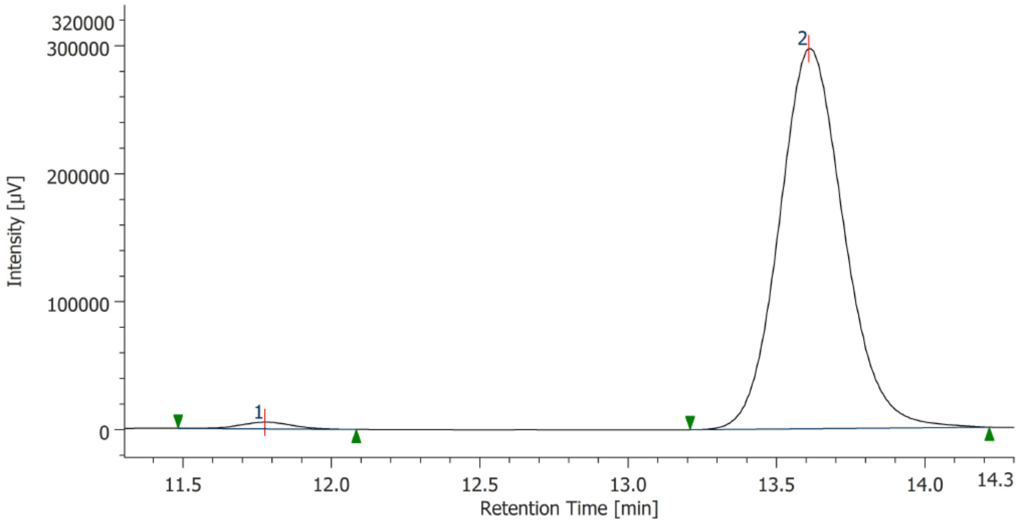

Peak No.	Retention Time (min)	Area (%)
1	11.775	1.338
2	13.608	93.032
3	14.558	5.630

***o*,*m*,*o*,*p*-Tetraphenylene 3e**
CHIRALPAK IF-3, *n*-hexane/2-propanol = 93:7, 1.0 mL min^{-1}

(±)-*cis*-**3e**, (±)-*trans*-**3e**, (±)-**4e**

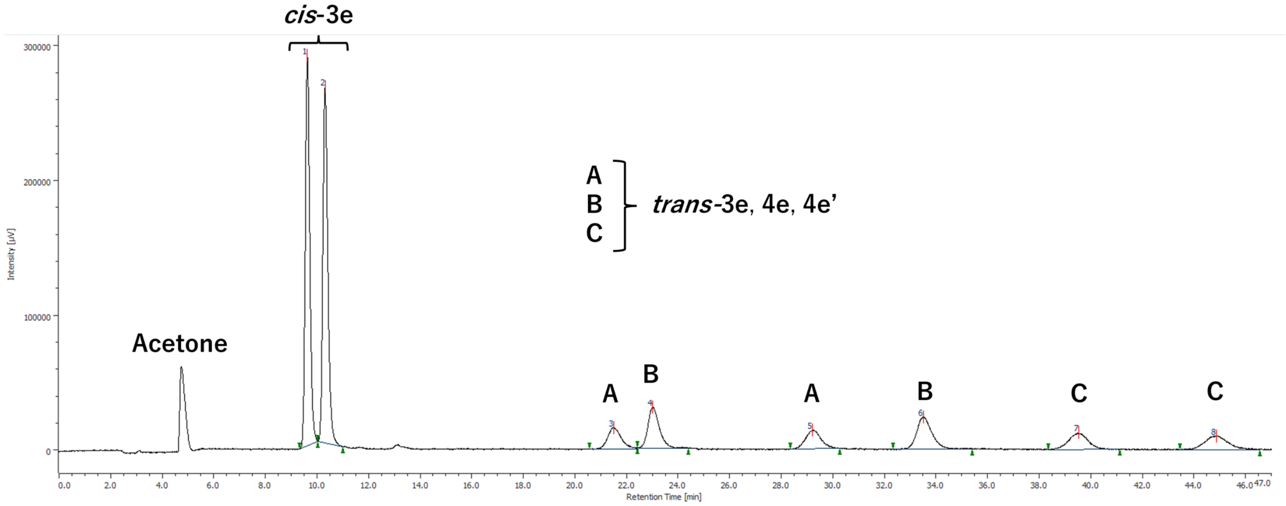

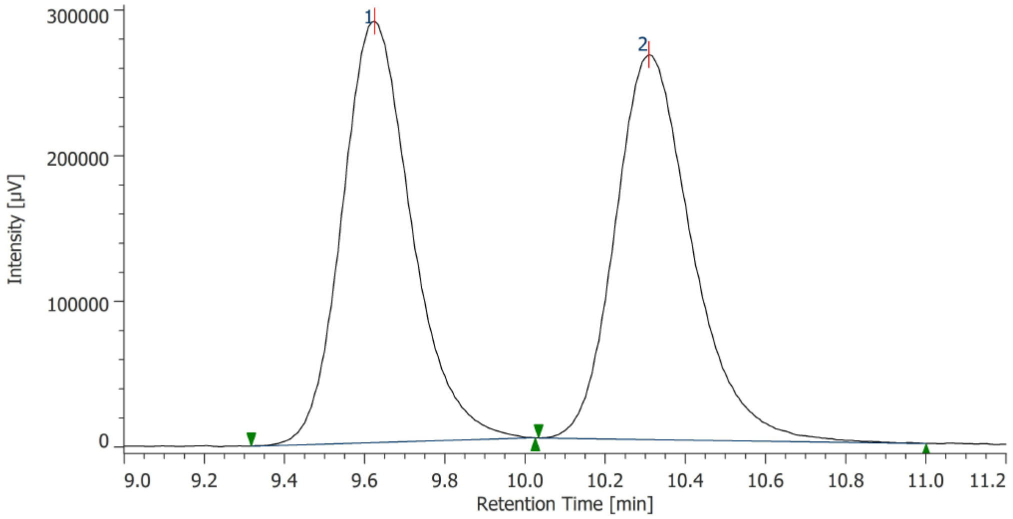

Peak No.	Retention Time (min)	Area (%)
1	9.625	30.591
2	10.308	31.043
3	21.483	5.100
4	23.008	8.419
5	29.200	5.215
6	33.500	8.474
7	39.508	5.582
8	44.850	5.574

cis-**3e**, *trans*-**3e** and **4e** using (*R*)-H_8-BINAP

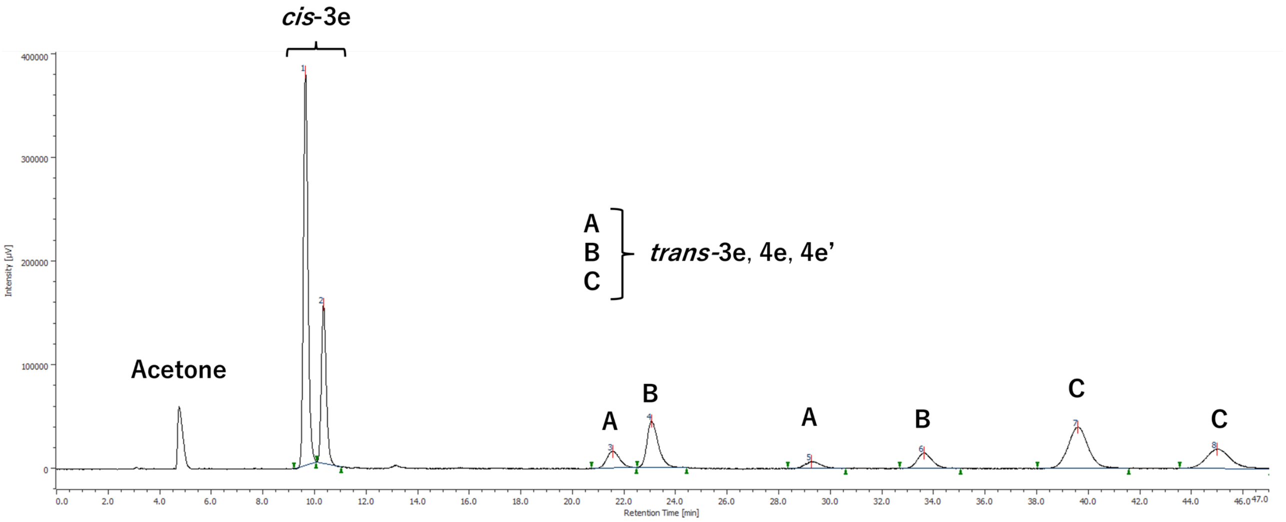

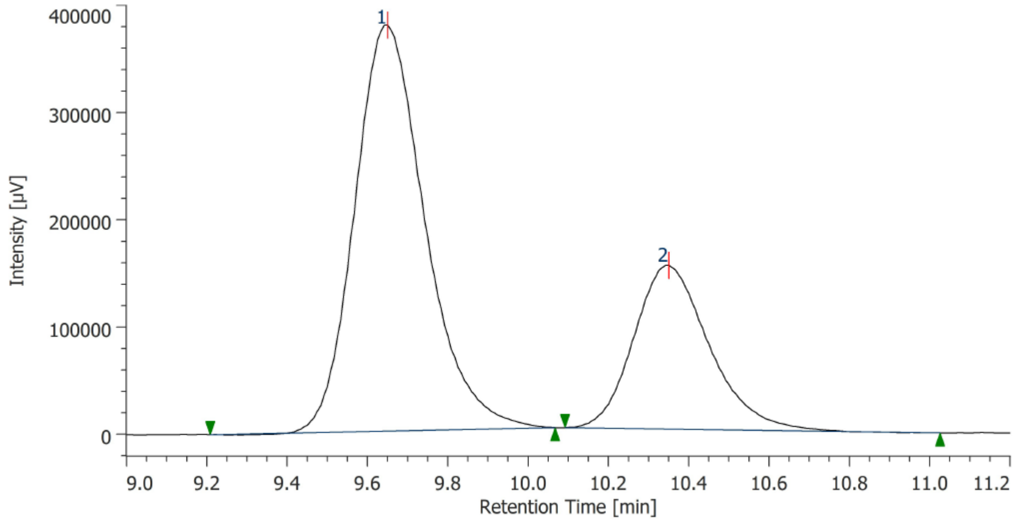

Peak No.	Retention Time (min)	Area (%)
1	9.650	36.157
2	10.350	15.640
3	21.567	4.425
4	23.050	10.788
5	29.258	2.112
6	33.625	4.557
7	39.575	16.761
8	44.975	9.559

***o*,*m*,*o*,*p*-Tetraphenylene 3f**
CHIRALPAK IF-3, *n*-hexane/2-propanol = 93:7, 1.0 mL min^{-1}

(±)-*cis*-**3f**, (±)-*trans*-**3f**, (±)-**4f**

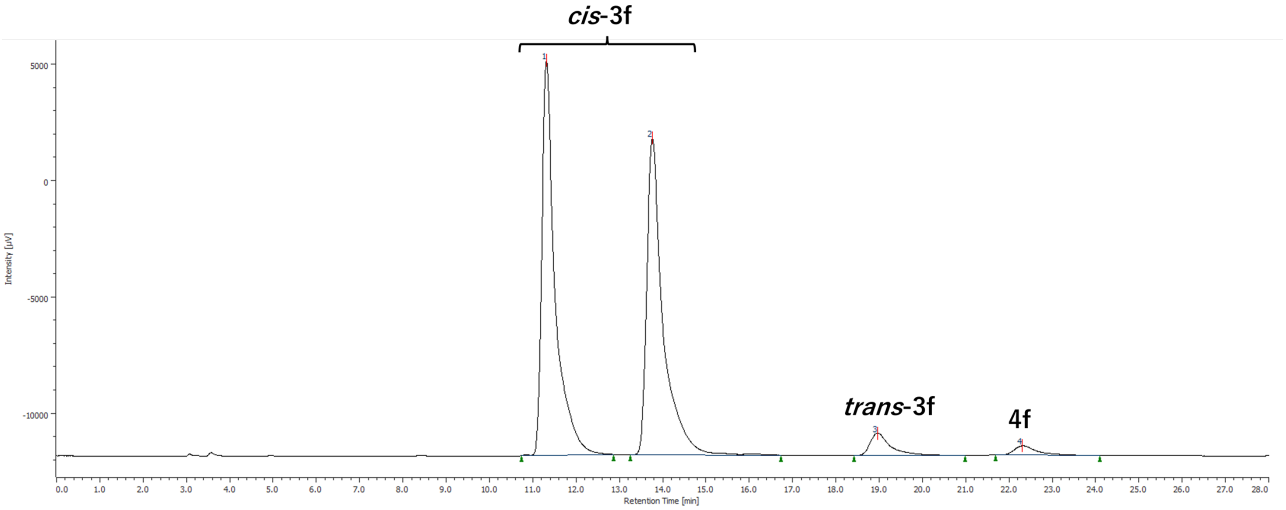

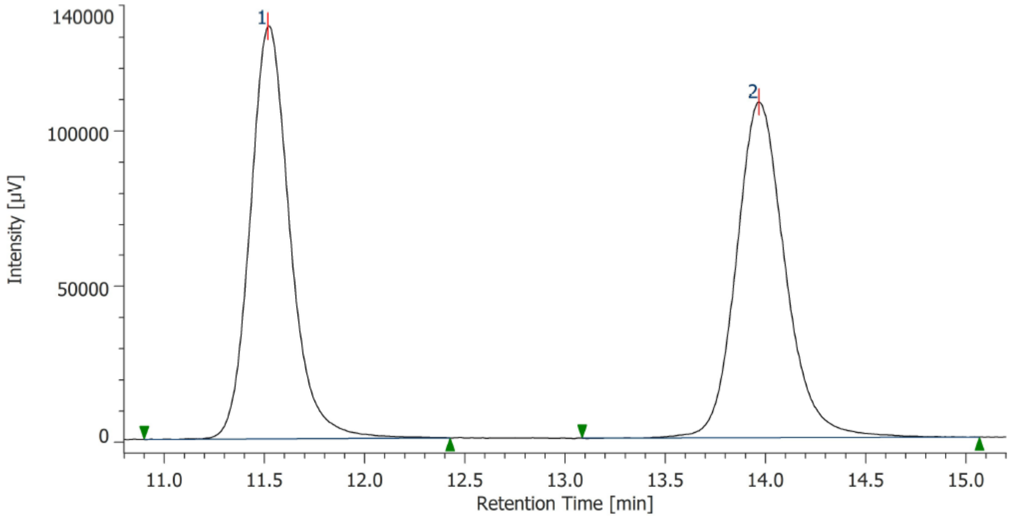

Peak No.	Retention Time (min)	Area (%)
1	11.308	47.016
2	13.758	46.850
3	18.950	4.285
4	22.292	1.849

cis-**3f** and *trans*-**3f** using (*R*)-H_8-BINAP

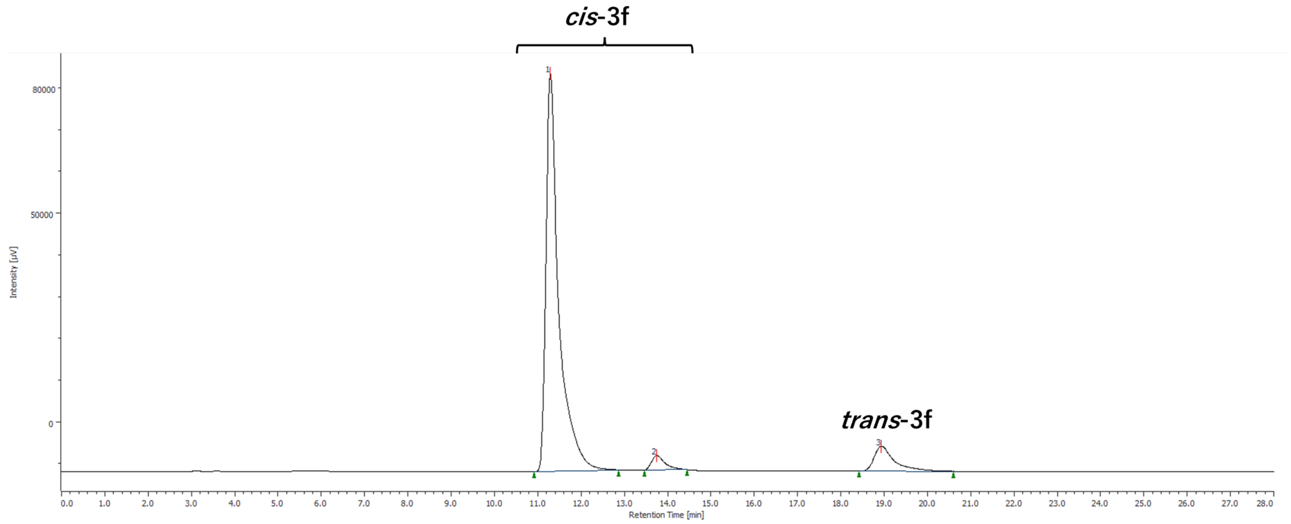

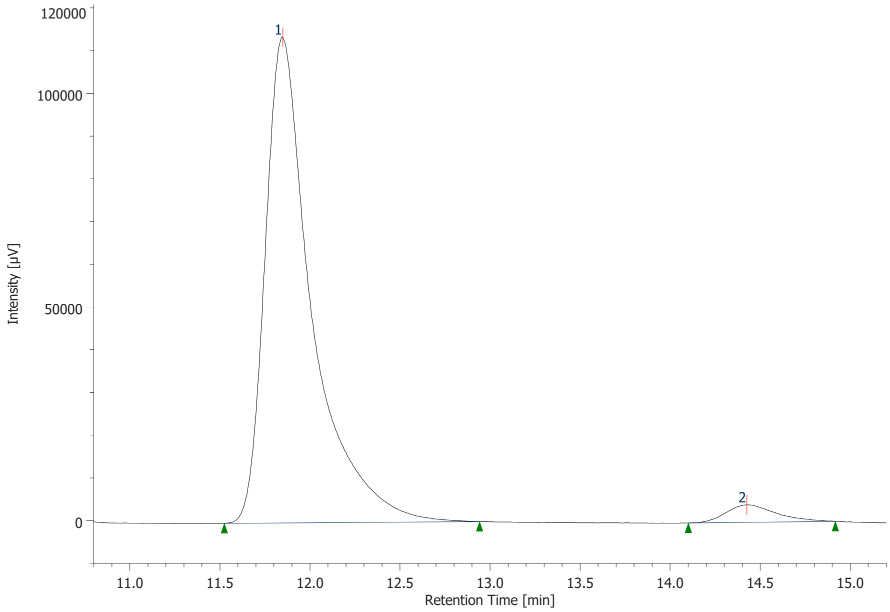

Peak No.	Retention Time (min)	Area (%)
1	11.283	87.864
2	13.742	3.364
3	18.925	8.773

8. ^{1}H and ^{13}C NMR Spectra

2,2''-Diethynyl-5'-methyl-1,1':3',1''-terphenyl (1a)

^{1}H NMR ($CDCl_3$, 400 MHz)

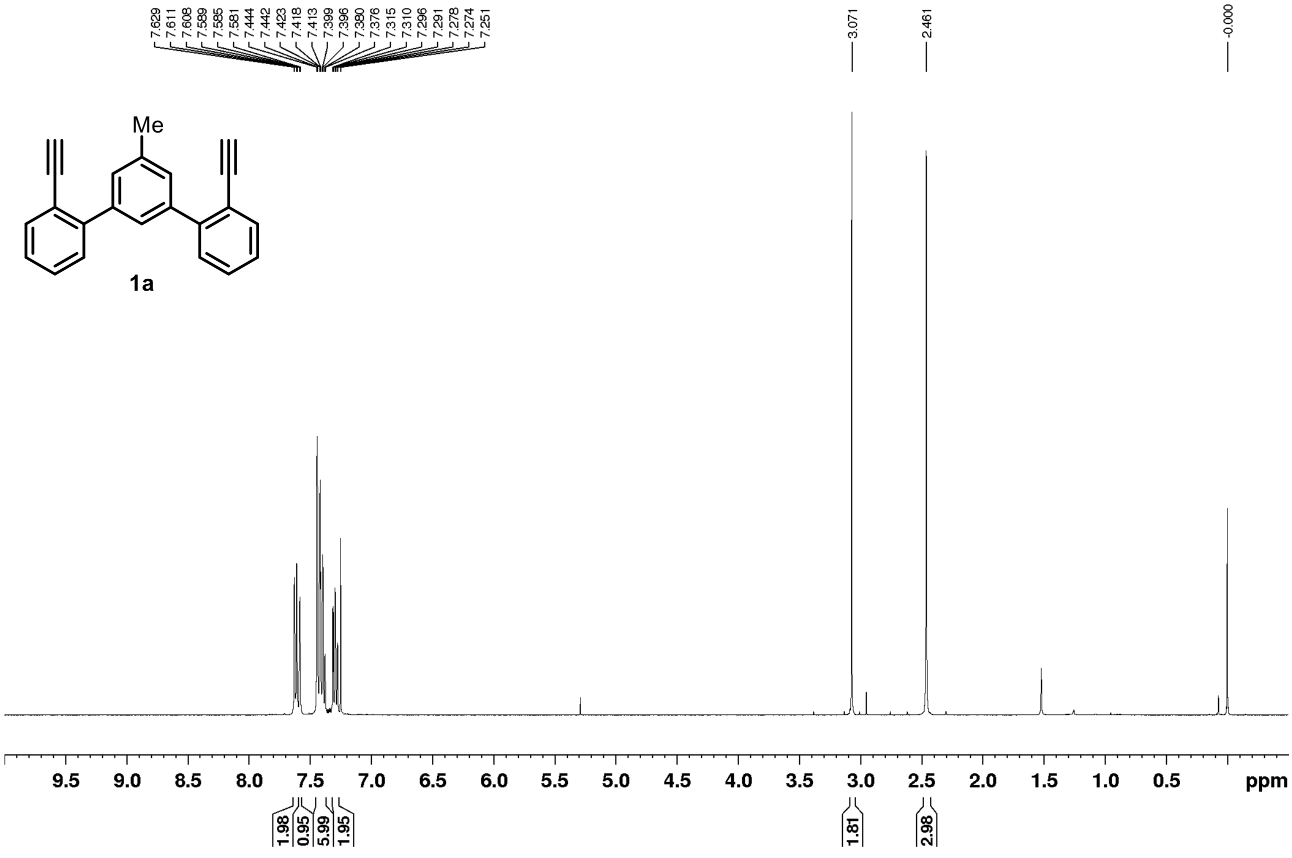

^{13}C NMR ($CDCl_3$, 100 MHz)

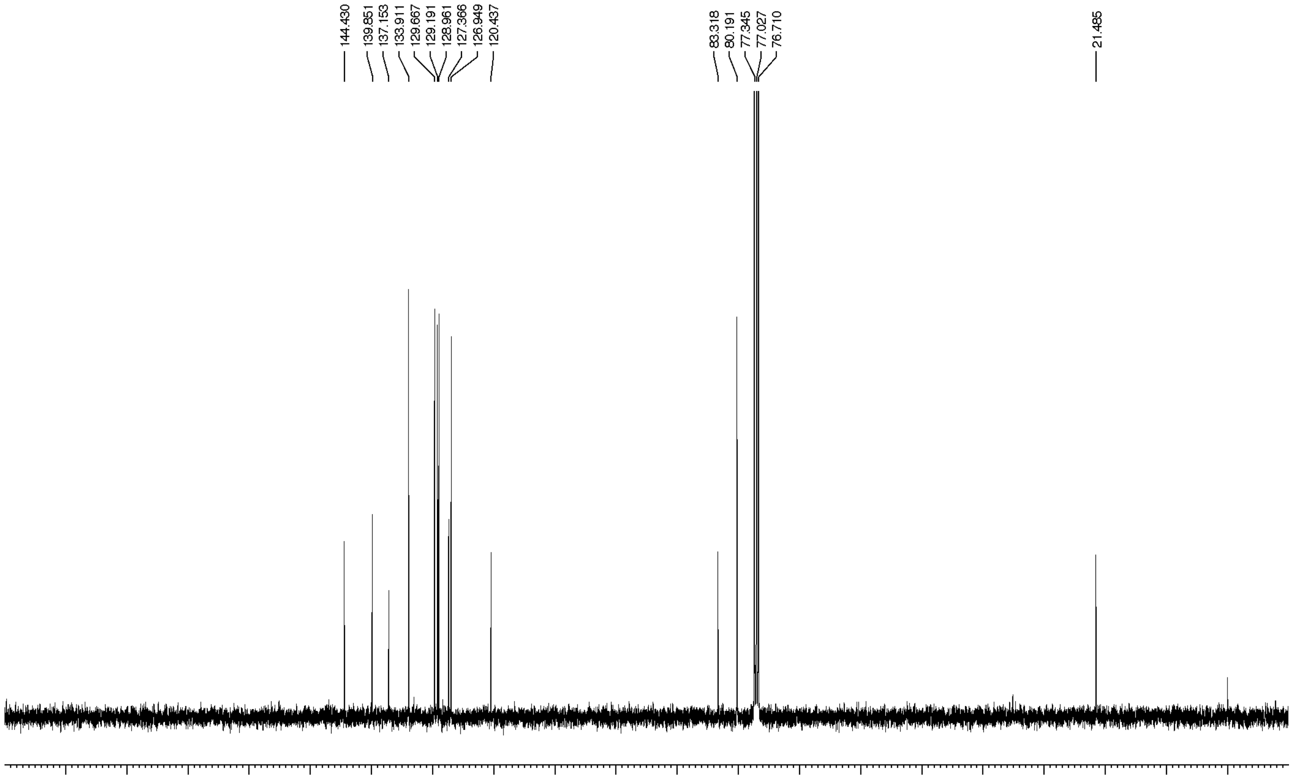

2,2''-Diethynyl-4',6'-dimethyl-1,1':3',1''-terphenyl (1b)

^{1}H NMR ($CDCl_3$, 400 MHz)

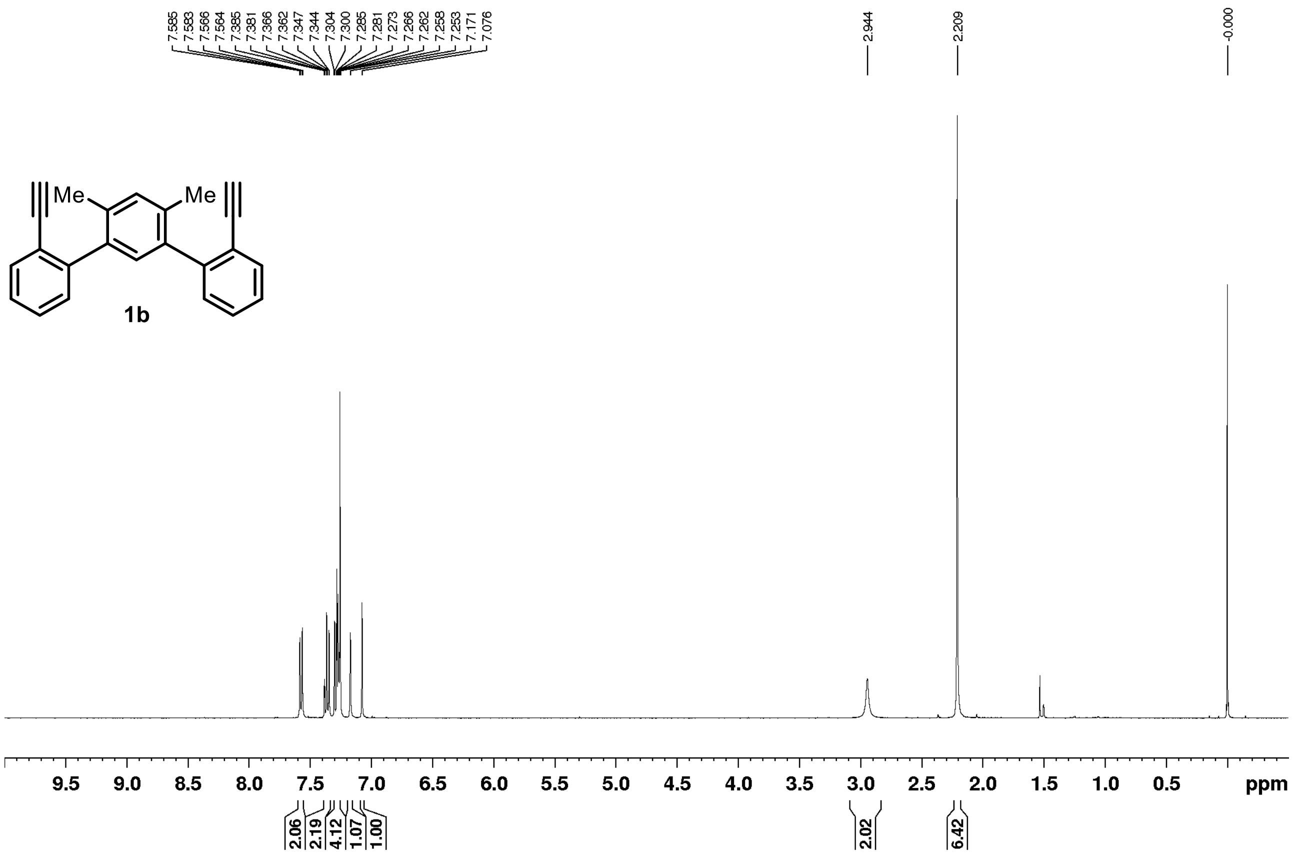

^{13}C NMR ($CDCl_3$, 100 MHz)

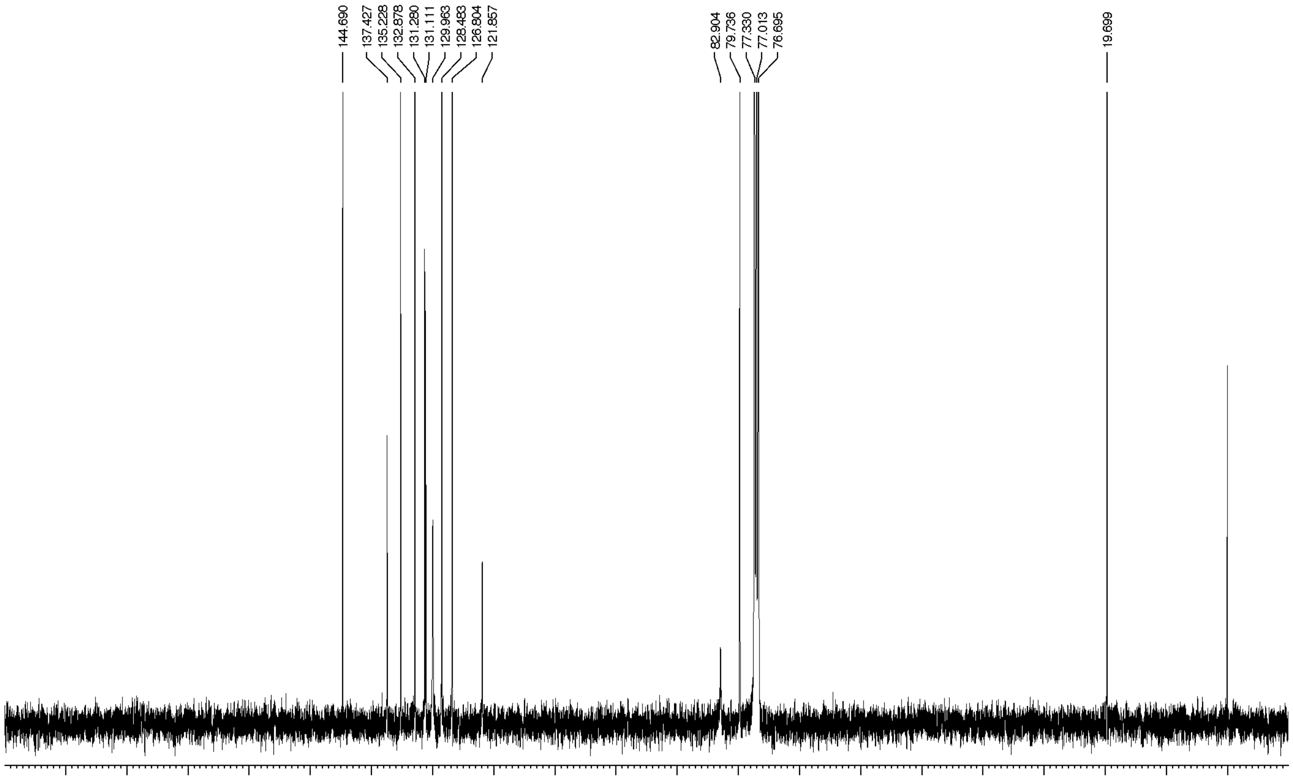

1-((Triisopropylsilyl)ethynyl)naphthalen-2-yl trifluoromethanesulfonate (S7)

^{1}H NMR ($CDCl_3$, 400 MHz)

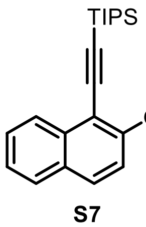

S7

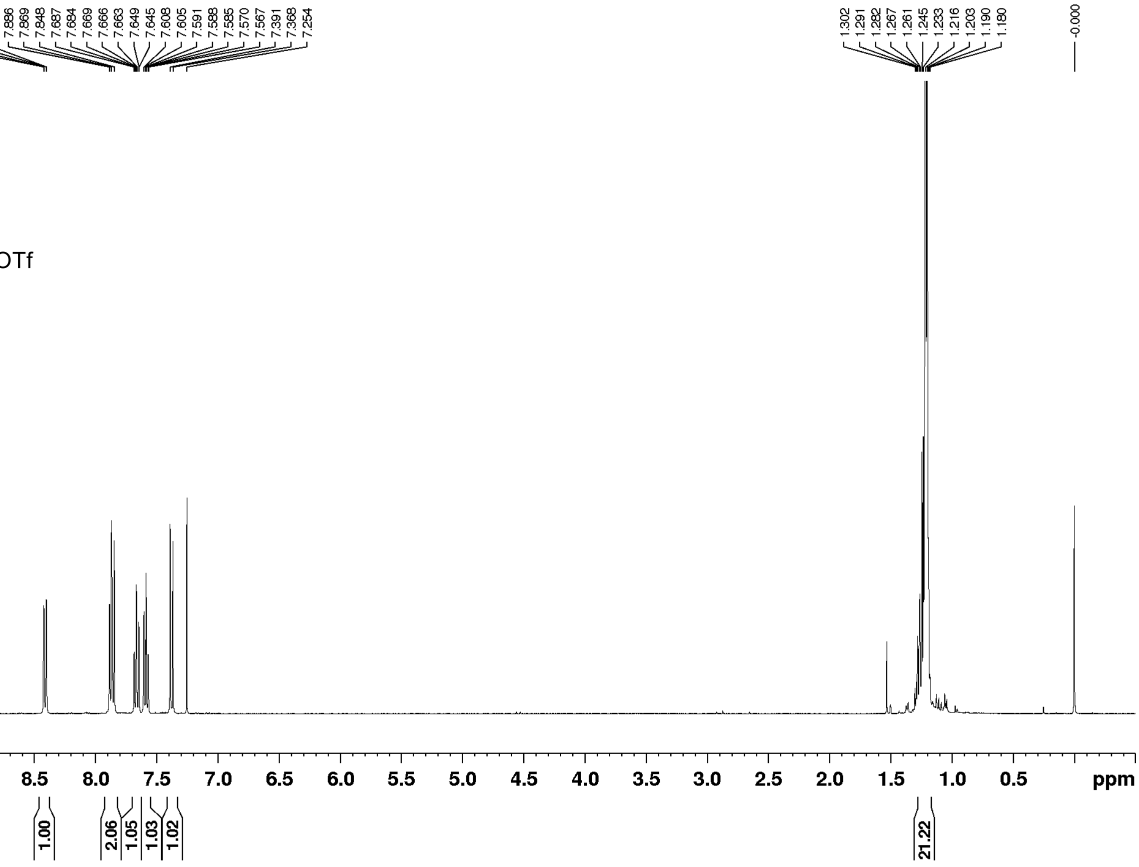

^{13}C NMR ($CDCl_3$, 100 MHz)

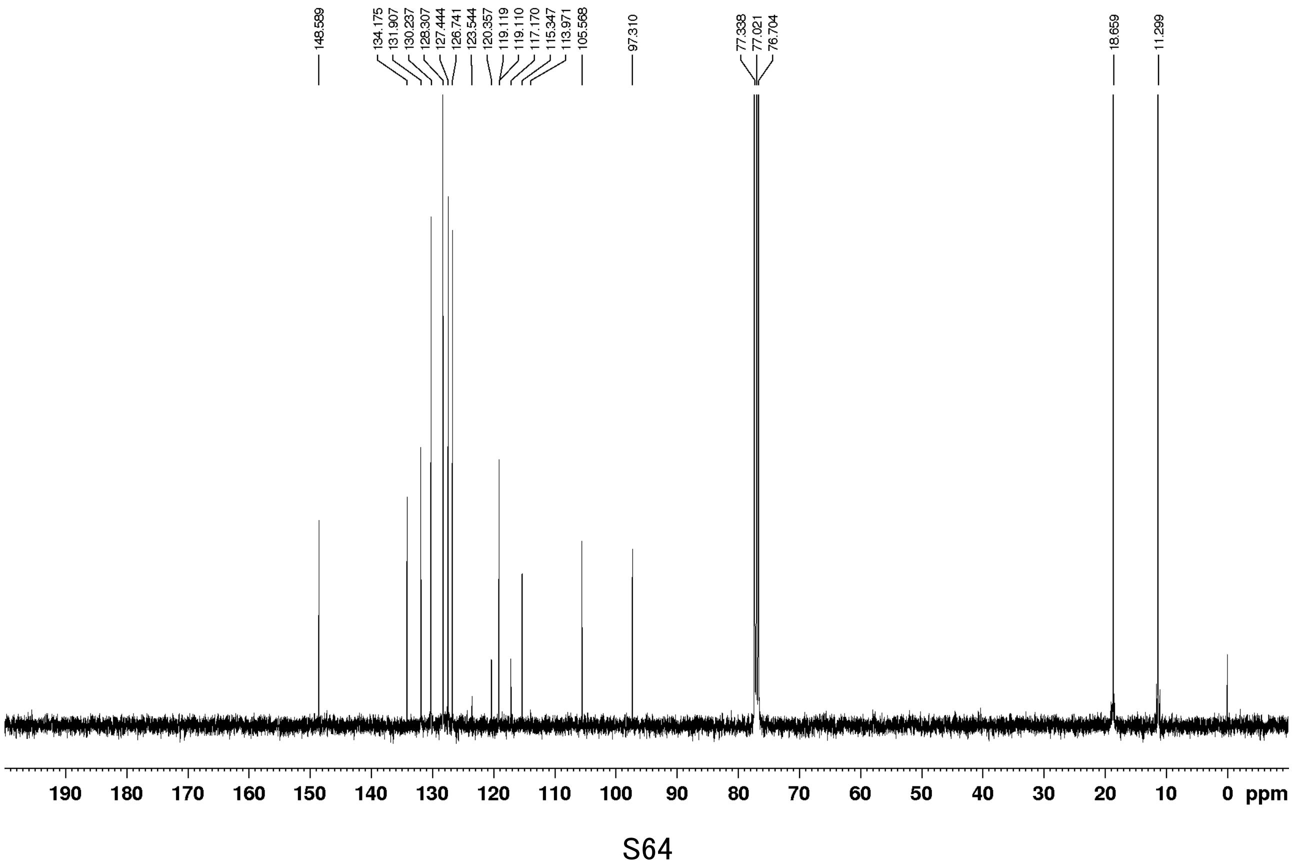

((3'-Bromo-5'-methyl-[1,1'-biphenyl]-2-yl)ethynyl)triisopropylsilane (S8)

^{1}H NMR ($CDCl_3$, 400 MHz)

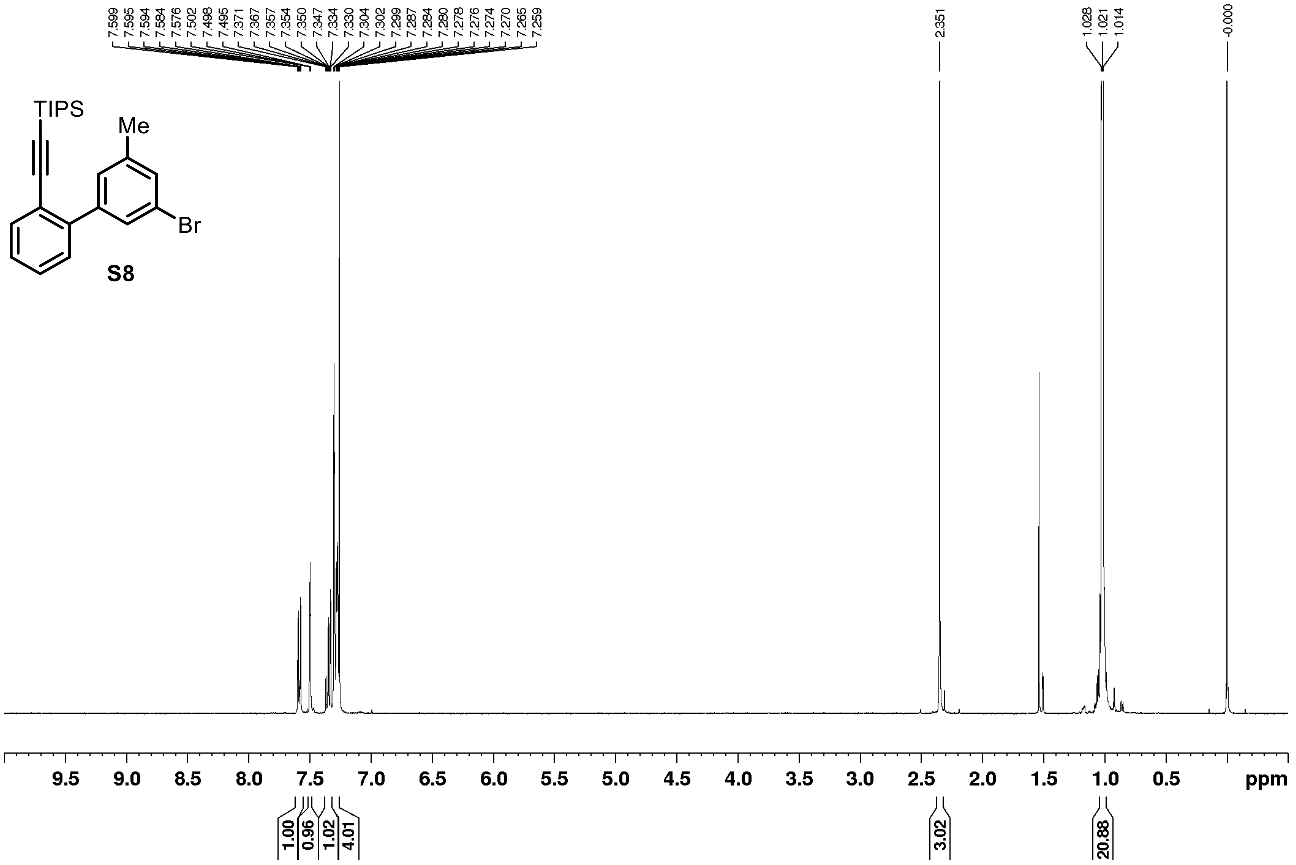

^{13}C NMR ($CDCl_3$, 100 MHz)

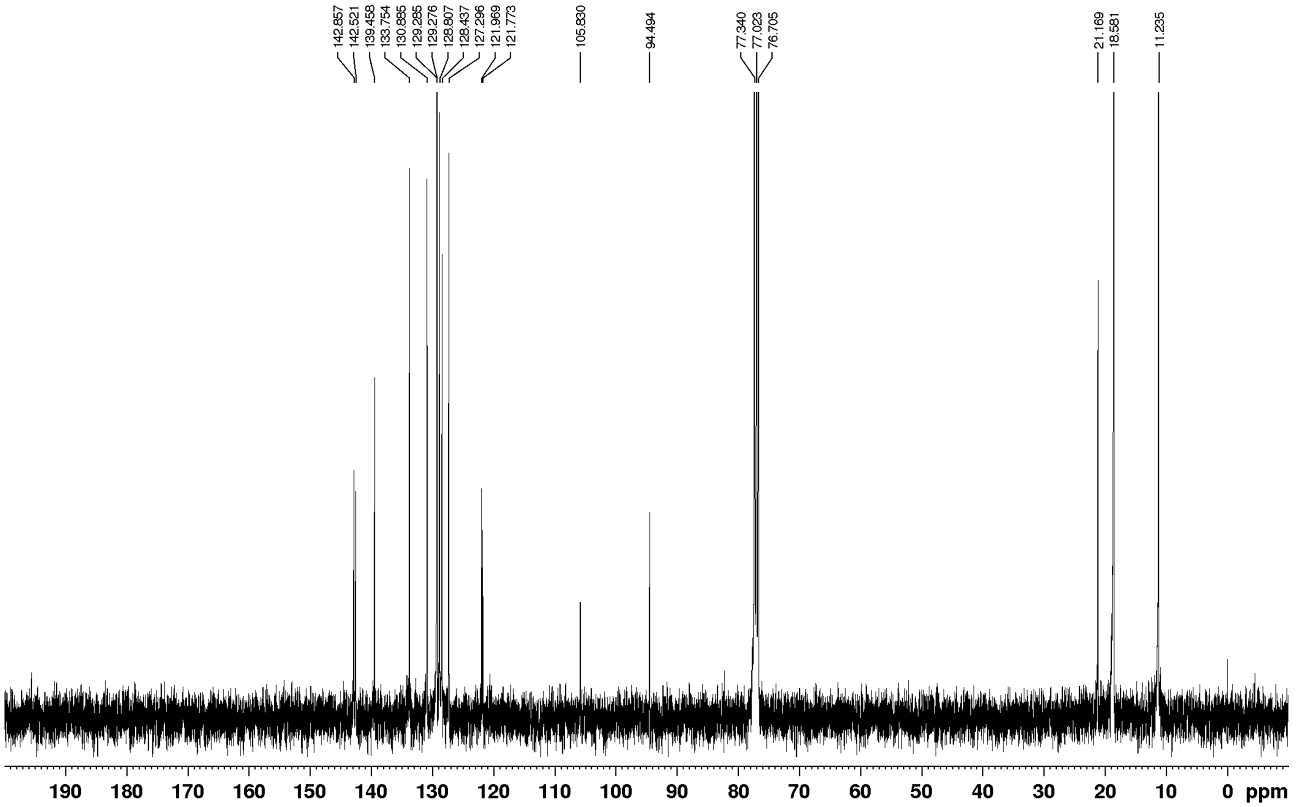

Triisopropyl((3'-methyl-5'-(4,4,5,5-tetramethyl-1,3,2-dioxaborolan-2-yl)-[1,1'-biphenyl]-2-yl)ethynyl)silane (S9)

^{1}H NMR ($CDCl_3$, 400 MHz)

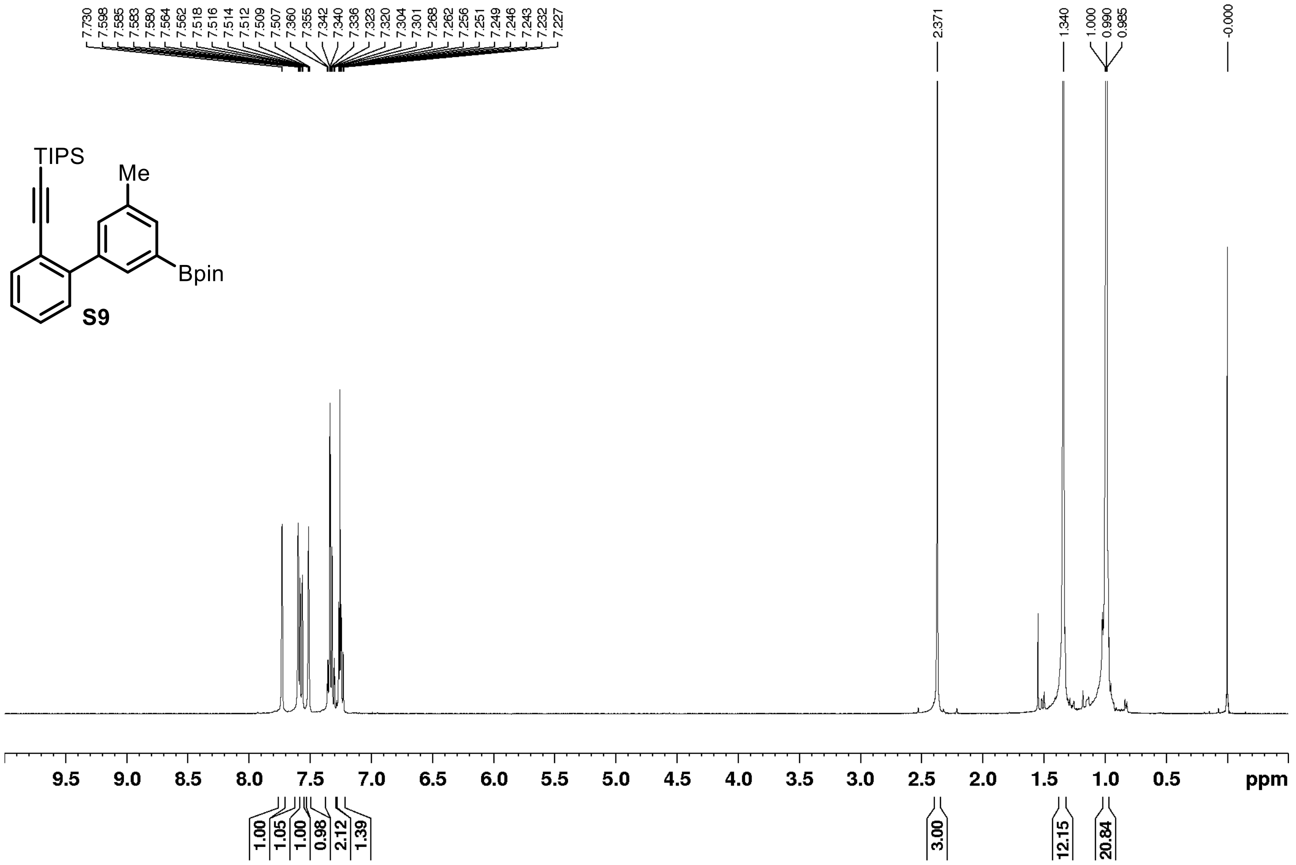

^{13}C NMR ($CDCl_3$, 100 MHz)

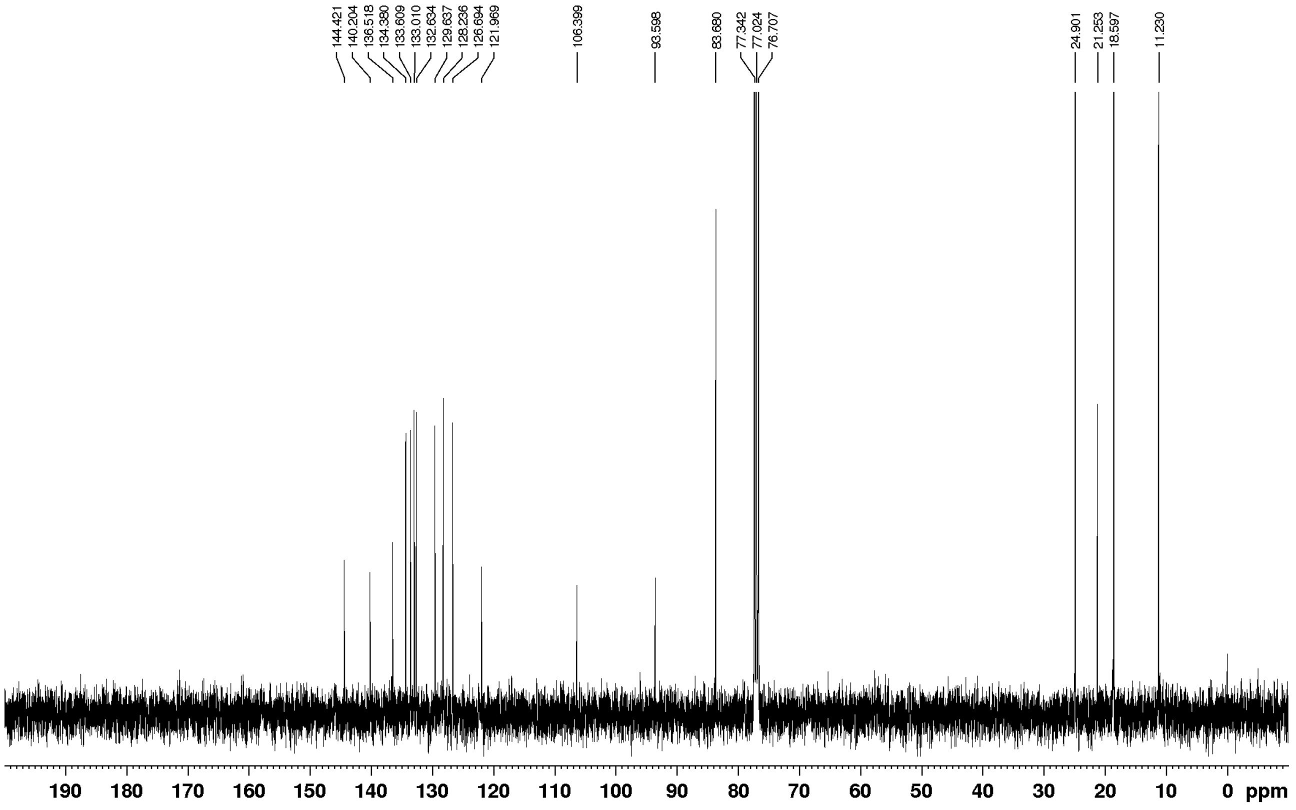

1-Ethynyl-2-(2'-ethynyl-5-methyl-[1,1'-biphenyl]-3-yl)naphthalene (1c)

^{1}H NMR (CDCl$_3$, 400 MHz)

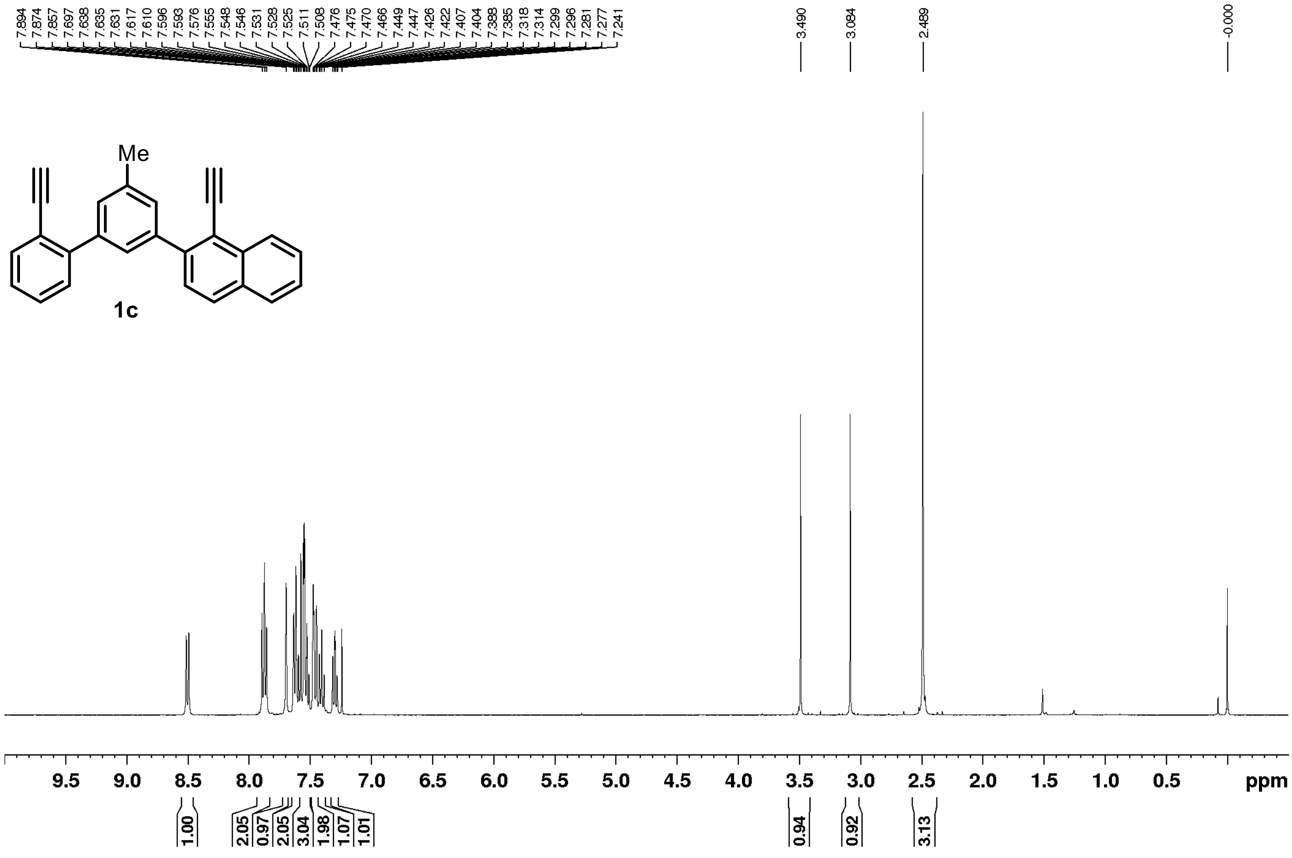

^{13}C NMR (CDCl$_3$, 100 MHz)

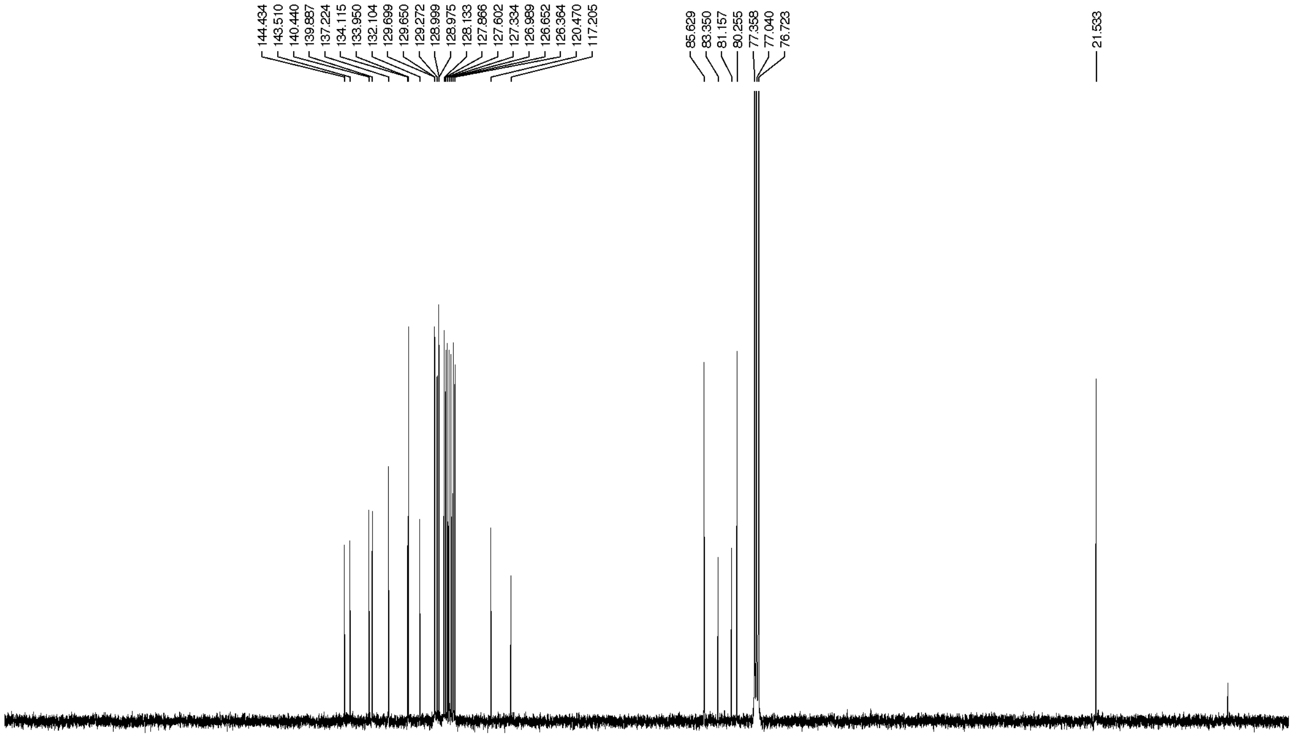

((3'-romo-5'-chloro-[1,1'-biphenyl]-2-yl)ethynyl)triisopropylsilane (S12)

^{1}H NMR (CDCl$_3$, 400 MHz)

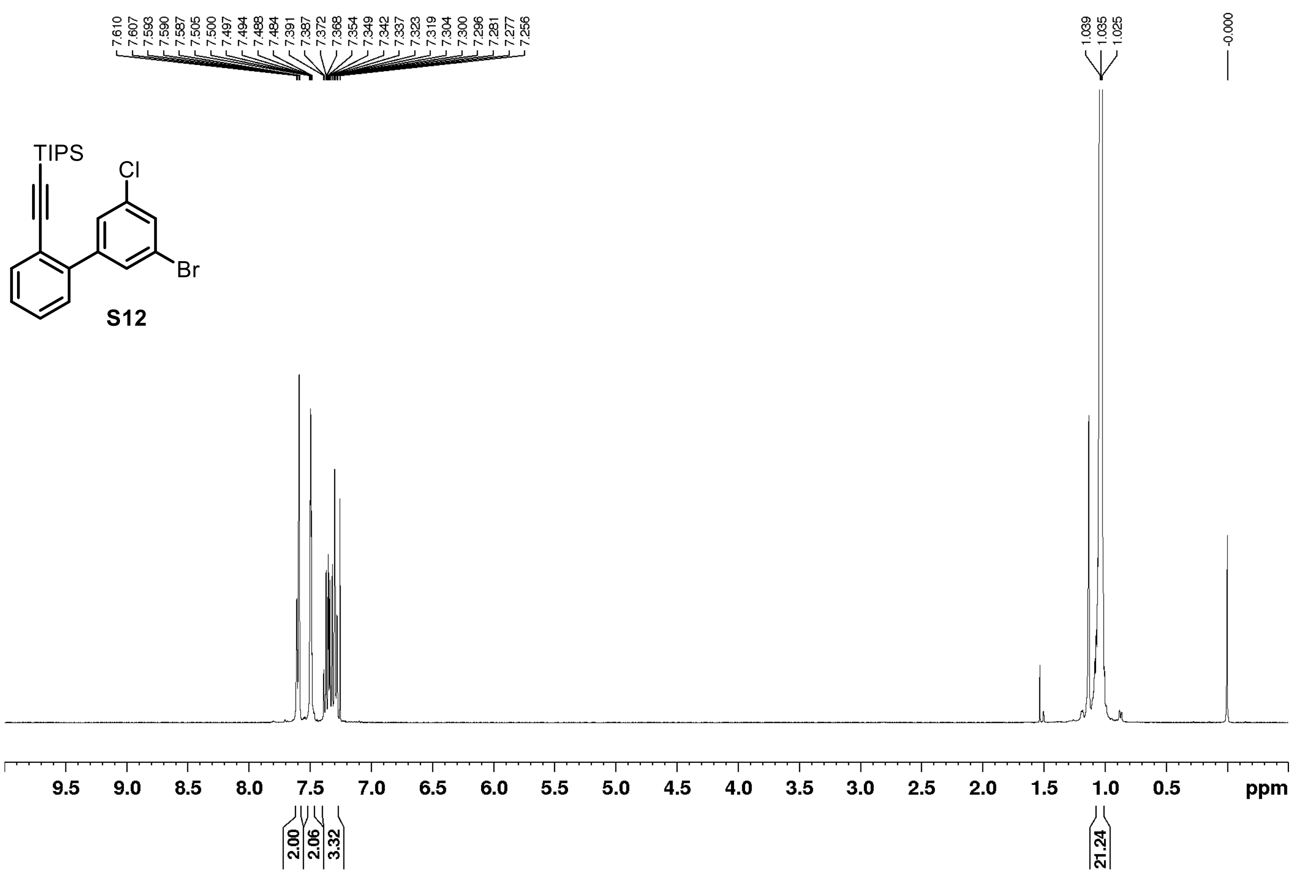

^{13}C NMR (CDCl$_3$, 100 MHz)

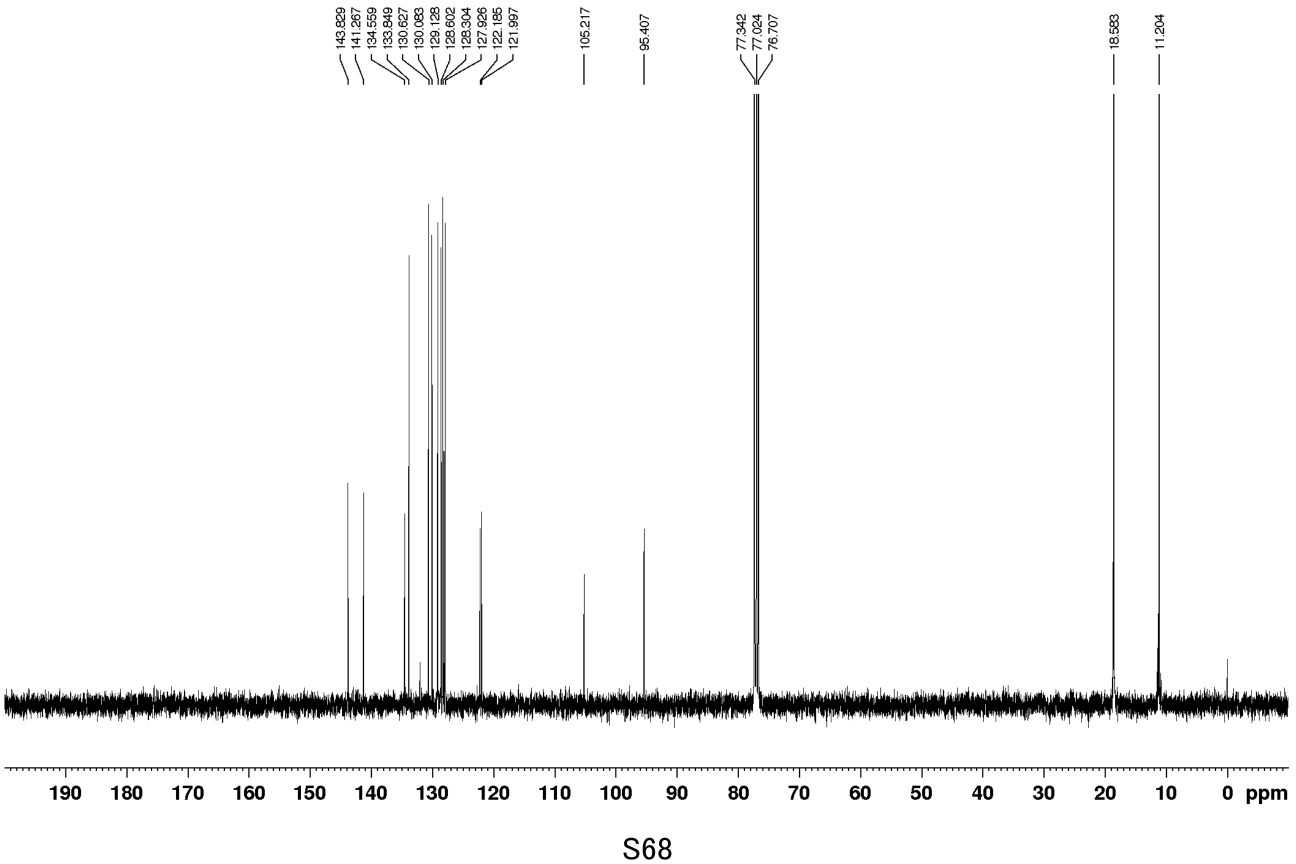

((3'-Chloro-5'-(4,4,5,5-tetramethyl-1,3,2-dioxaborolan-2-yl)-[1,1'-biphenyl]-2-yl)ethynyl)-triisopropylsilane (S13)

^{1}H NMR ($CDCl_3$, 400 MHz)

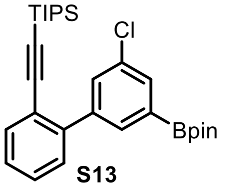

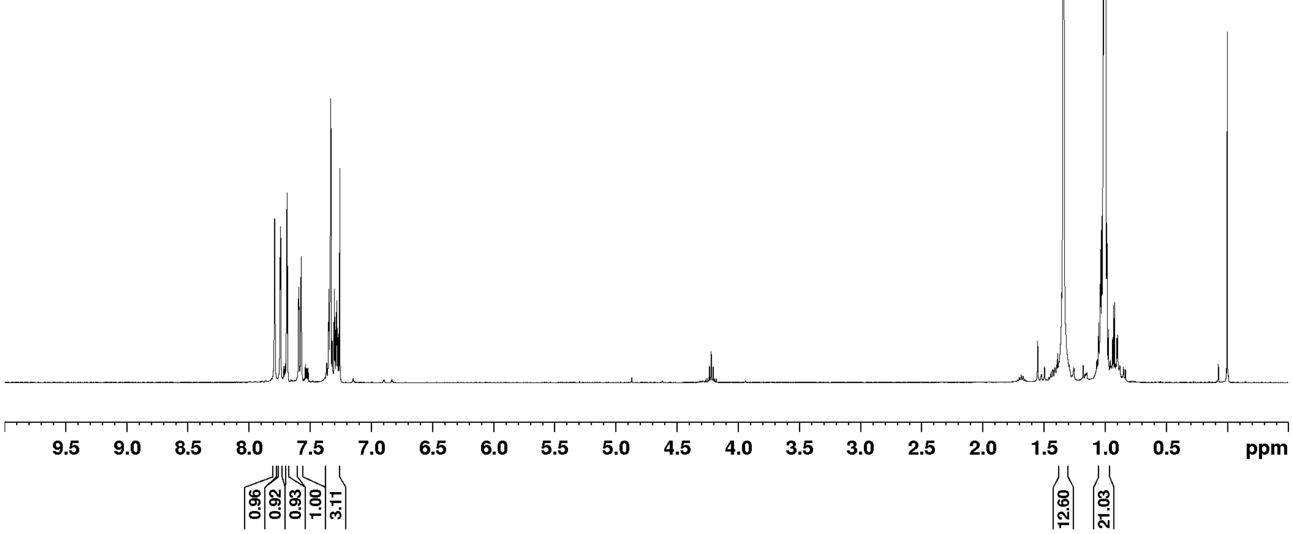

^{13}C NMR ($CDCl_3$, 100 MHz)

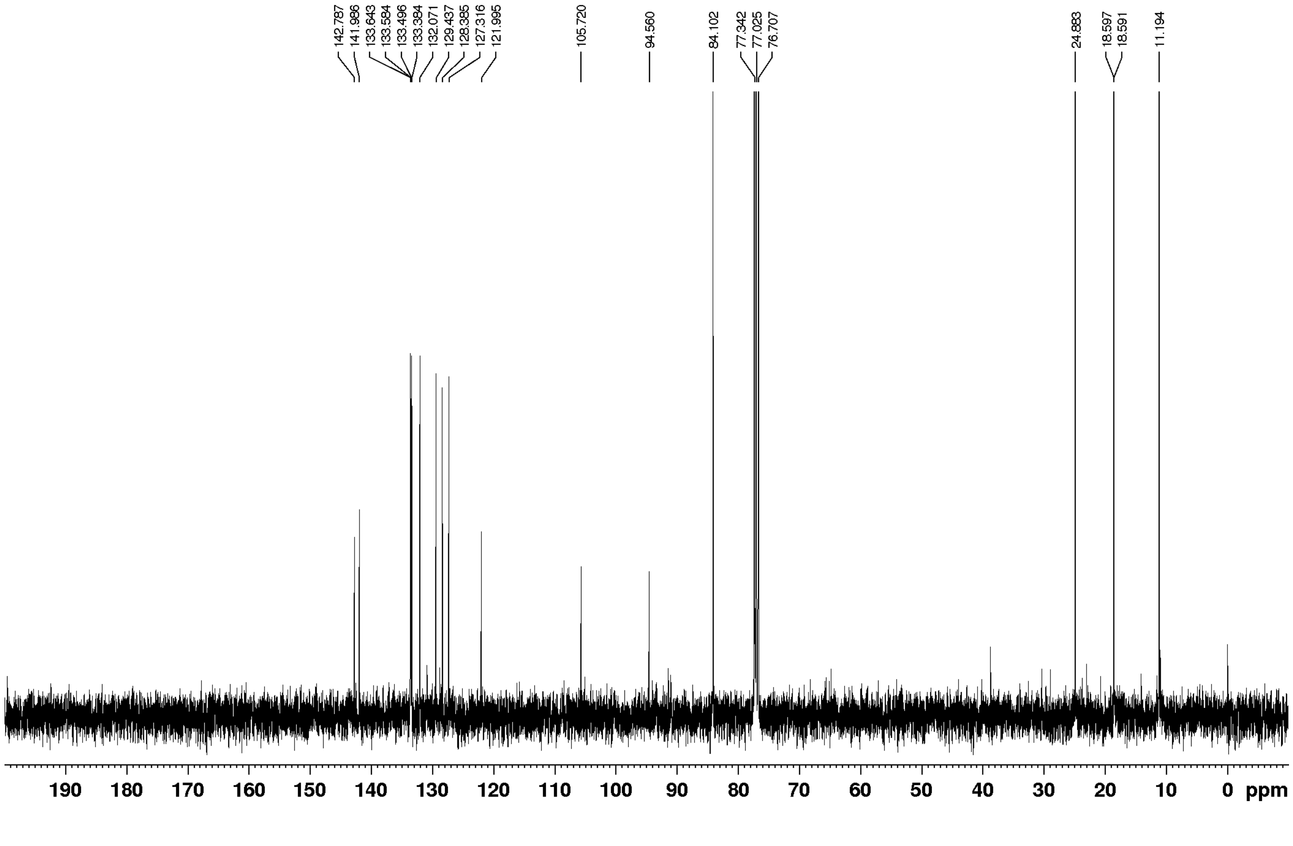

2-(5-Chloro-2'-ethynyl-[1,1'-biphenyl]-3-yl)-1-ethynylnaphthalene (1d)

^{1}H NMR ($CDCl_3$, 400 MHz)

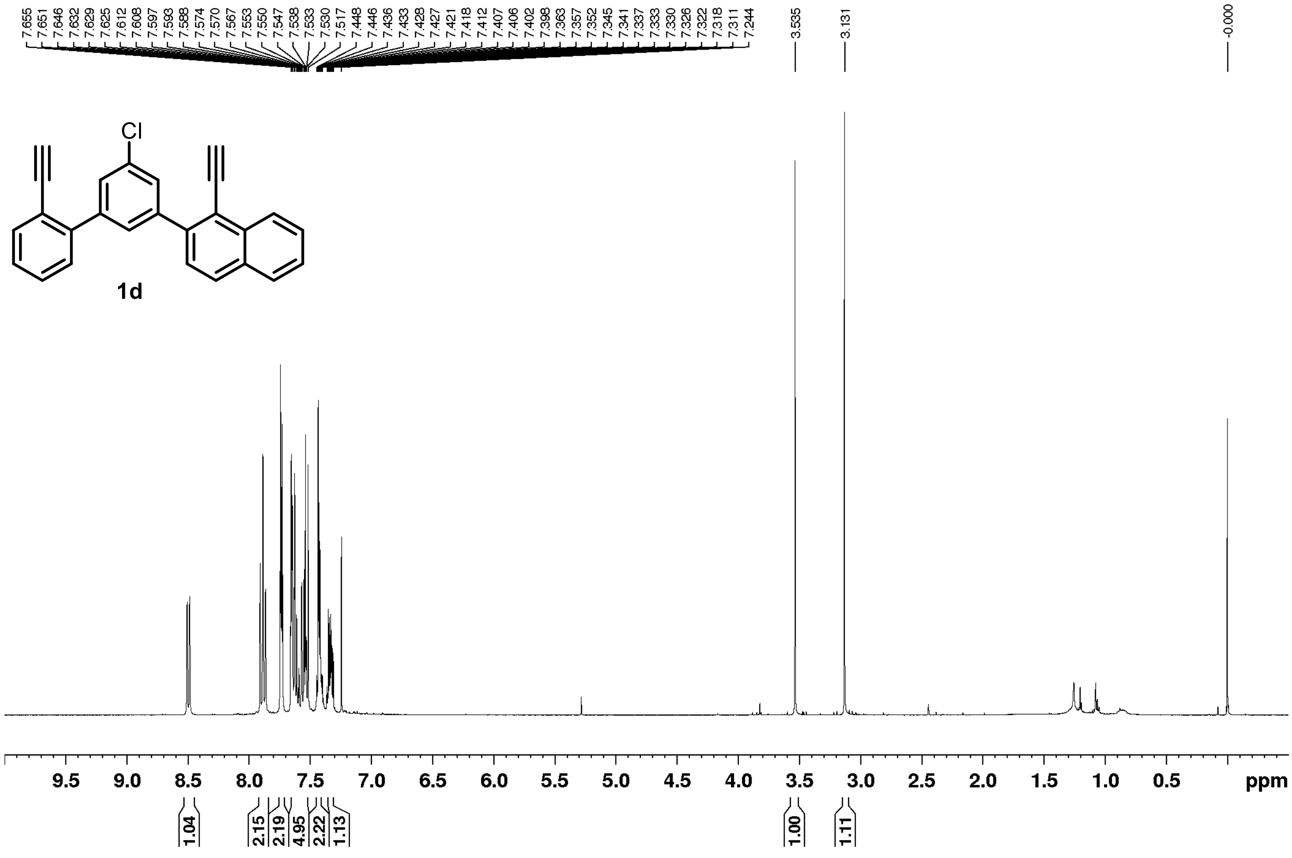

^{13}C NMR ($CDCl_3$, 100 MHz)

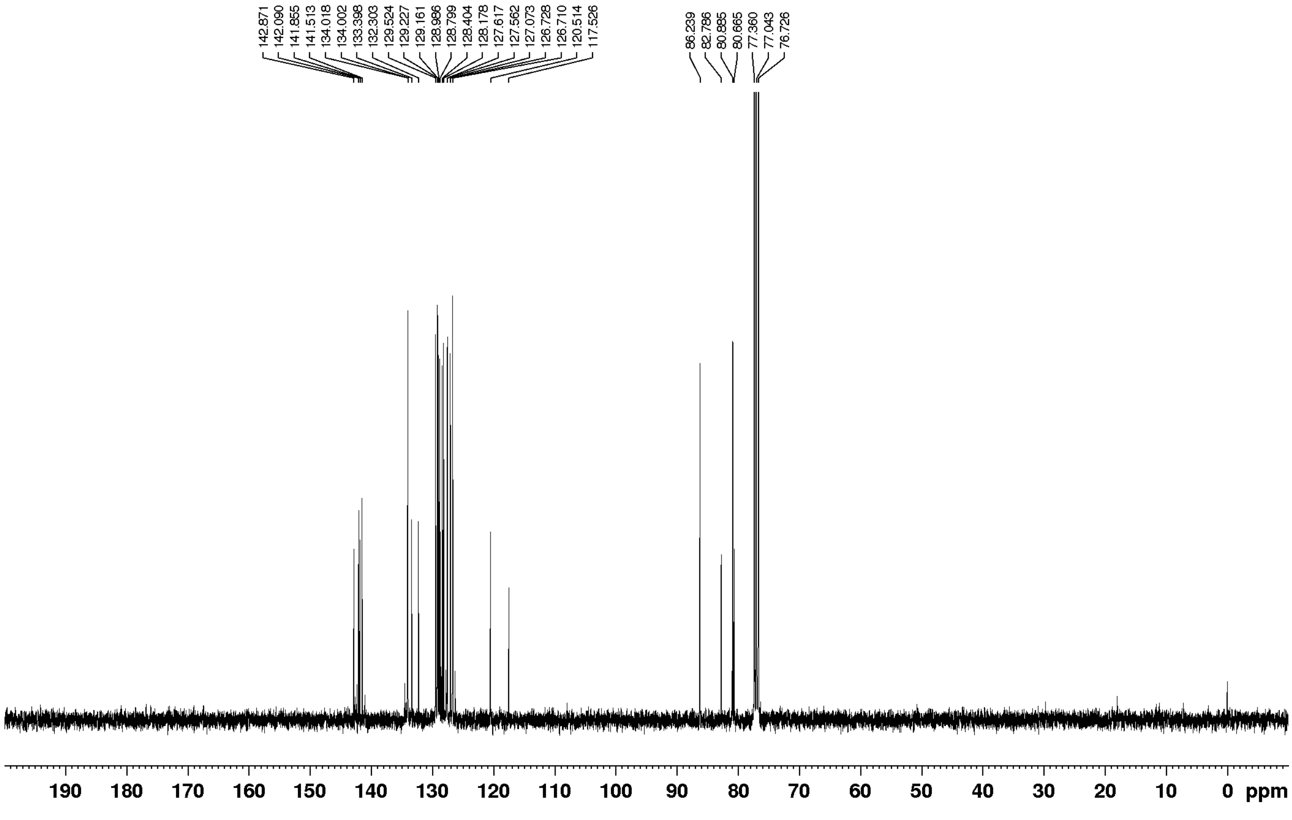

((1-Bromonaphthalen-2-yl)ethynyl)triisopropylsilane (S16)

^{1}H NMR ($CDCl_3$, 400 MHz)

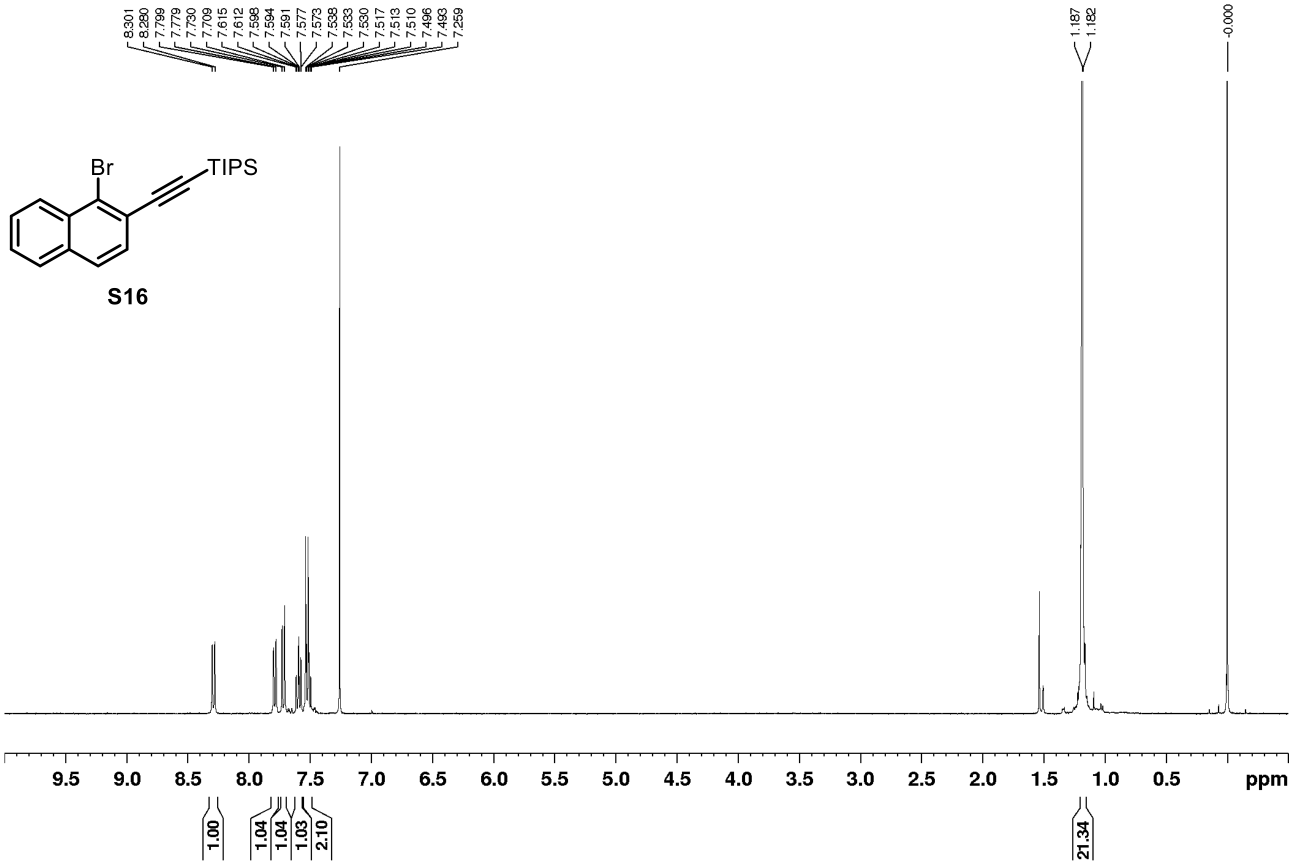

^{13}C NMR ($CDCl_3$, 100 MHz)

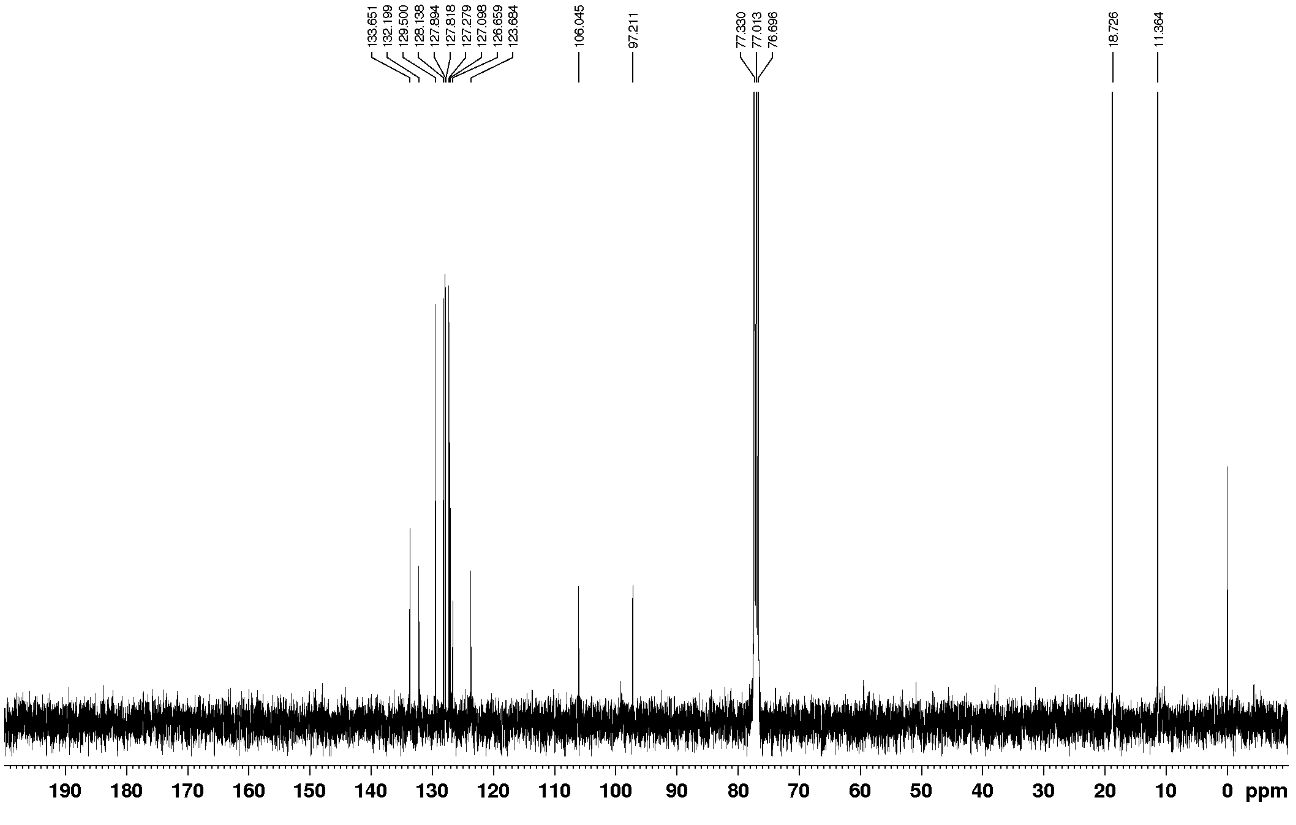

(2-((Triisopropylsilyl)ethynyl)naphthalen-1-yl)boronic acid (S17)

^{1}H NMR ($CDCl_3$, 400 MHz)

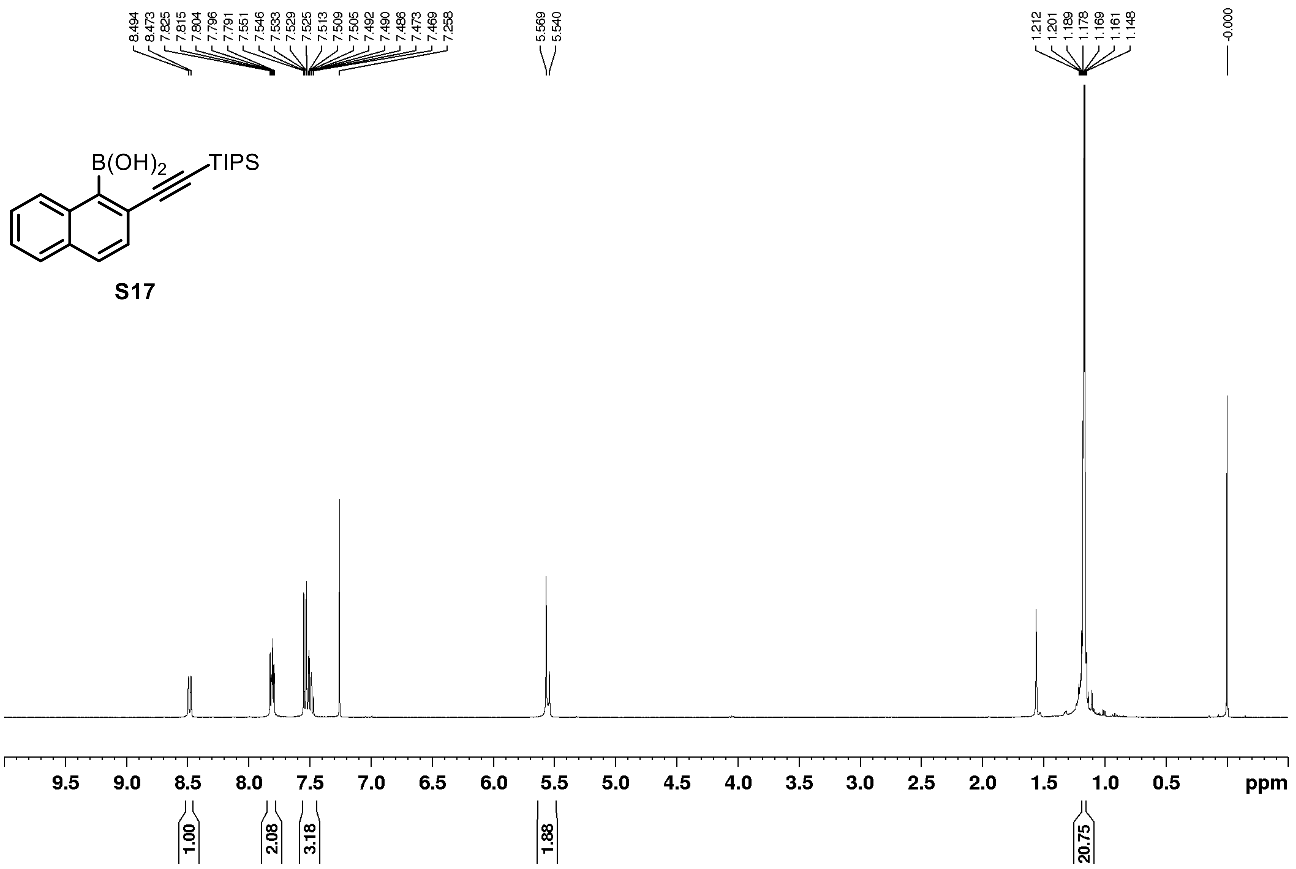

^{13}C NMR ($CDCl_3$, 100 MHz)

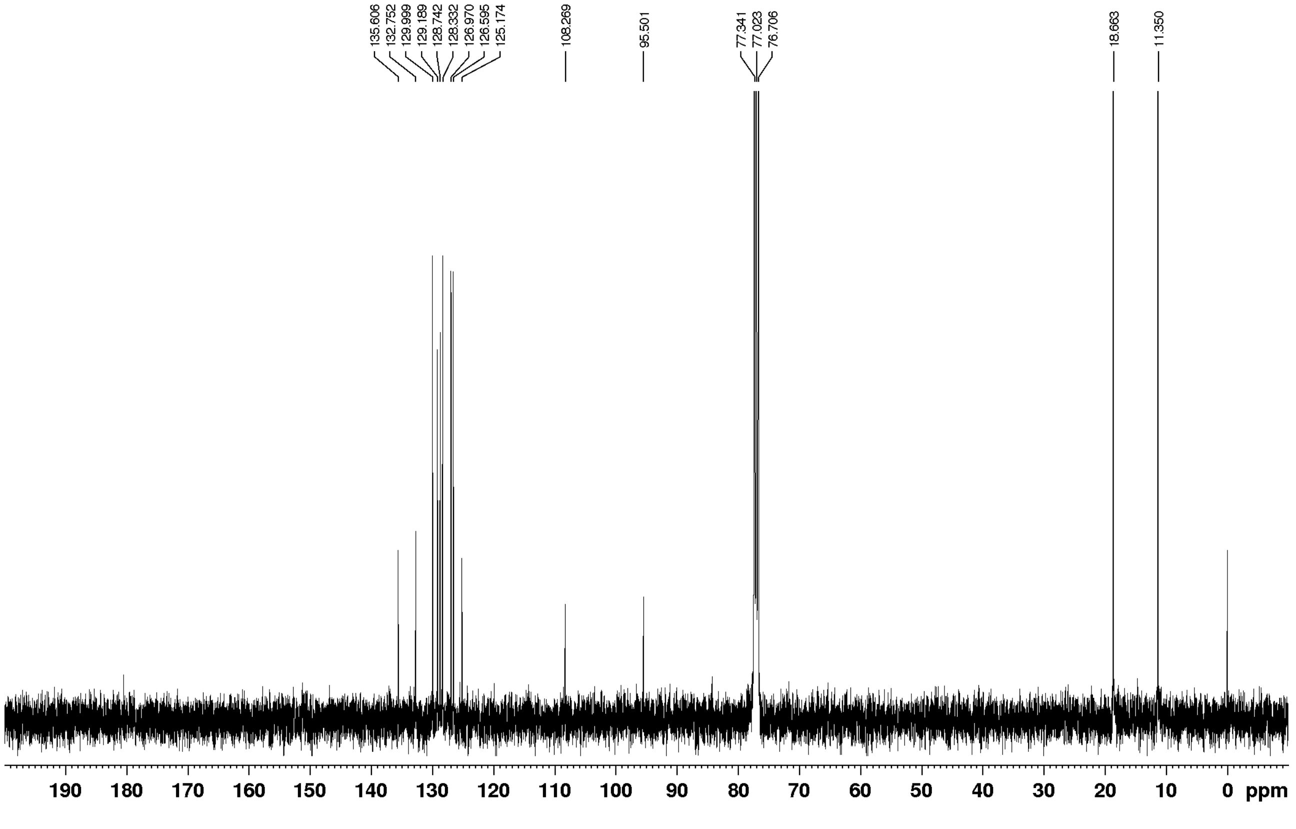

2-Ethynyl-1-(2'-ethynyl-5-methyl-[1,1'-biphenyl]-3-yl)naphthalene (1e)

^{1}H NMR ($CDCl_3$, 400 MHz)

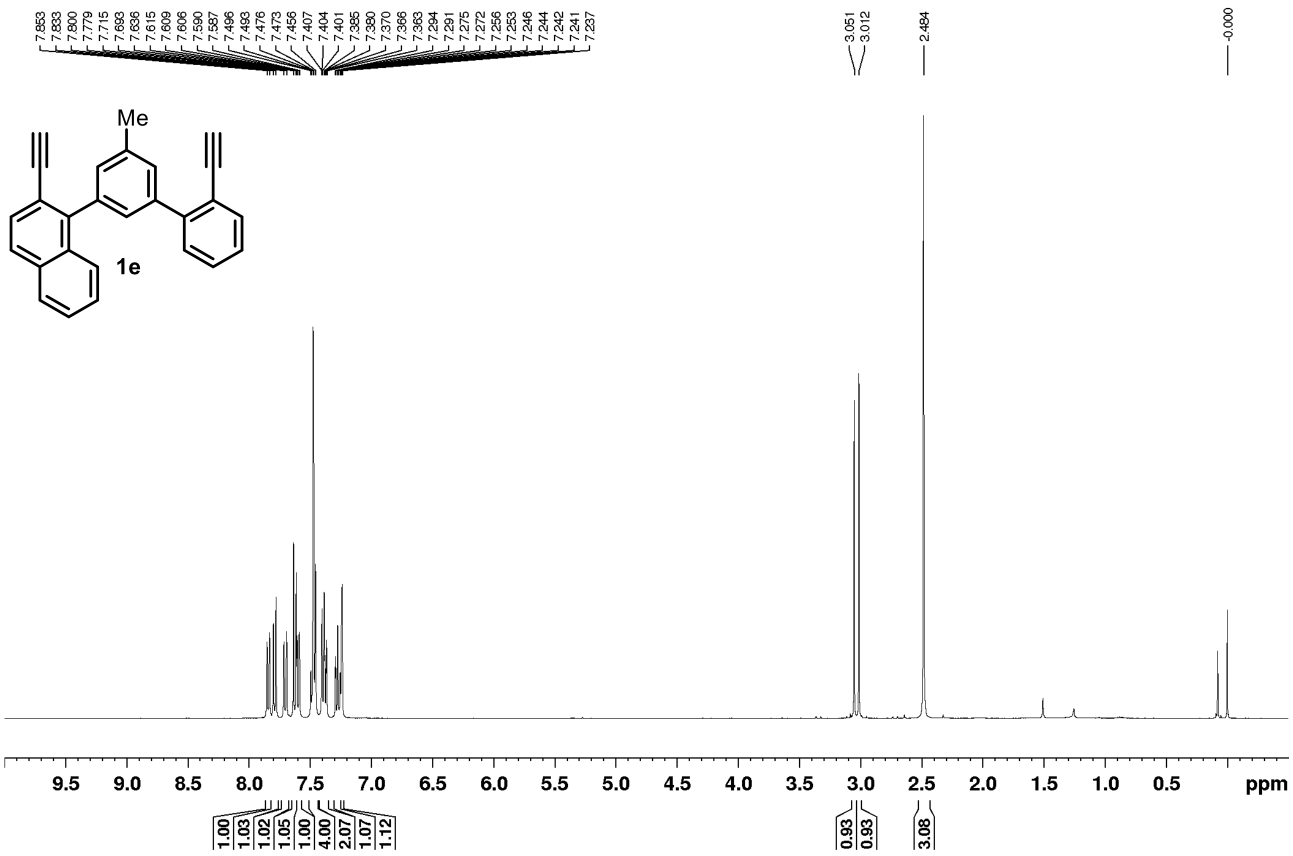

^{13}C NMR ($CDCl_3$, 100 MHz)

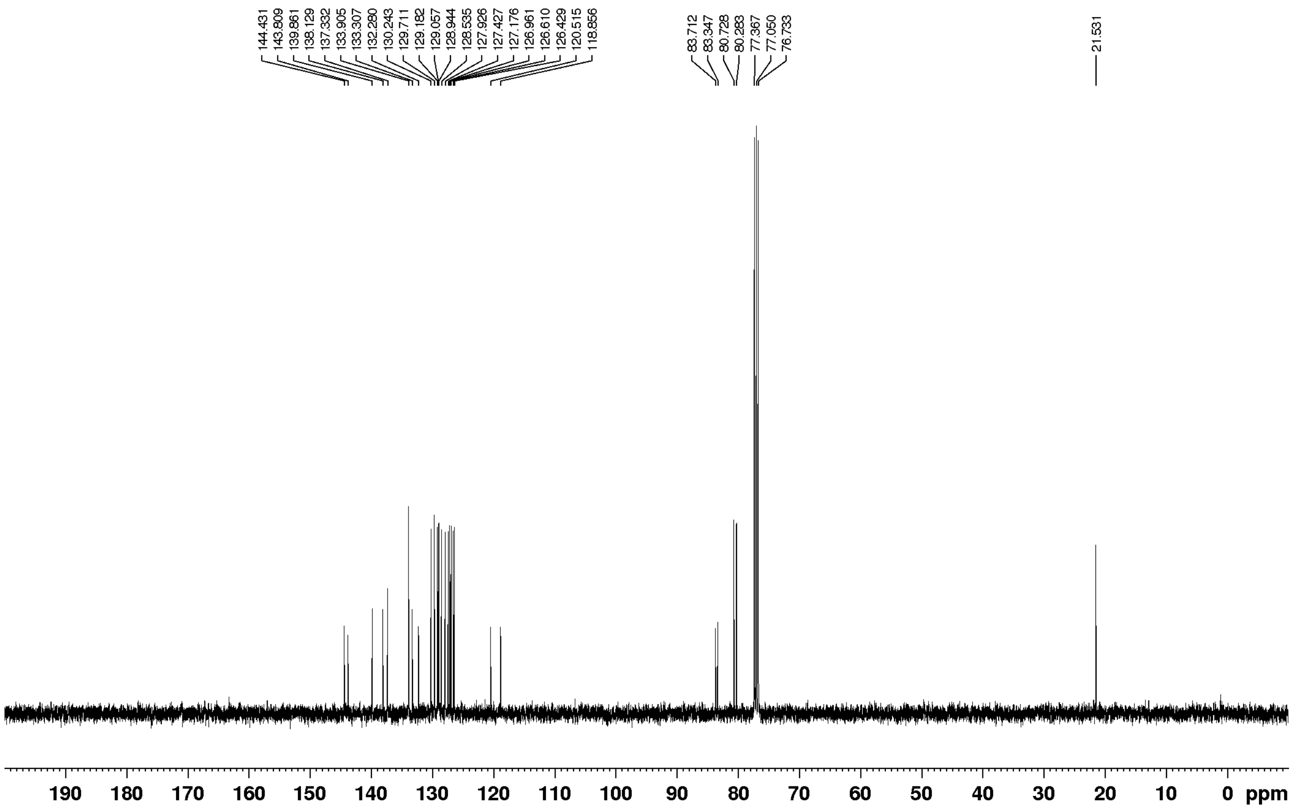

((1-(3-Bromo-5-chlorophenyl)naphthalen-2-yl)ethynyl)triisopropylsilane (S19)

^{1}H NMR ($CDCl_3$, 400 MHz)

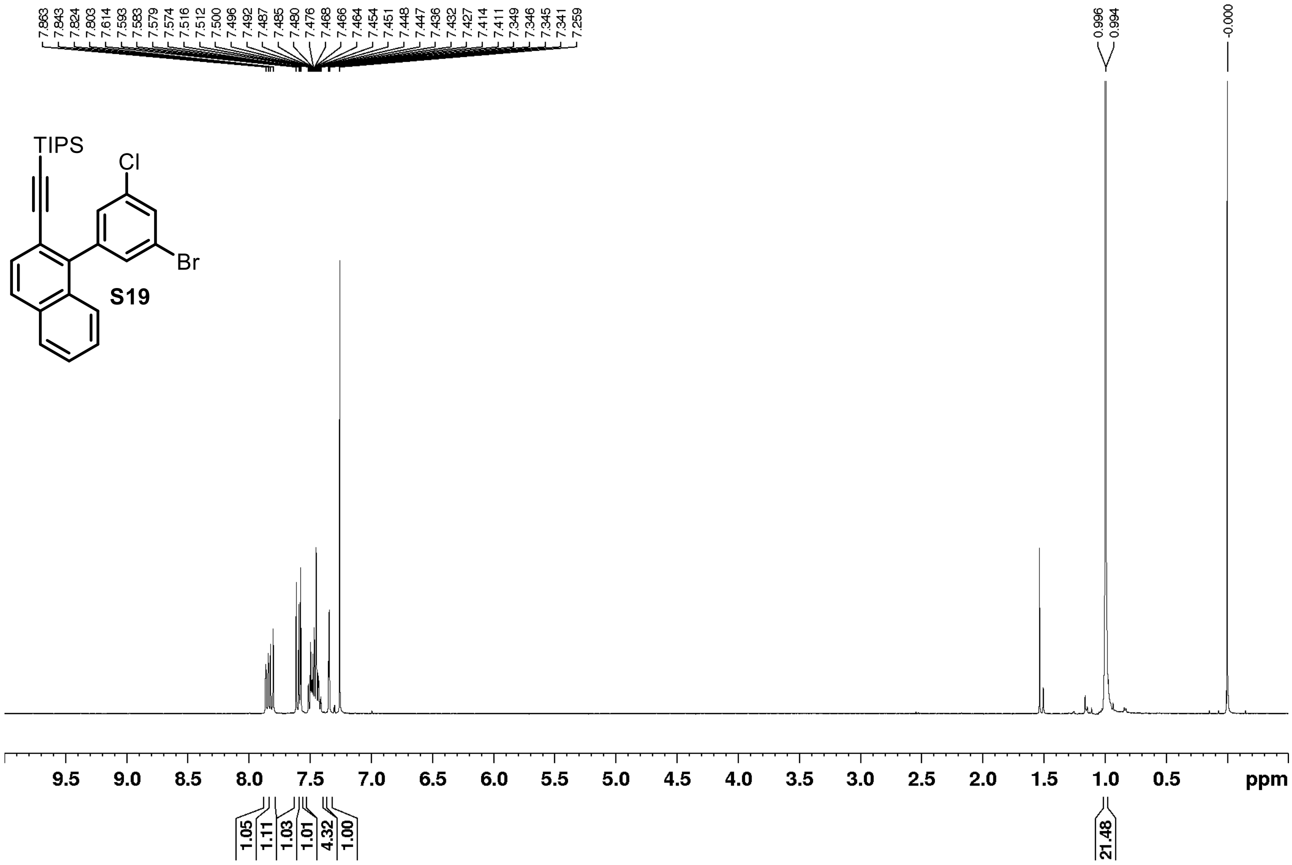

^{13}C NMR ($CDCl_3$, 100 MHz)

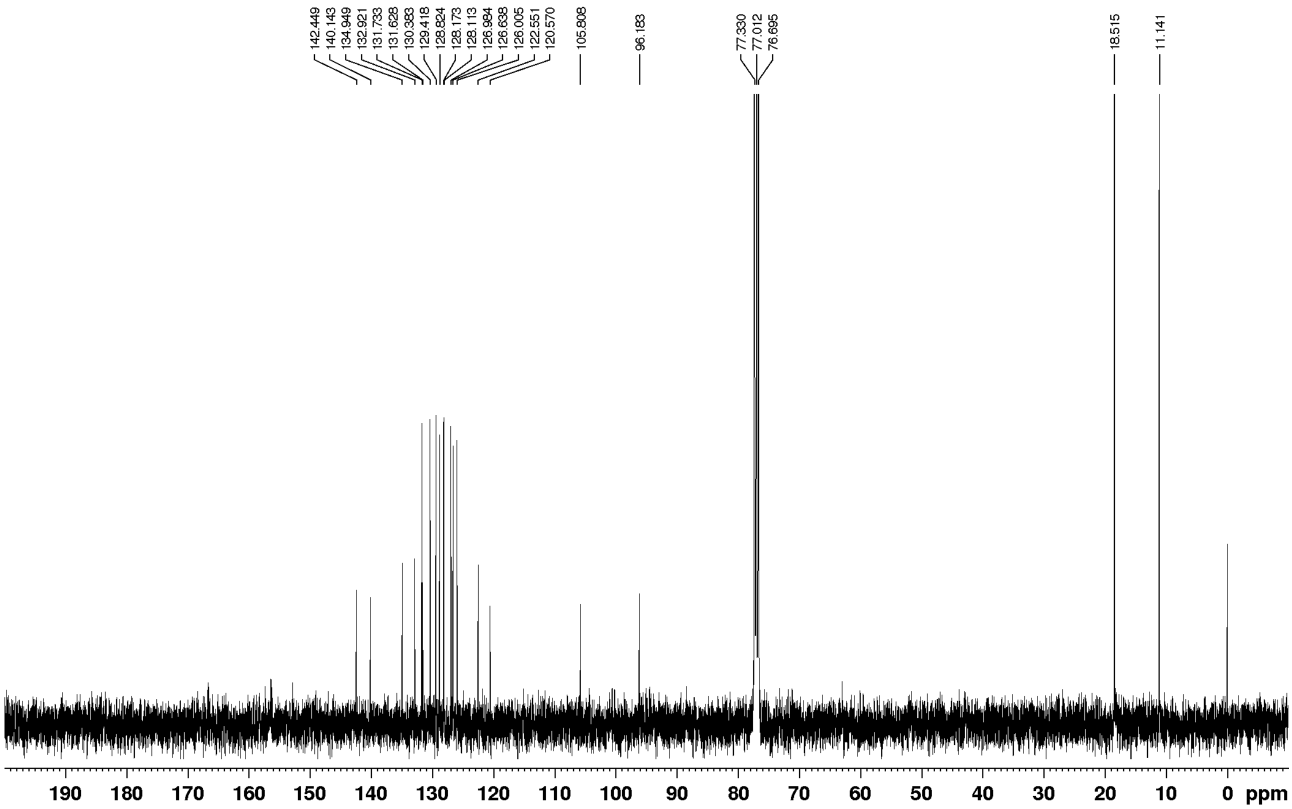

((1-(3-Chloro-5-(4,4,5,5-tetramethyl-1,3,2-dioxaborolan-2-yl)phenyl)naphthalen-2-yl)ethynyl)-triisopropylsilane (S20)

^{1}H NMR ($CDCl_3$, 400 MHz)

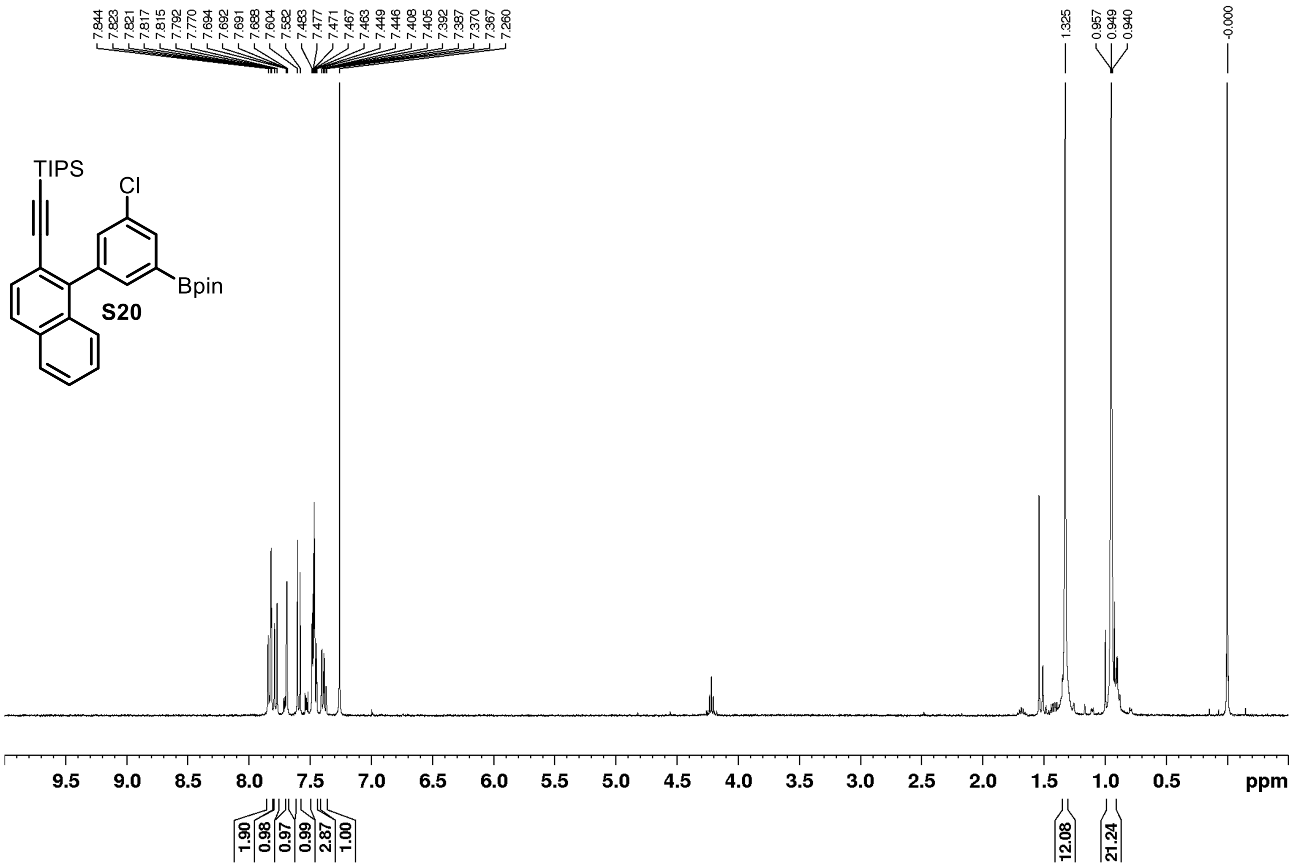

^{13}C NMR ($CDCl_3$, 100 MHz)

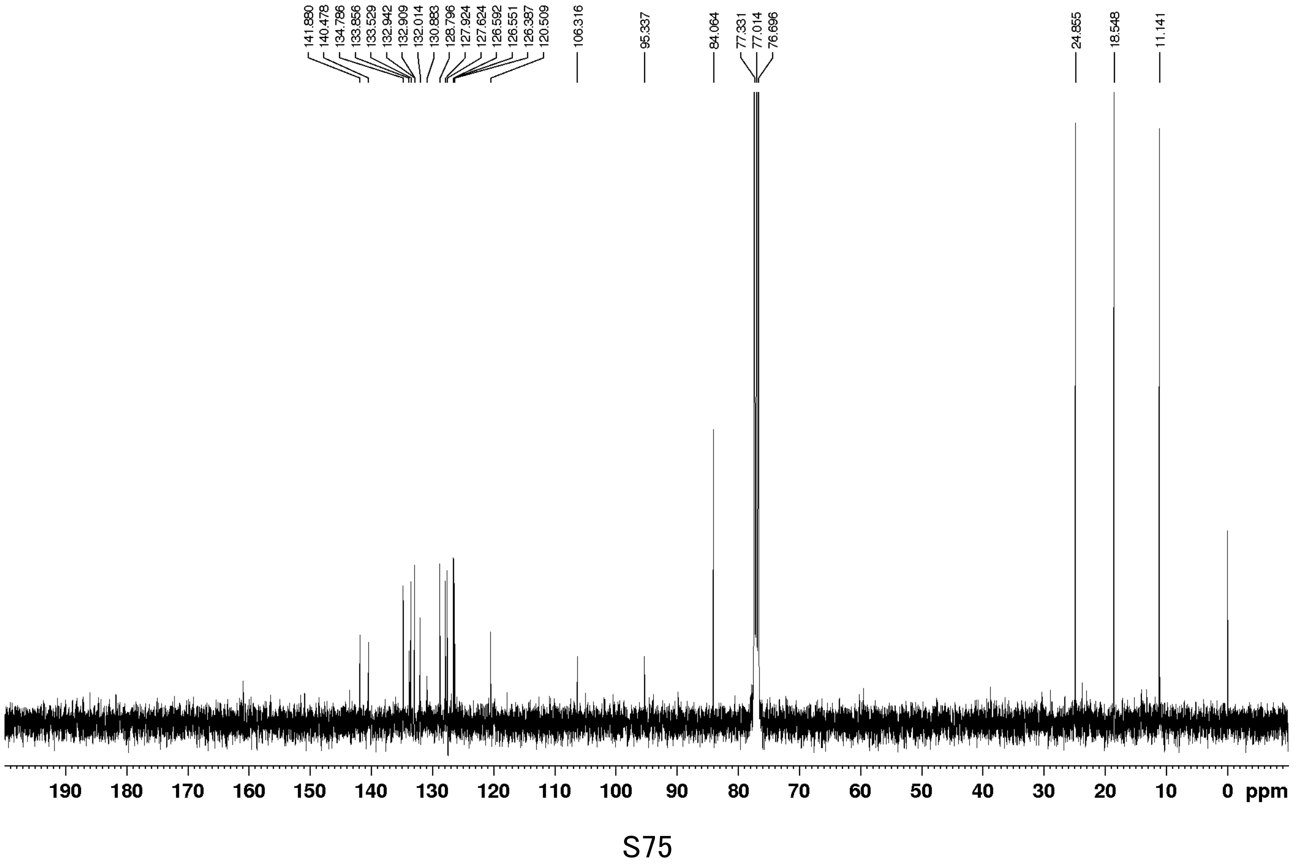

1-(3-Chloro-5-(1-ethynylnaphthalen-2-yl)phenyl)-2-ethynylnaphthalene (1f)

^{1}H NMR ($CDCl_3$, 400 MHz)

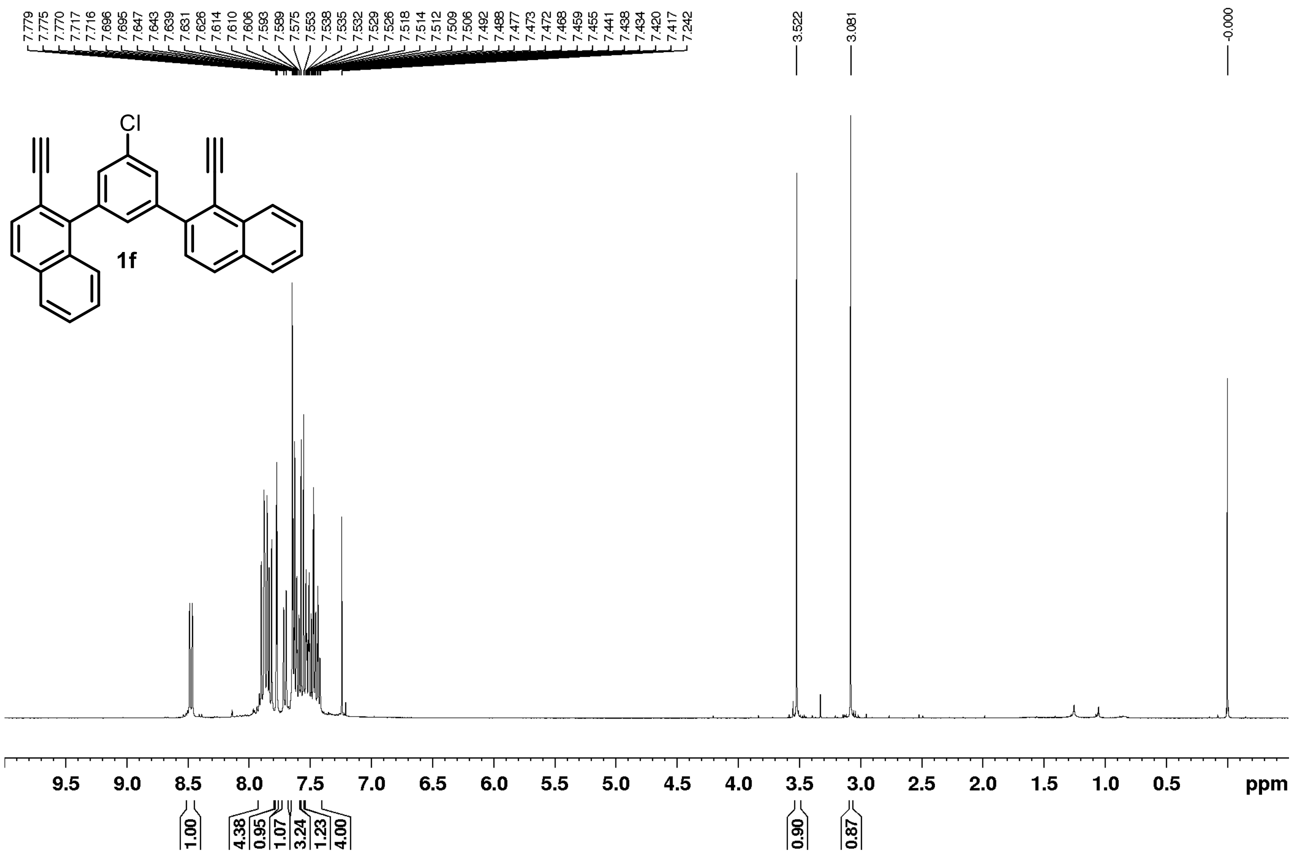

^{13}C NMR ($CDCl_3$, 100 MHz)

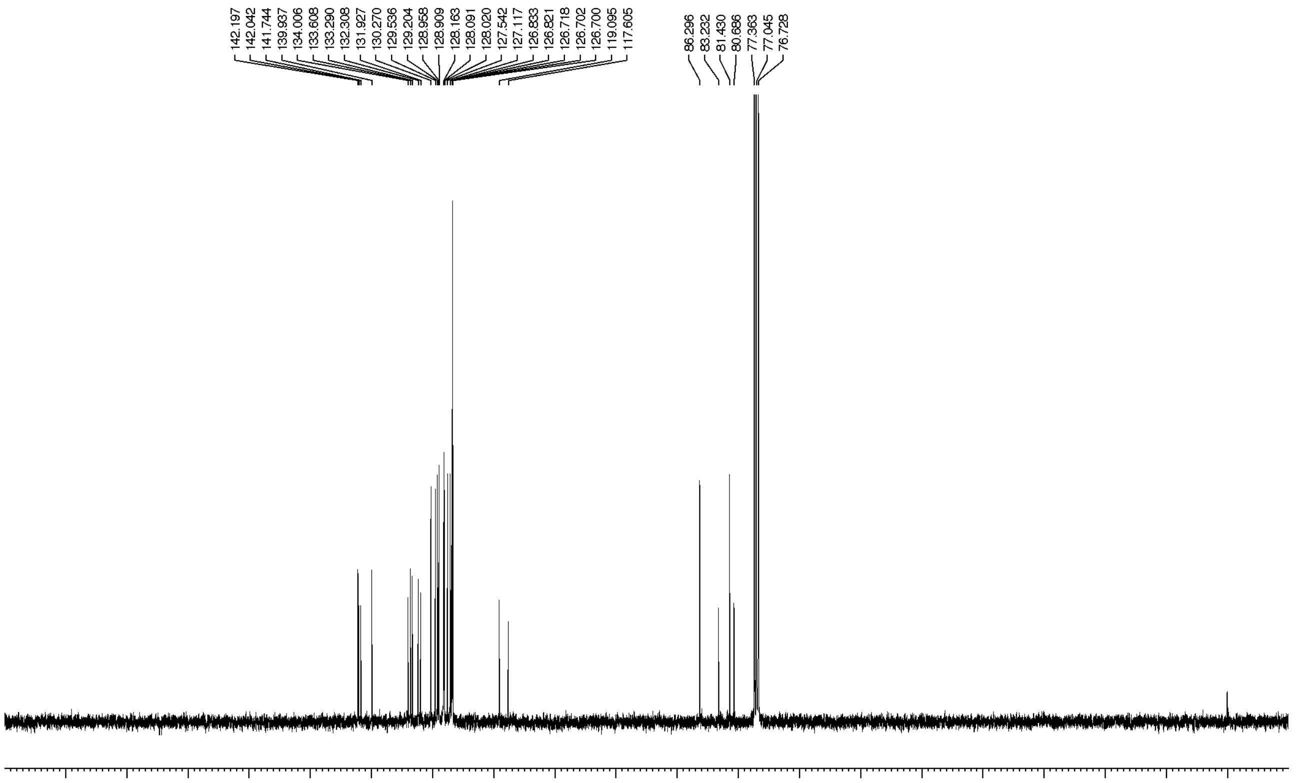

***o,m,o,p*-Tetraphenylene 3a and *o,m,o,m*-tetraphenylene 4a**

^{1}H NMR ($CDCl_3$, 400 MHz)

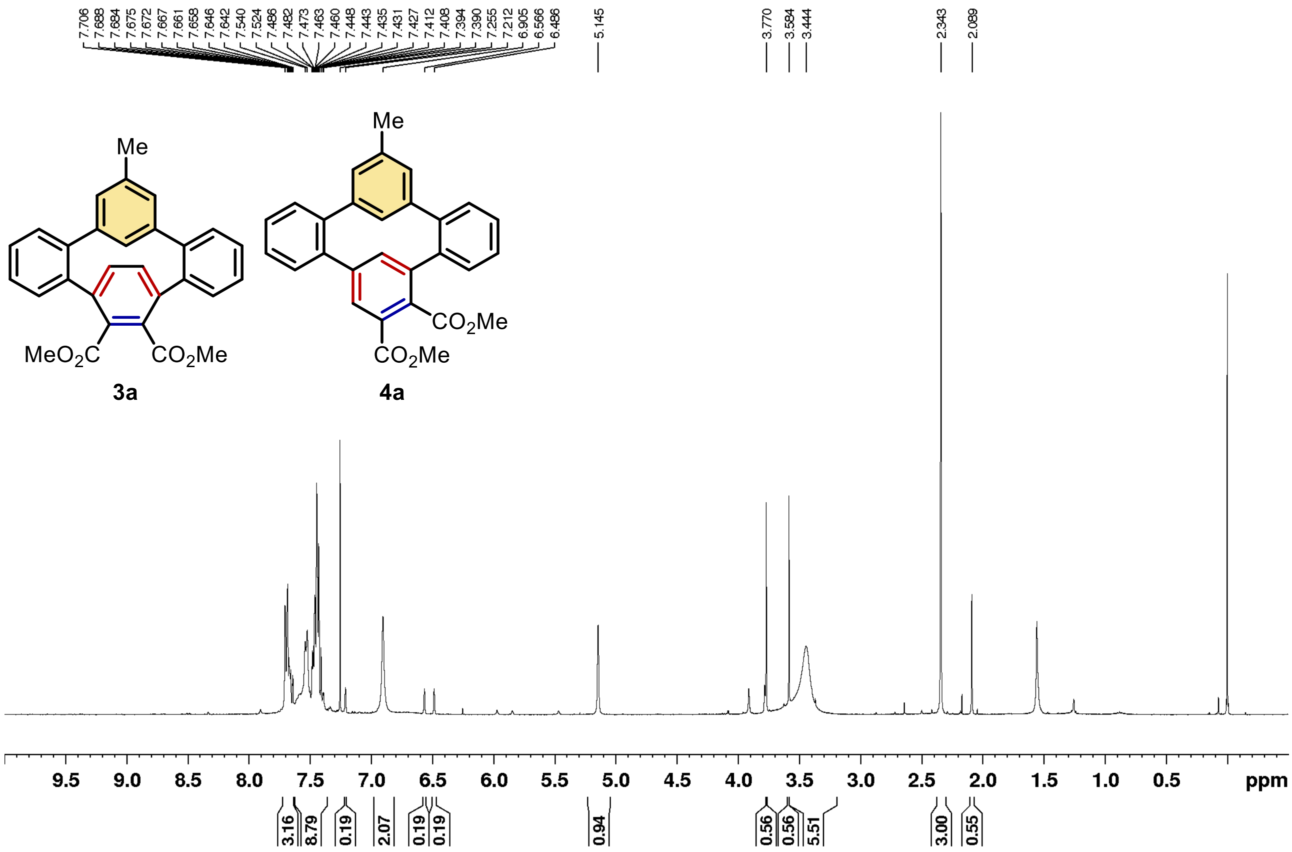

^{13}C NMR ($CDCl_3$, 100 MHz)

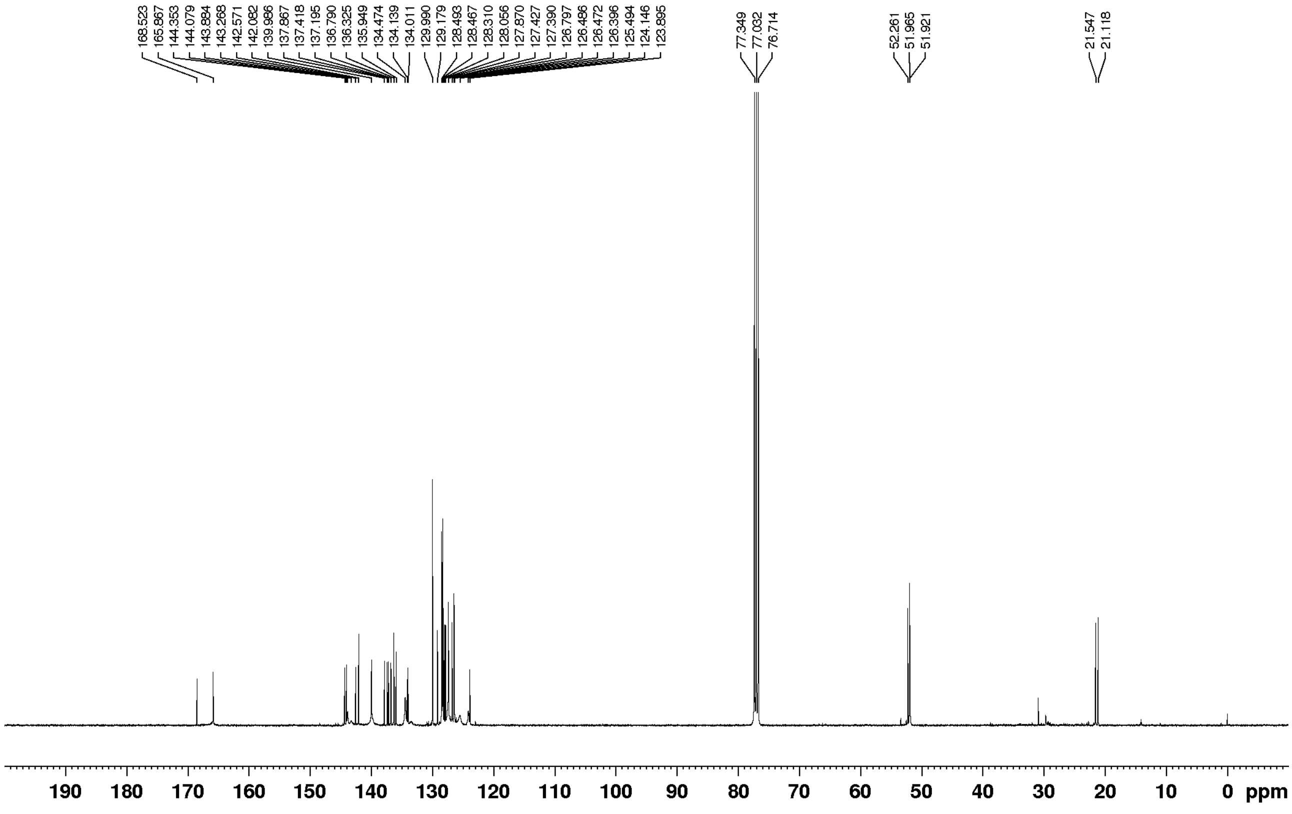

***o*,*m*,*o*,*p*-Tetraphenylene *cis*-3b and *o*,*m*,*o*,*m*-tetraphenylene 4b**

^{1}H NMR ($CDCl_3$, 400 MHz)

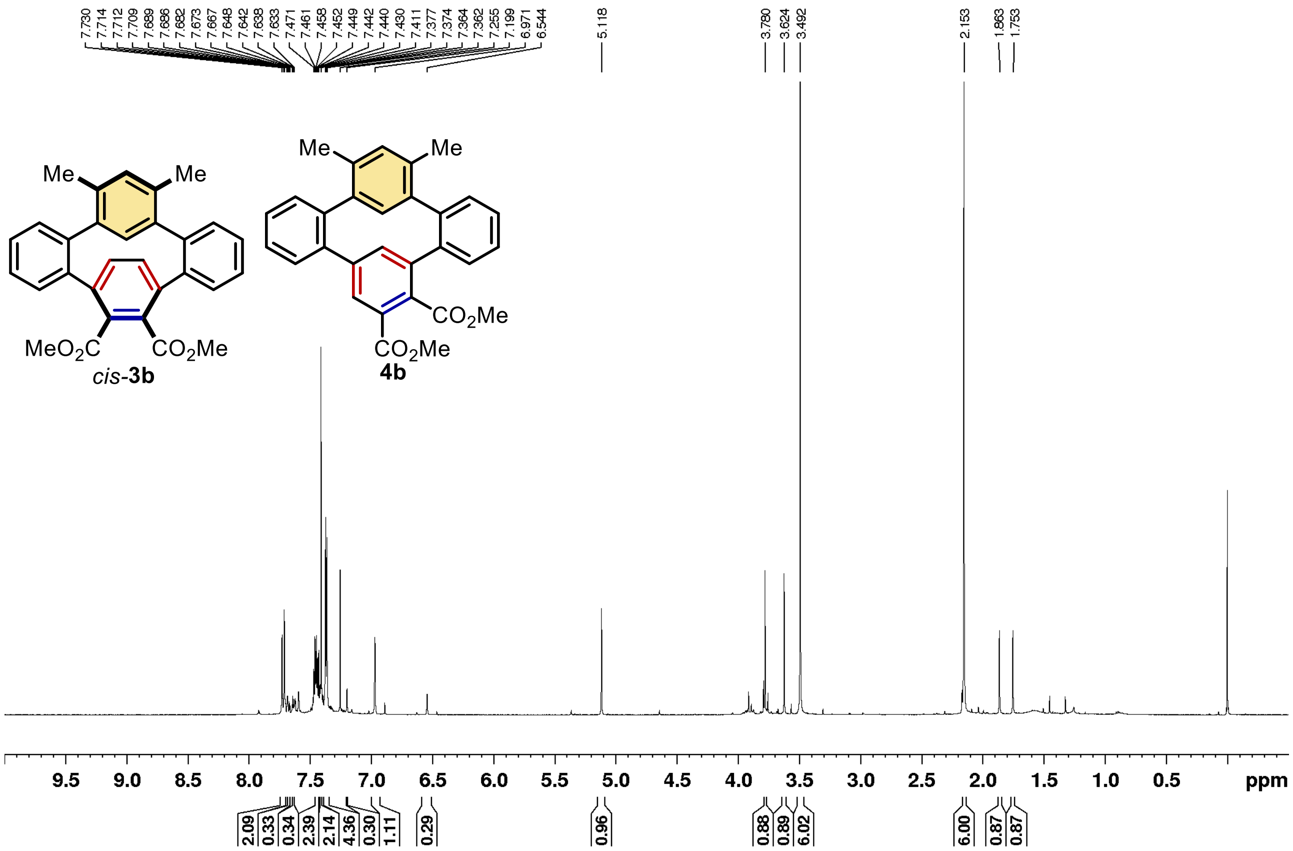

^{13}C NMR ($CDCl_3$, 100 MHz)

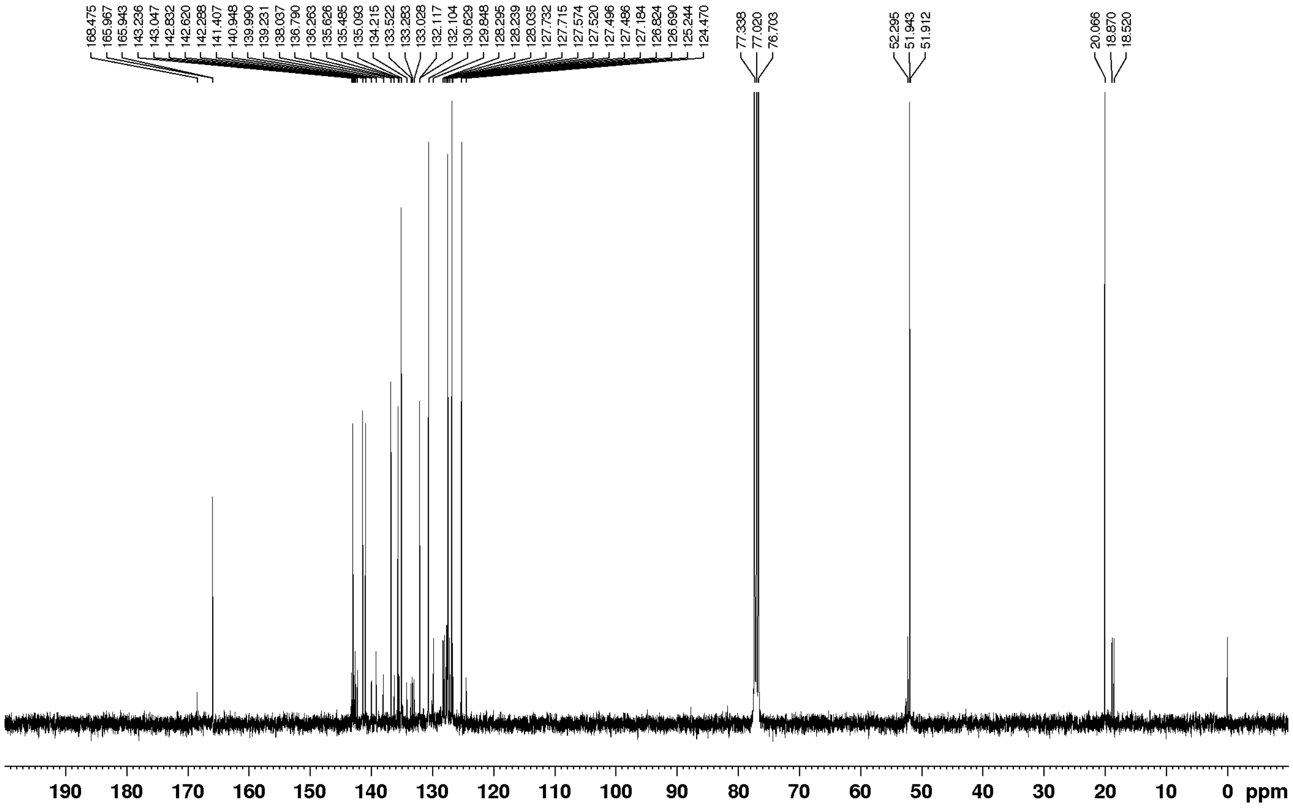

***o*,*m*,*o*,*p*-Tetraphenylene *cis*-3b**

^{1}H NMR ($CDCl_3$, 400 MHz)

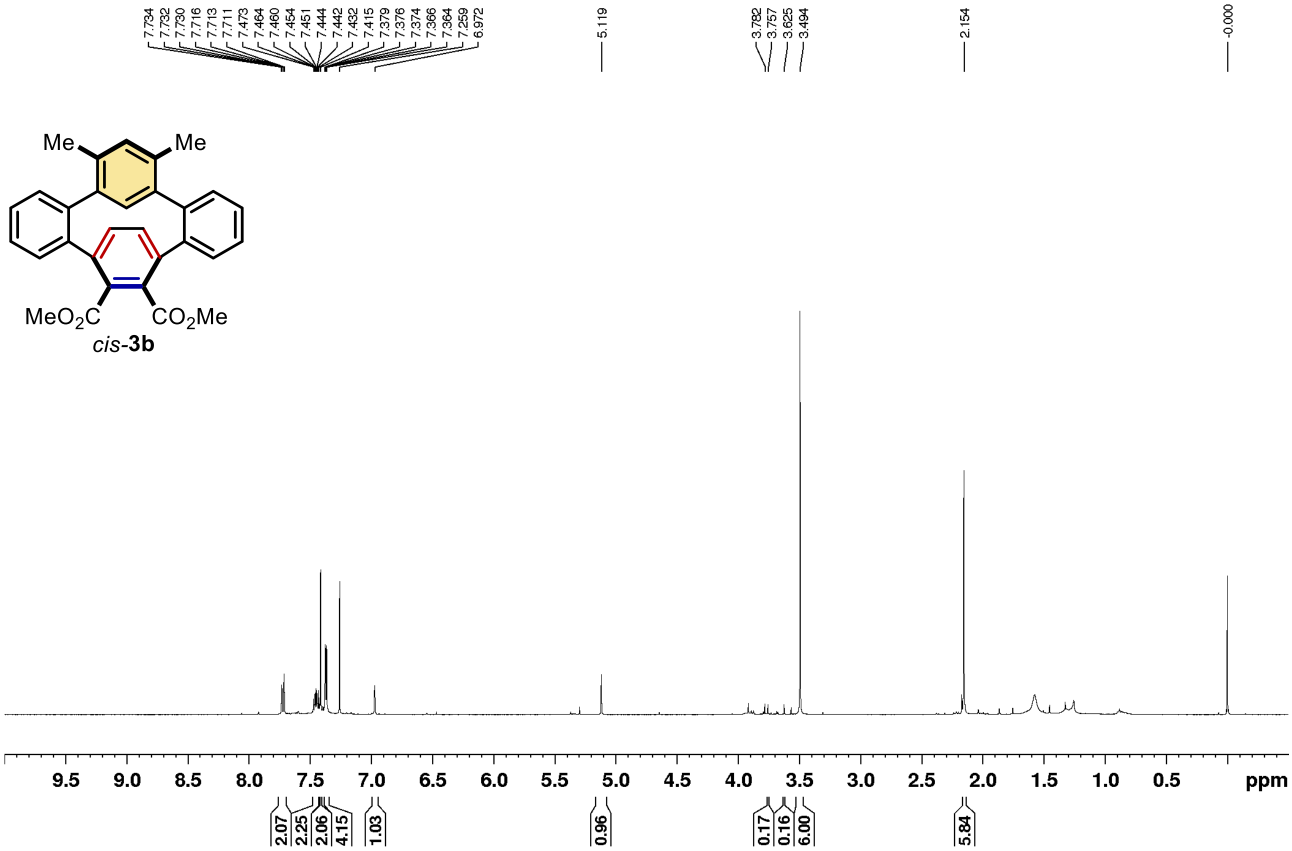

^{13}C NMR ($CDCl_3$, 100 MHz)

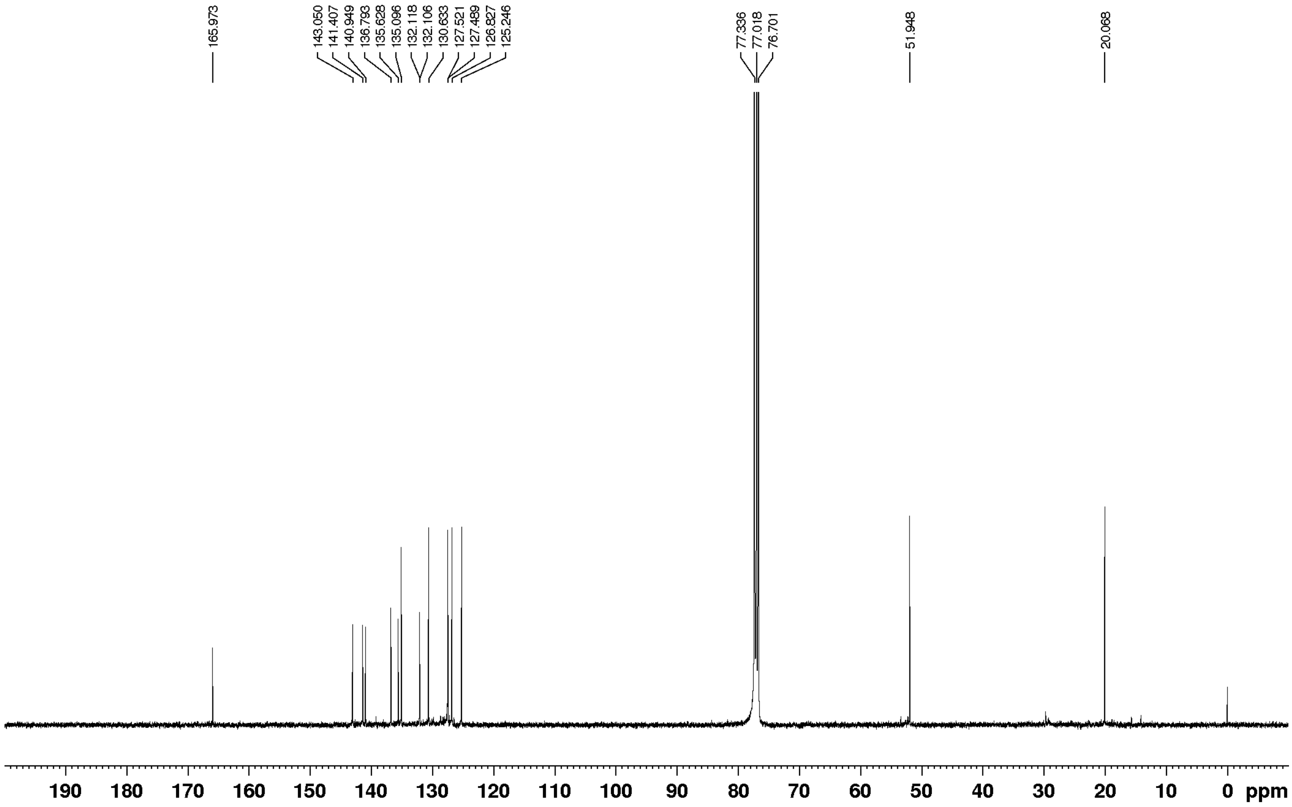

***o,m,o,p*-Tetraphenylene 3c**

^{1}H NMR ($CDCl_3$, 400 MHz)

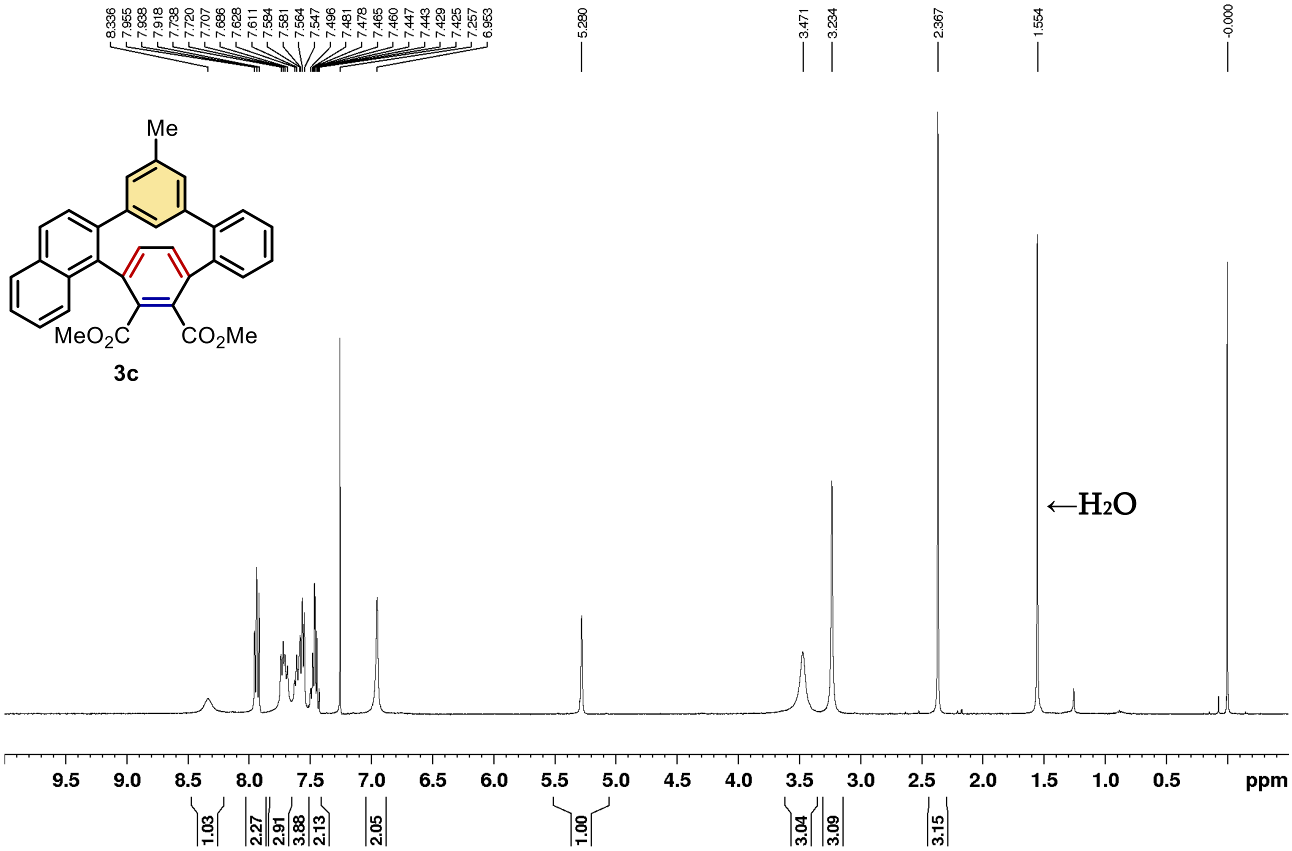

^{13}C NMR ($CDCl_3$, 100 MHz)

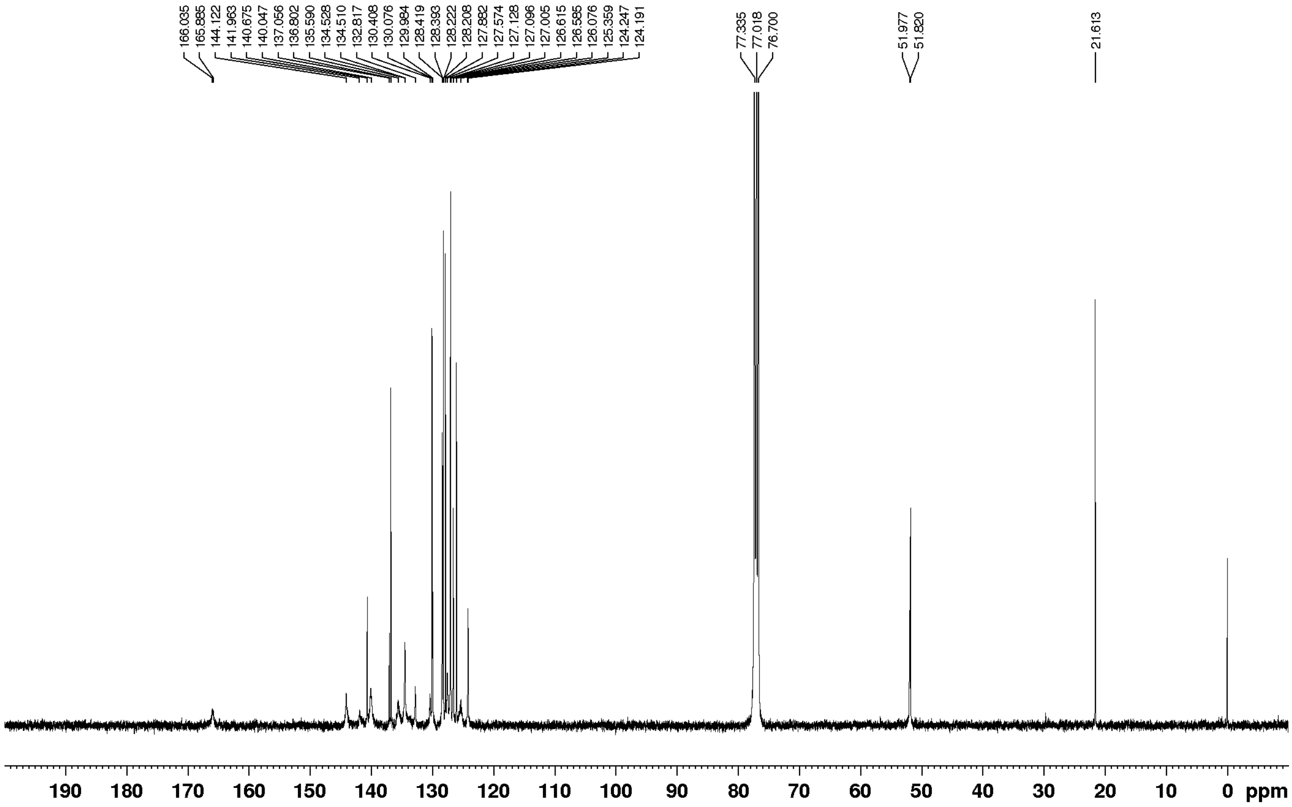

***o*,*m*,*o*,*p*-Tetraphenylene 3d and *o*,*m*,*o*,*m*-tetraphenylene 4d**

^{1}H NMR ($CDCl_3$, 400 MHz)

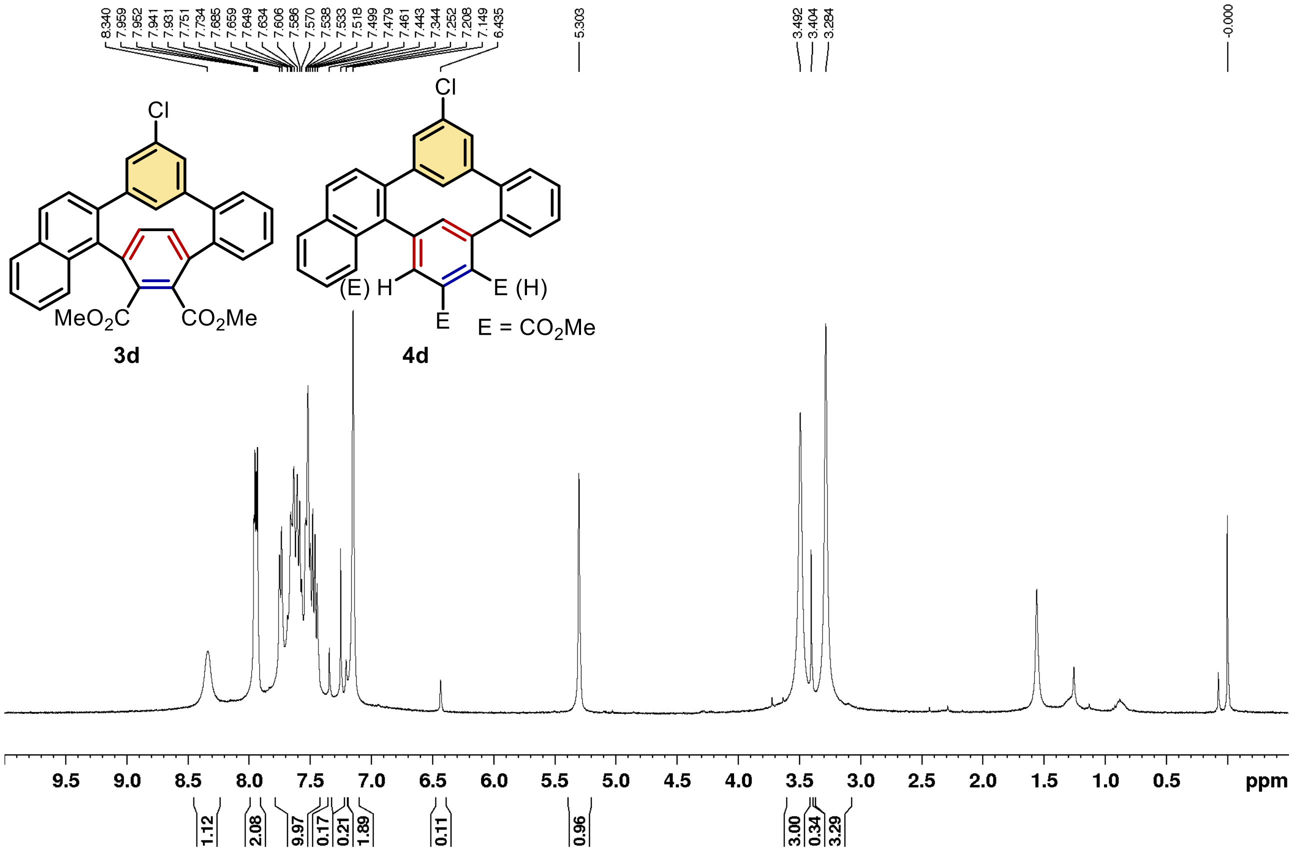

^{13}C NMR ($CDCl_3$, 100 MHz)

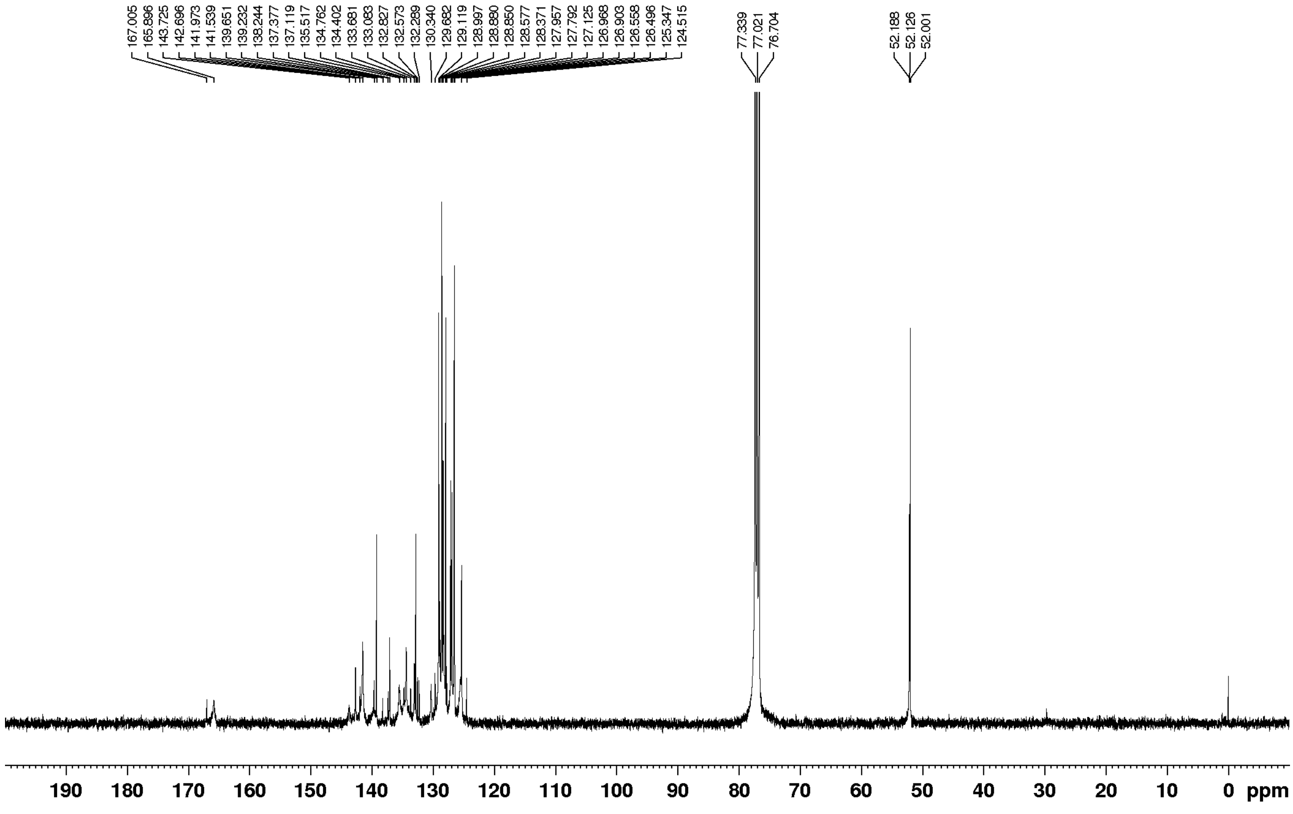

o,m,o,p-Tetraphenylenes *cis*-3e and *trans*-3e, and *o,m,o,m*-tetraphenylene 4e

^{1}H NMR ($CDCl_3$, 400 MHz)

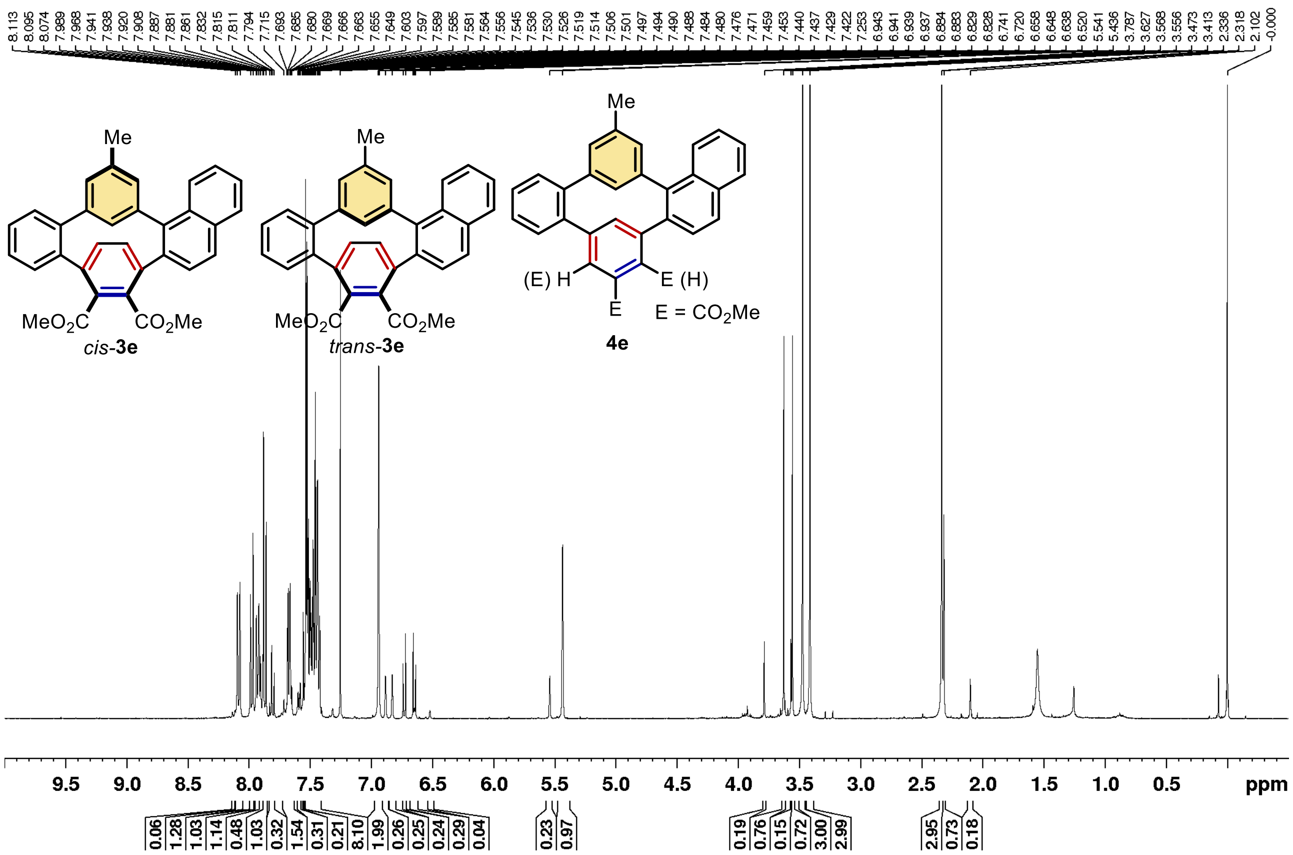

^{13}C NMR ($CDCl_3$, 100 MHz)

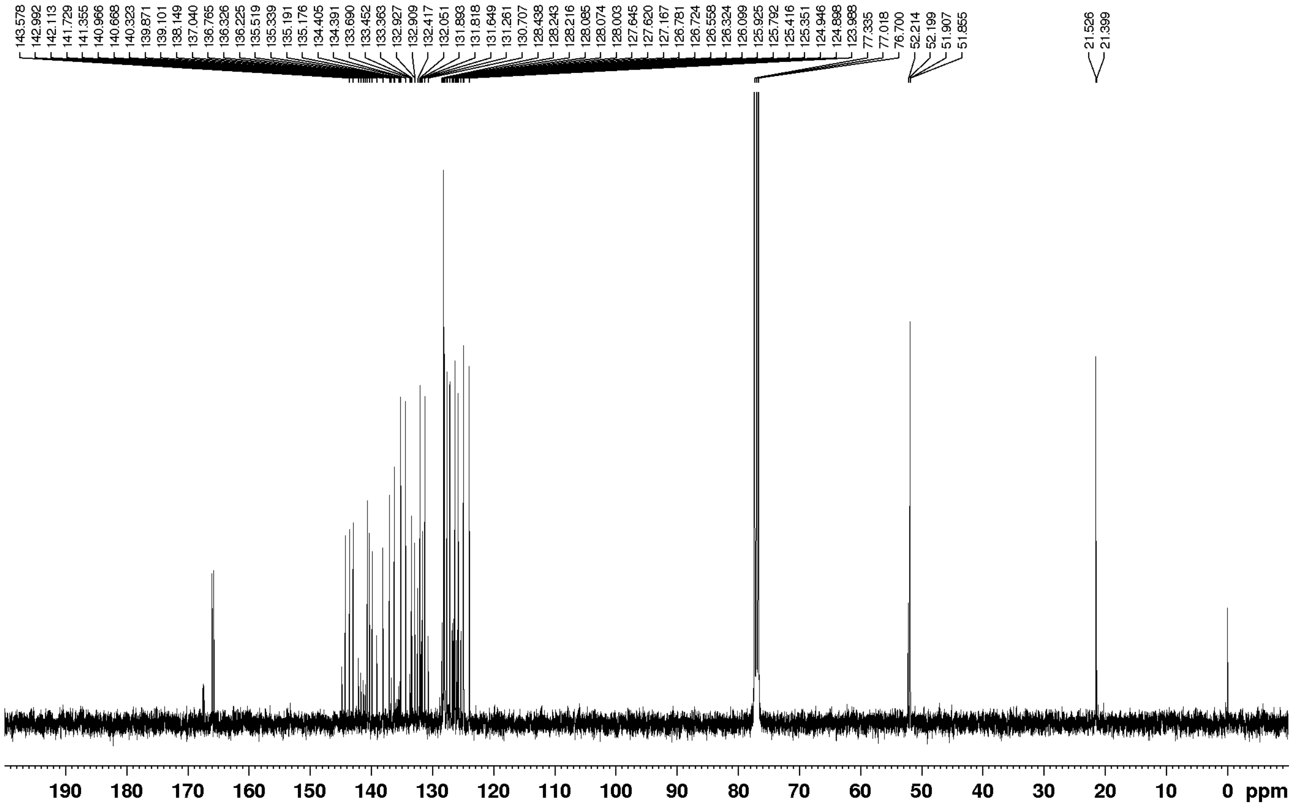

***o*,*m*,*o*,*p*-Tetraphenylenes *cis*-3f and *trans*-3f**

^{1}H NMR ($CDCl_3$, 400 MHz)

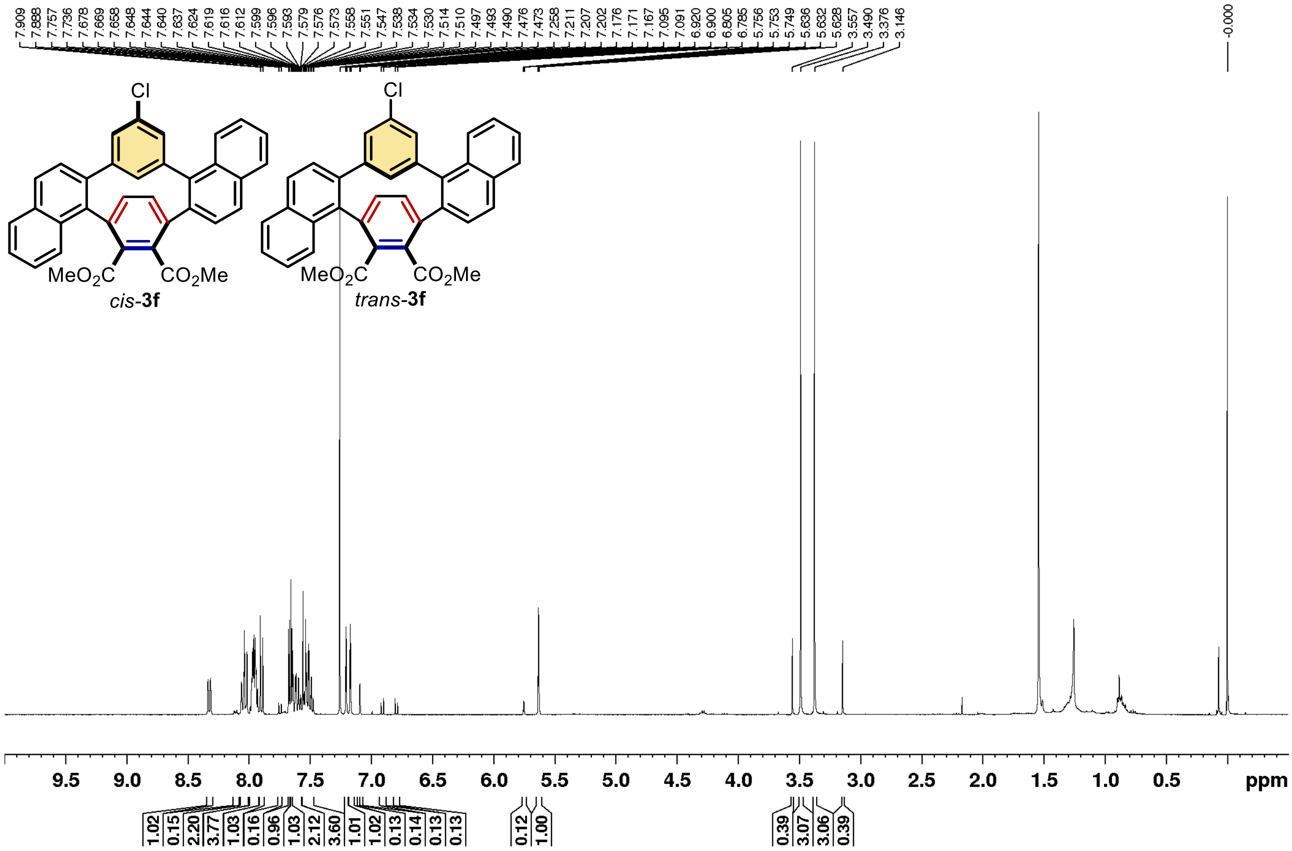

^{13}C NMR ($CDCl_3$, 100 MHz)

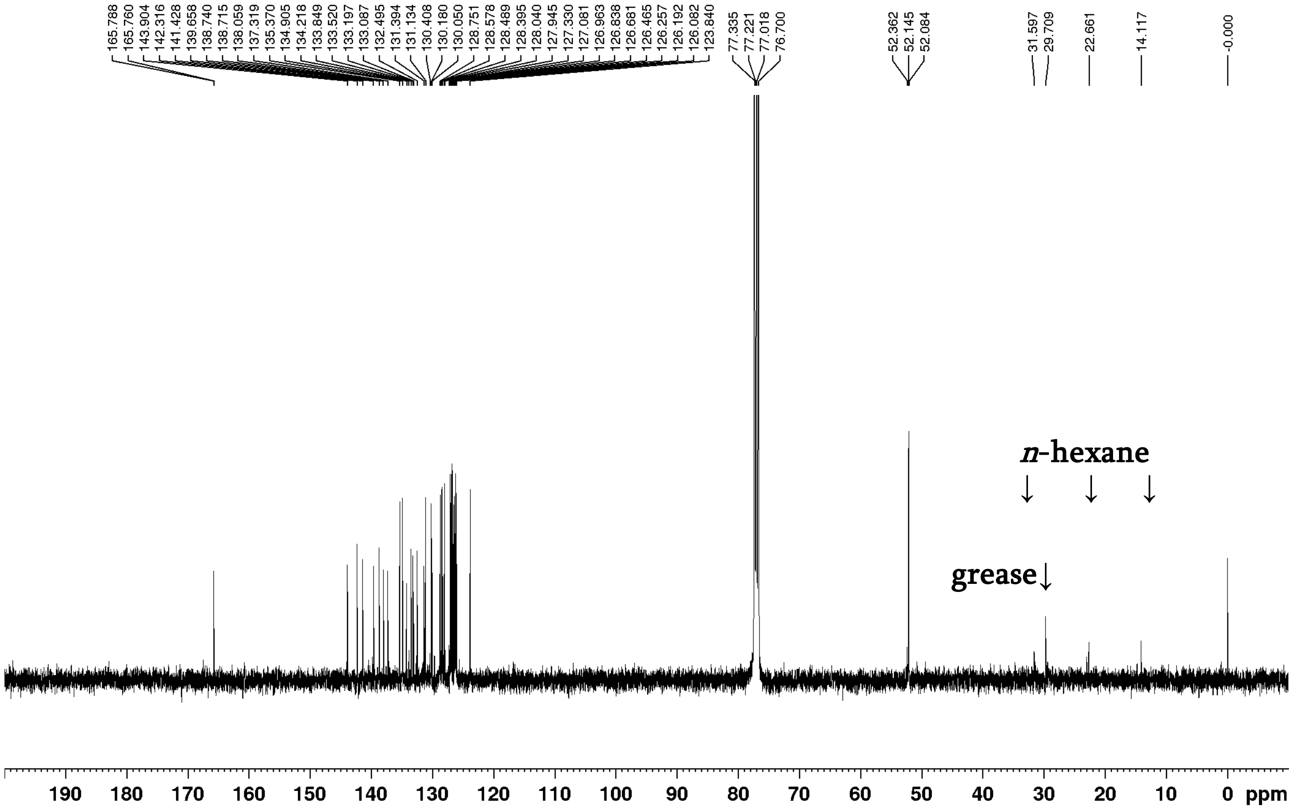

Supplement: Supplementary file 1 — ol4c02712_si_001.pdf [file ol4c02712_si_001.pdf]
